# Supplementary material for: Missing Value Imputation With Adversarial Random Forests—MissARF
Source: Stat Med. 2026 Feb 4;45(3-5):e70379. doi: 10.1002/sim.70379 (PMC12871009; doi:10.1002/sim.70379)
Supplement: Supplementary file 1 — Data S1: Supporting Information. [file SIM-45-0-s001.pdf]

Supplementary material for: Missing value imputation with adversarial random forests – MissARF

Golchian et al.

Contents

1 Setting I: Single imputation **2**
1.1 NRMSE 2
1.1.1 Linear effect 2
1.1.2 Squared effect 12
1.2 Brier Score 22
1.2.1 Linear effect 22
1.2.2 Squared effect 32

2 Setting II: Multiple imputation **42**
2.1 Coverage rate 42
2.1.1 Category 1: Similar performance across all methods, MissARF with smallest average width 42
2.1.2 Category 2: PMM methods struggle, MissARF performs well 54
2.1.3 Category 3: All methods perform poorly 58
2.2 Average CI Width 62
2.2.1 Category 1: Similar performance across all methods, MissARF with smallest average width 62
2.2.2 Category 2: PMM methods struggle, MissARF performs well 74
2.2.3 Category 3: All methods perform poorly 78
2.3 RMSE 82
2.3.1 Category 1: Similar performance across all methods, MissARF with smallest average width 82
2.3.2 Category 2: PMM methods struggle, MissARF performs well 94
2.3.3 Category 3: All methods perform poorly 98

3 Real Data **102**

4 Runtime comparisons **103**

5 Further experiments **106**
5.1 Possible hyperparameter tuning 106
5.2 Smaller sample sizes 106

1 Setting I: Single imputation

1.1 NRMSE

1.1.1 Linear effect

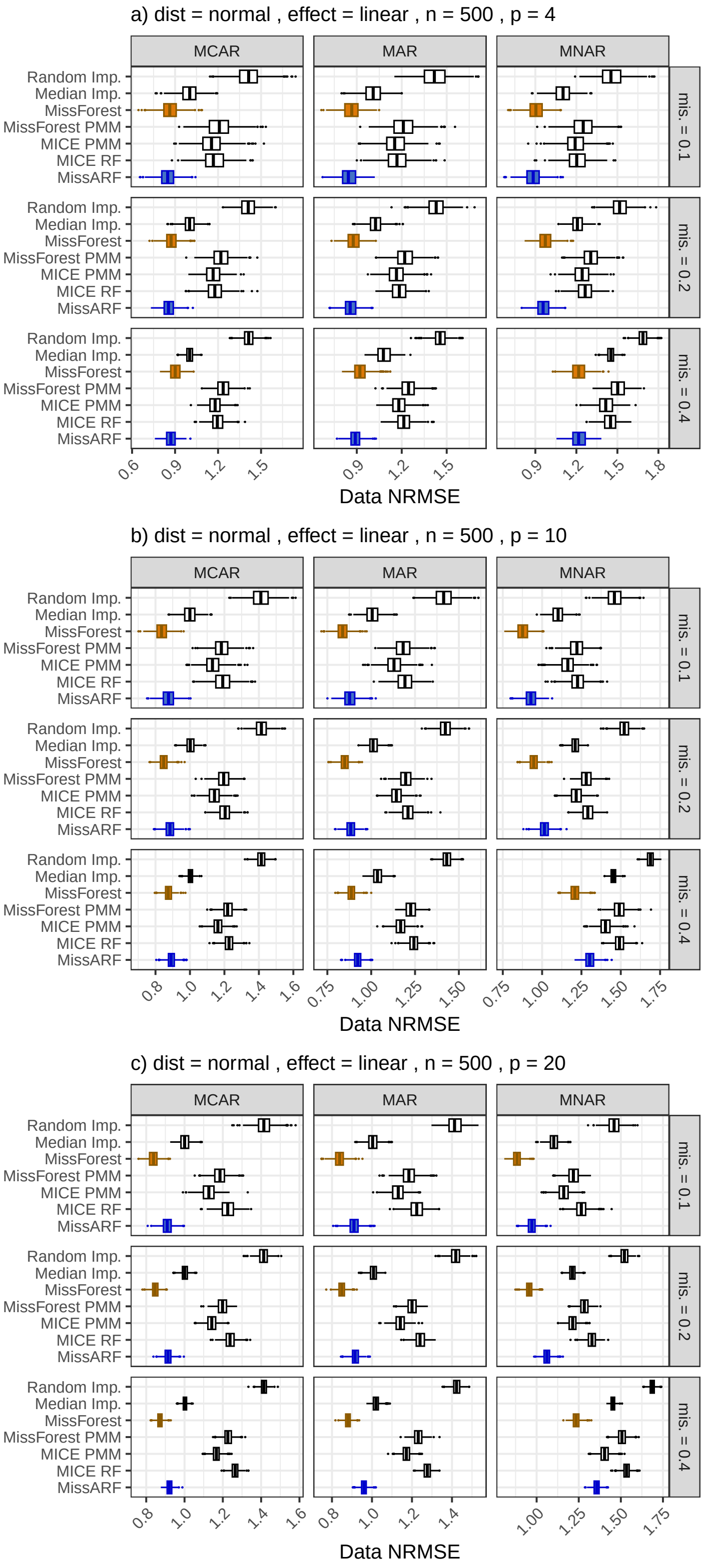

Figure S1: **NRMSE** of the normal distribution setting with a linear effect over different missingness patterns, dimensionality (p) and missingness rates (mis.) with $n = 500$. The boxplots are plotted over the replicates, with MissARF (blue) and MissForest (orange) highlighted.

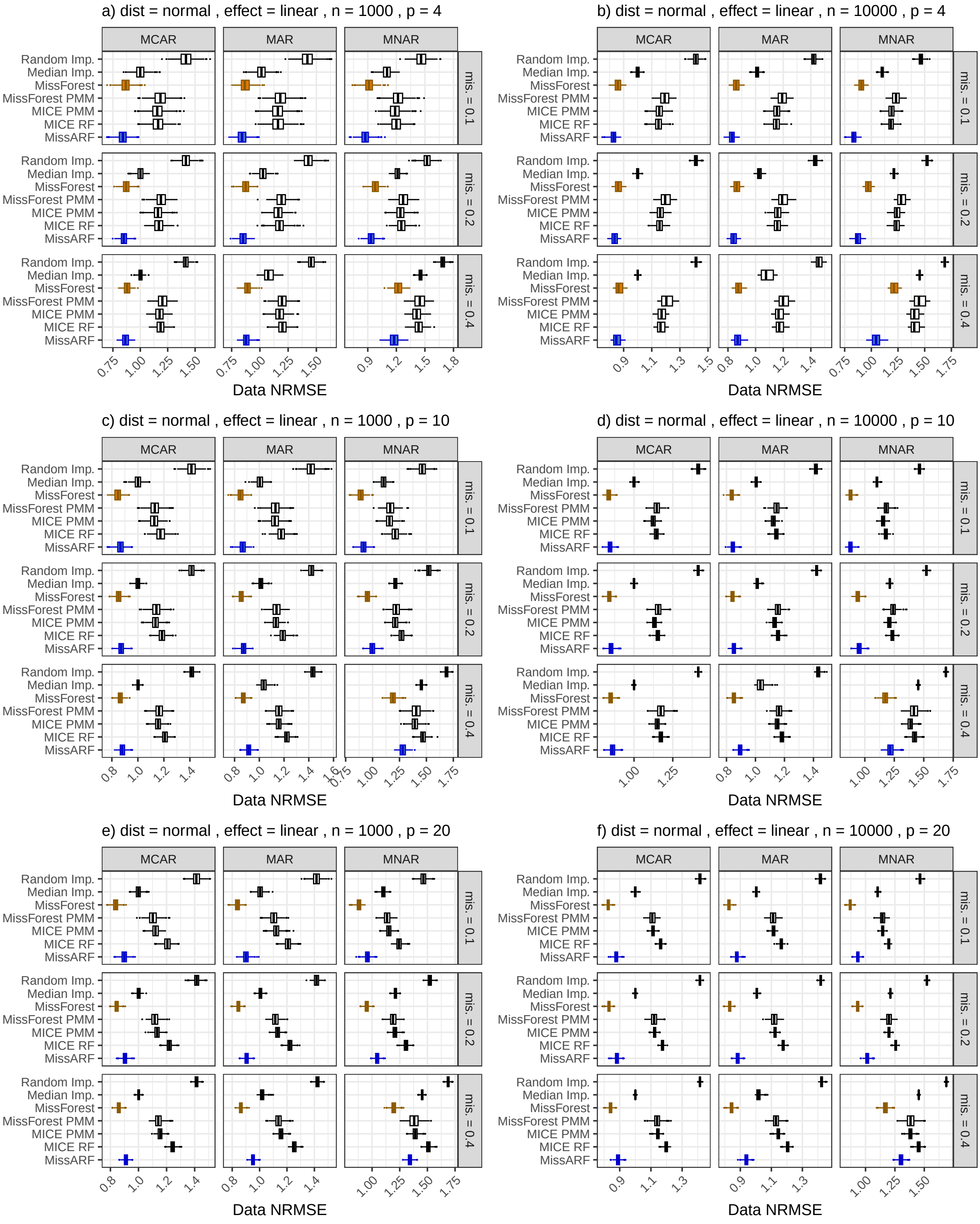

Figure S2: **NRMSE** of the normal distribution setting with a linear effect over different missingness patterns, dimensionality (p) and missingness rates (mis.) with $n = 1000$ (left) and $n = 10,000$ (right). The boxplots are plotted over the replicates, with MissARF (blue) and MissForest (orange) highlighted.

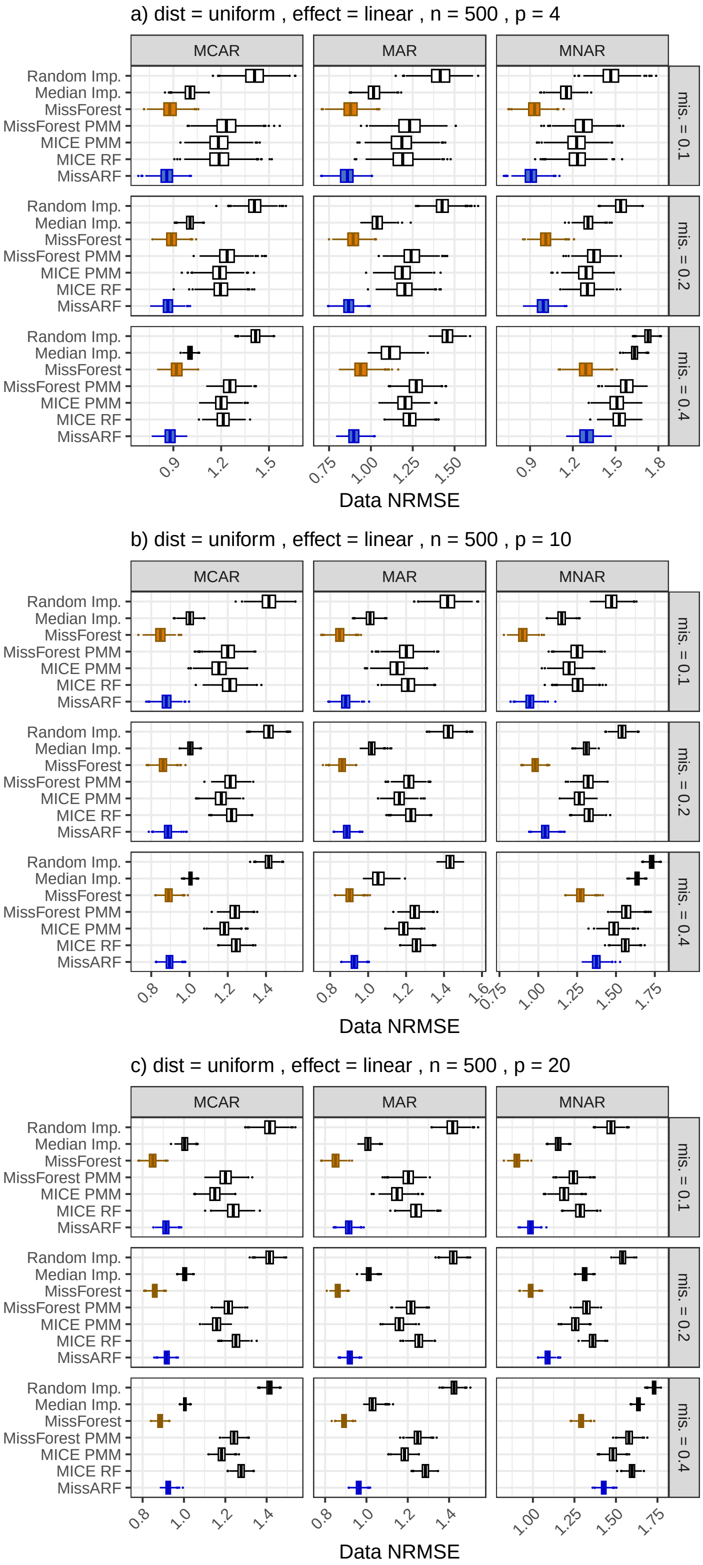

Figure S3: **NRMSE** of the uniform distribution setting with a linear effect over different missingness patterns, dimensionality (p) and missingness rates (mis.) with $n = 500$. The boxplots are plotted over the replicates, with MissARF (blue) and MissForest (orange) highlighted.

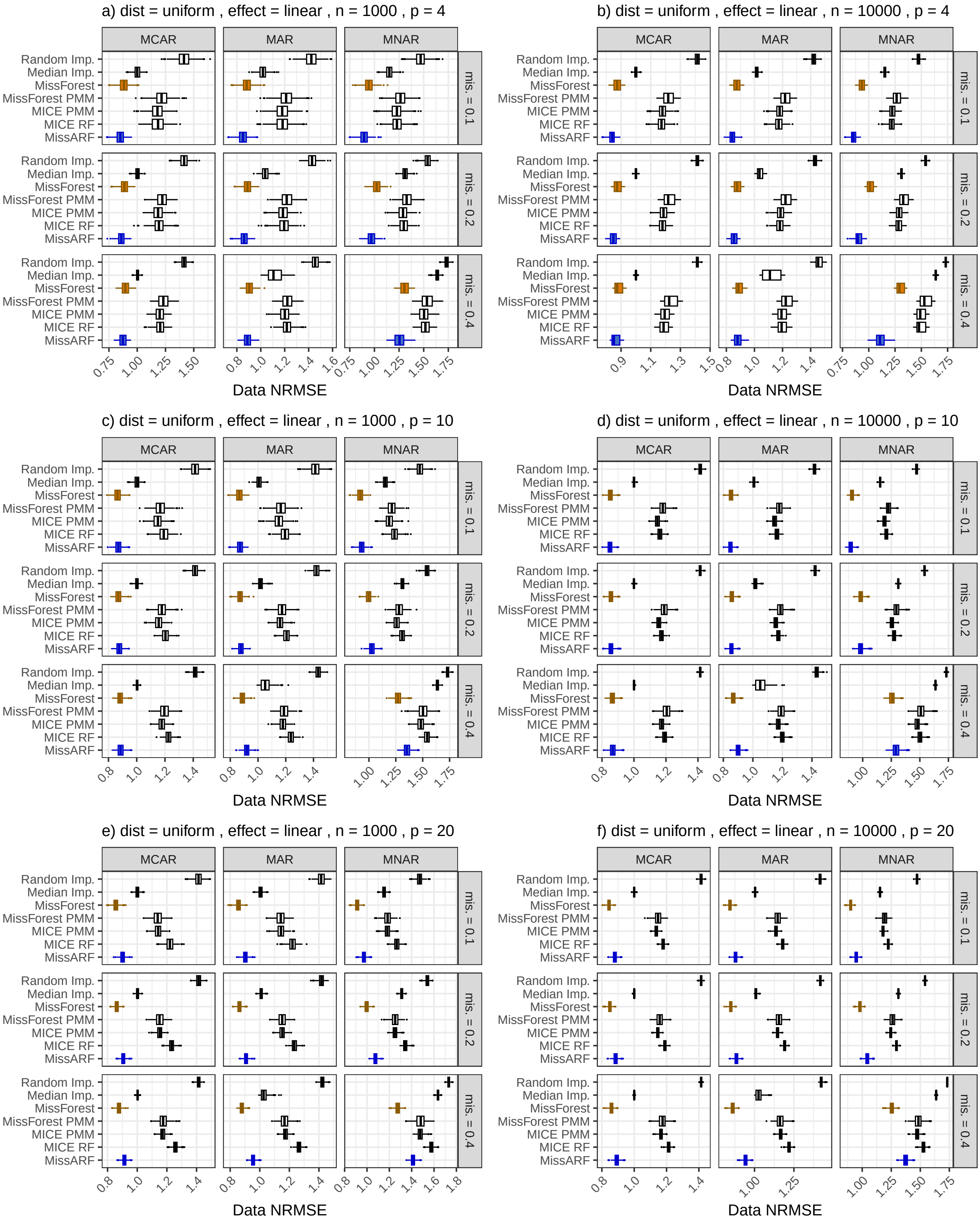

Figure S4: **NRMSE** of the uniform distribution setting with a linear effect over different missingness patterns, dimensionality (p) and missingness rates (mis.) with $n = 1000$ (left) and $n = 10,000$ (right). The boxplots are plotted over the replicates, with MissARF (blue) and MissForest (orange) highlighted.

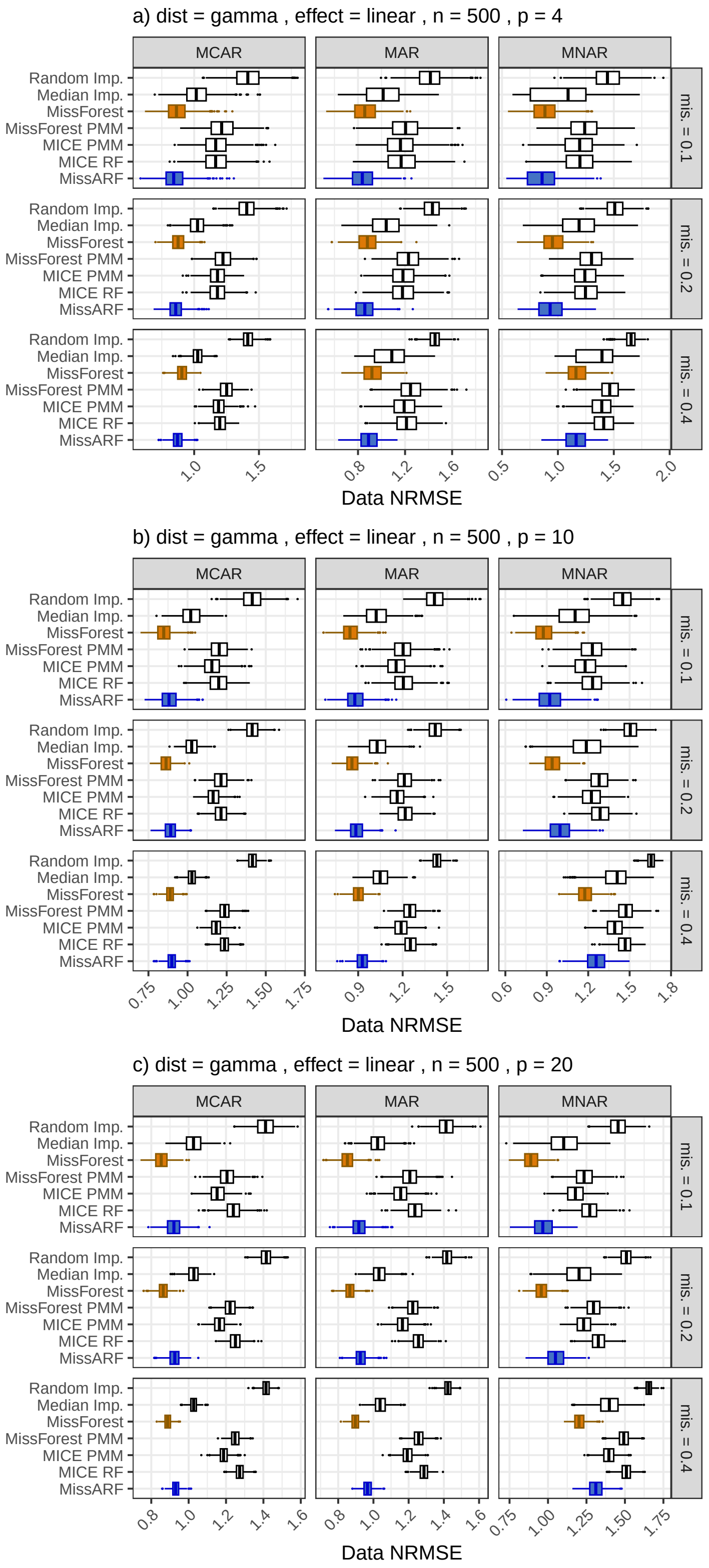

Figure S5: **NRMSE** of the gamma distribution setting with a linear effect over different missingness patterns, dimensionality (p) and missingness rates (mis.) with $n = 500$. The boxplots are plotted over the replicates, with MissARF (blue) and MissForest (orange) highlighted.

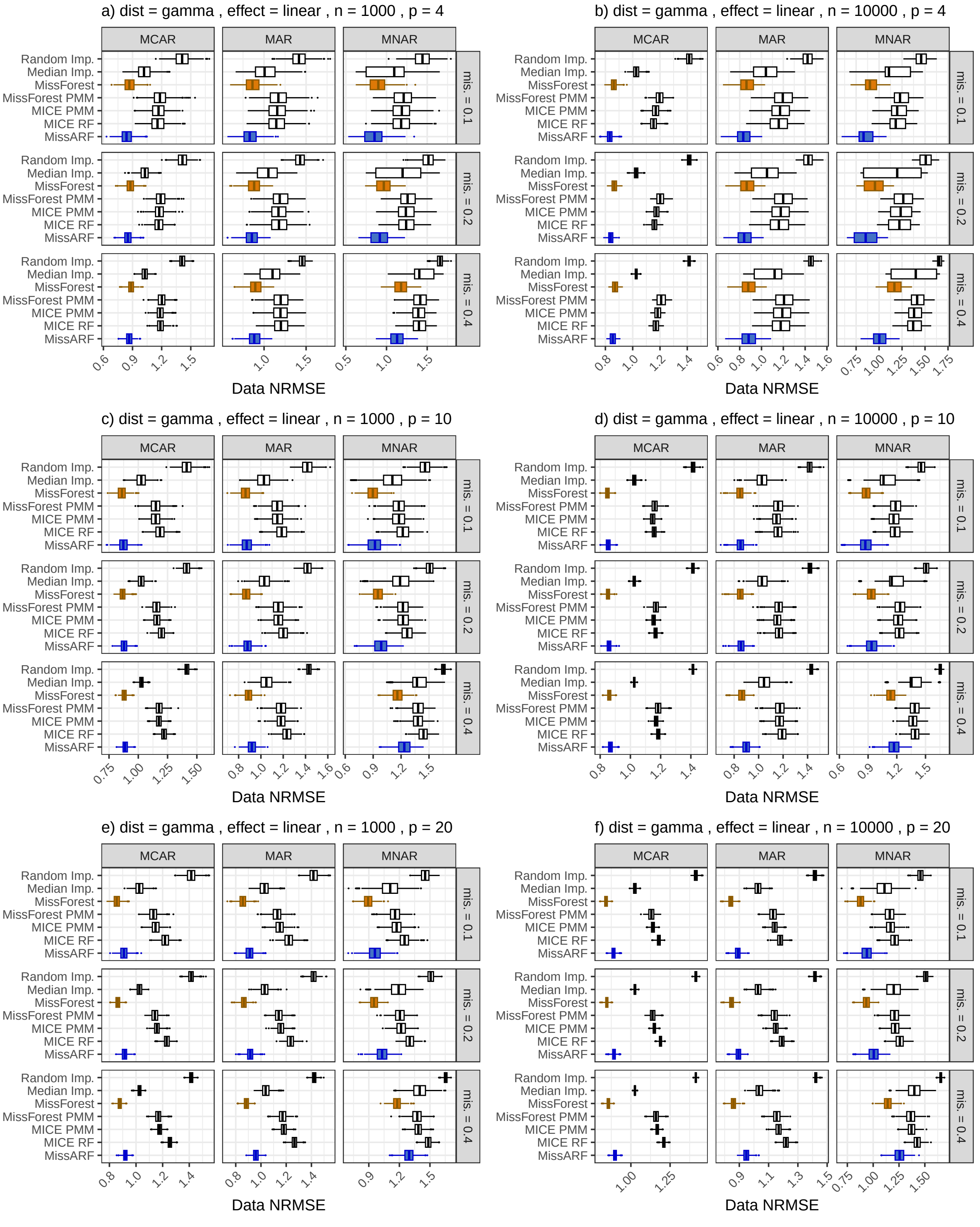

Figure S6: **NRMSE** of the gamma distribution setting with a linear effect over different missingness patterns, dimensionality (p) and missingness rates (mis.) with $n = 1000$ (left) and $n = 10,000$ (right). The boxplots are plotted over the replicates, with MissARF (blue) and MissForest (orange) highlighted.

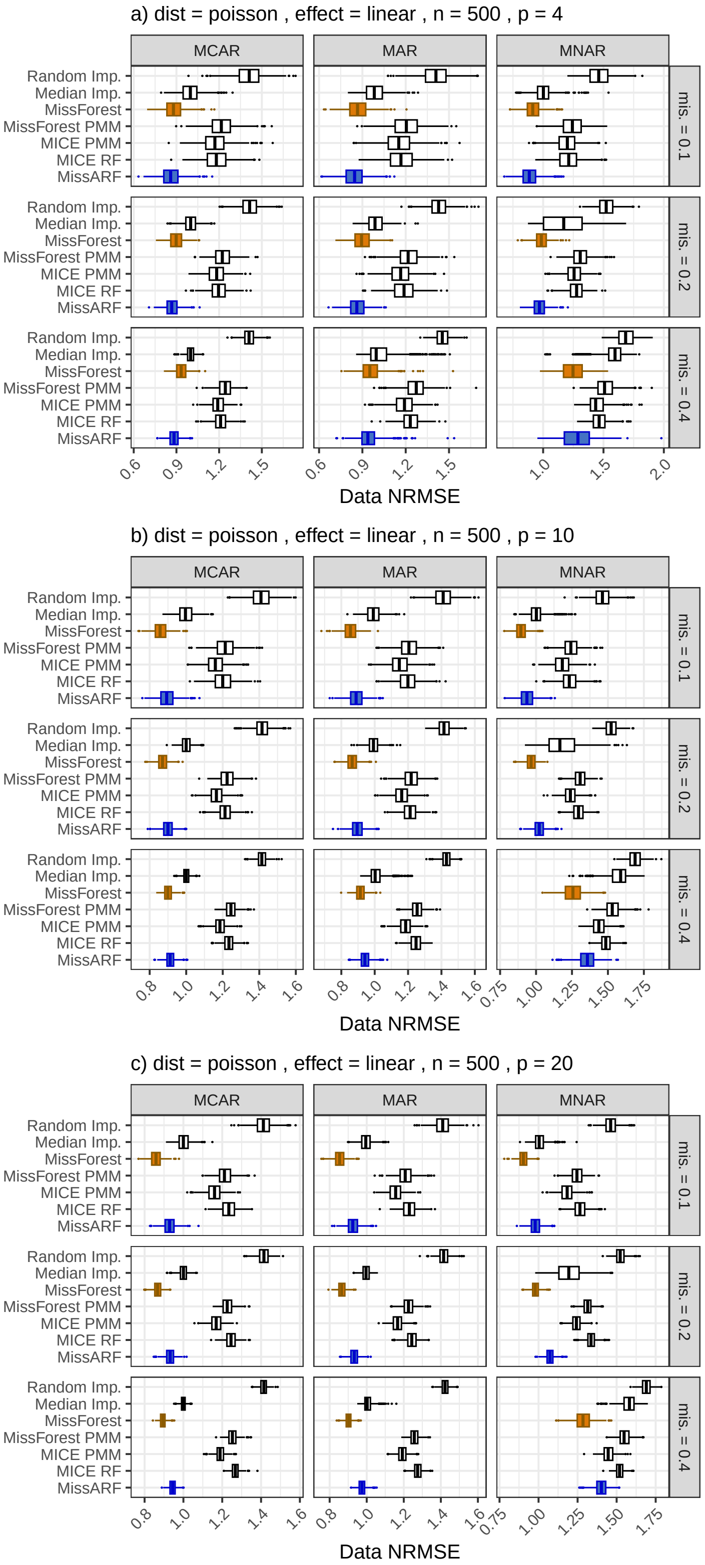

Figure S7: **NRMSE** of the Poisson distribution setting with a linear effect over different missingness patterns, dimensionality (p) and missingness rates (mis.) with $n = 500$. The boxplots are plotted over the replicates, with MissARF (blue) and MissForest (orange) highlighted.

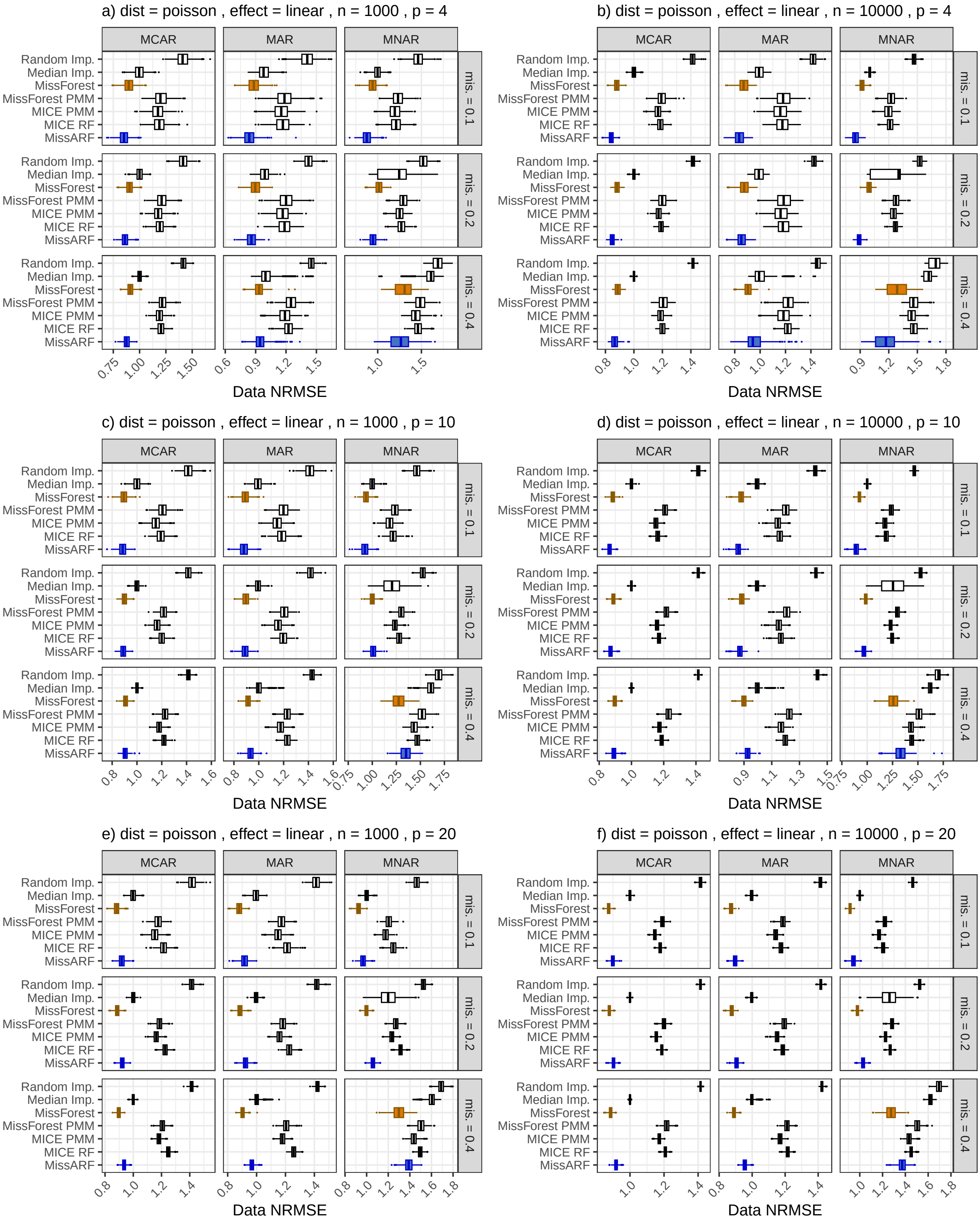

Figure S8: **NRMSE** of the Poisson distribution setting with a linear effect over different missingness patterns, dimensionality (p) and missingness rates (mis.) with $n = 1000$ (left) and $n = 10,000$ (right). The boxplots are plotted over the replicates, with MissARF (blue) and MissForest (orange) highlighted.

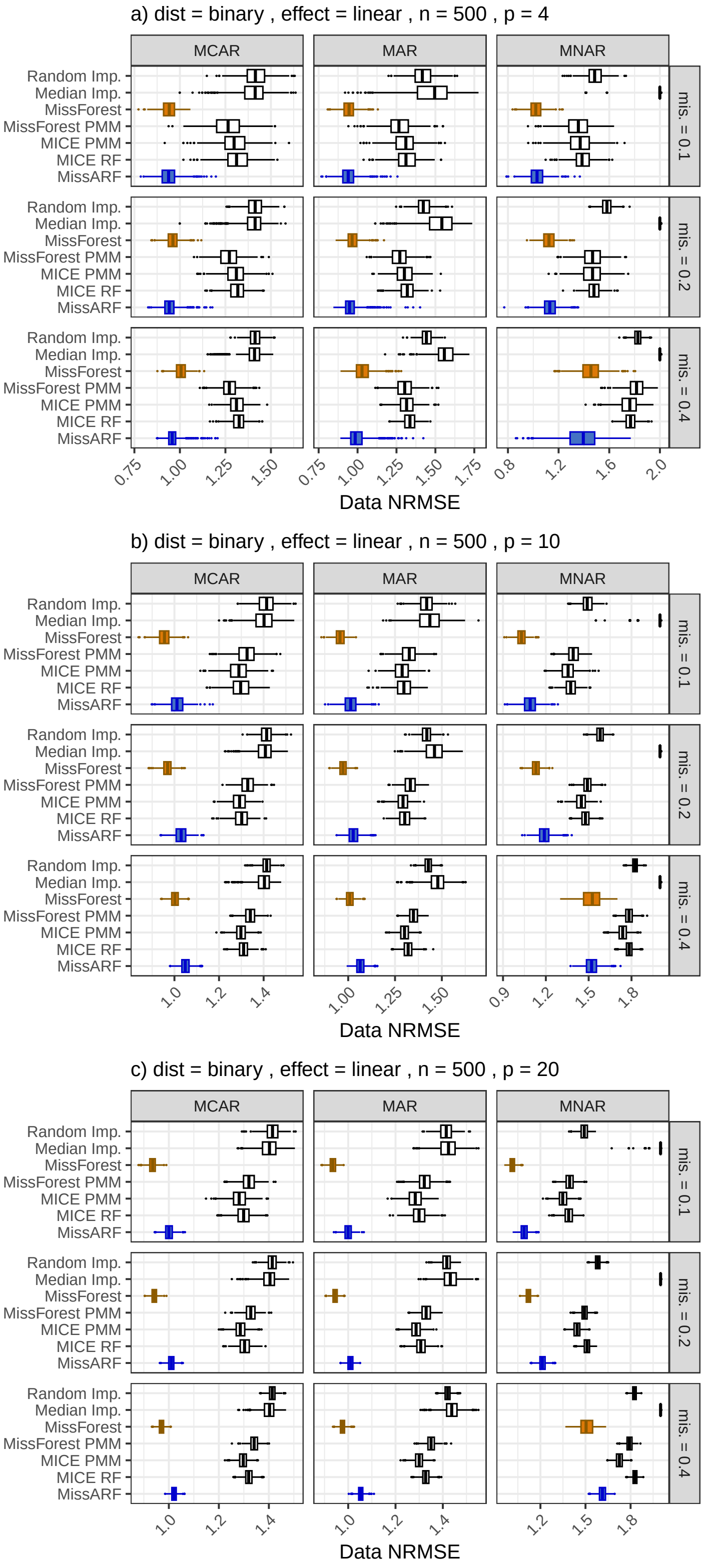

Figure S9: **NRMSE** of the binary distribution setting with a linear effect over different missingness patterns, dimensionality (p) and missingness rates (mis.) with $n = 500$. The boxplots are plotted over the replicates, with MissARF (blue) and MissForest (orange) highlighted.

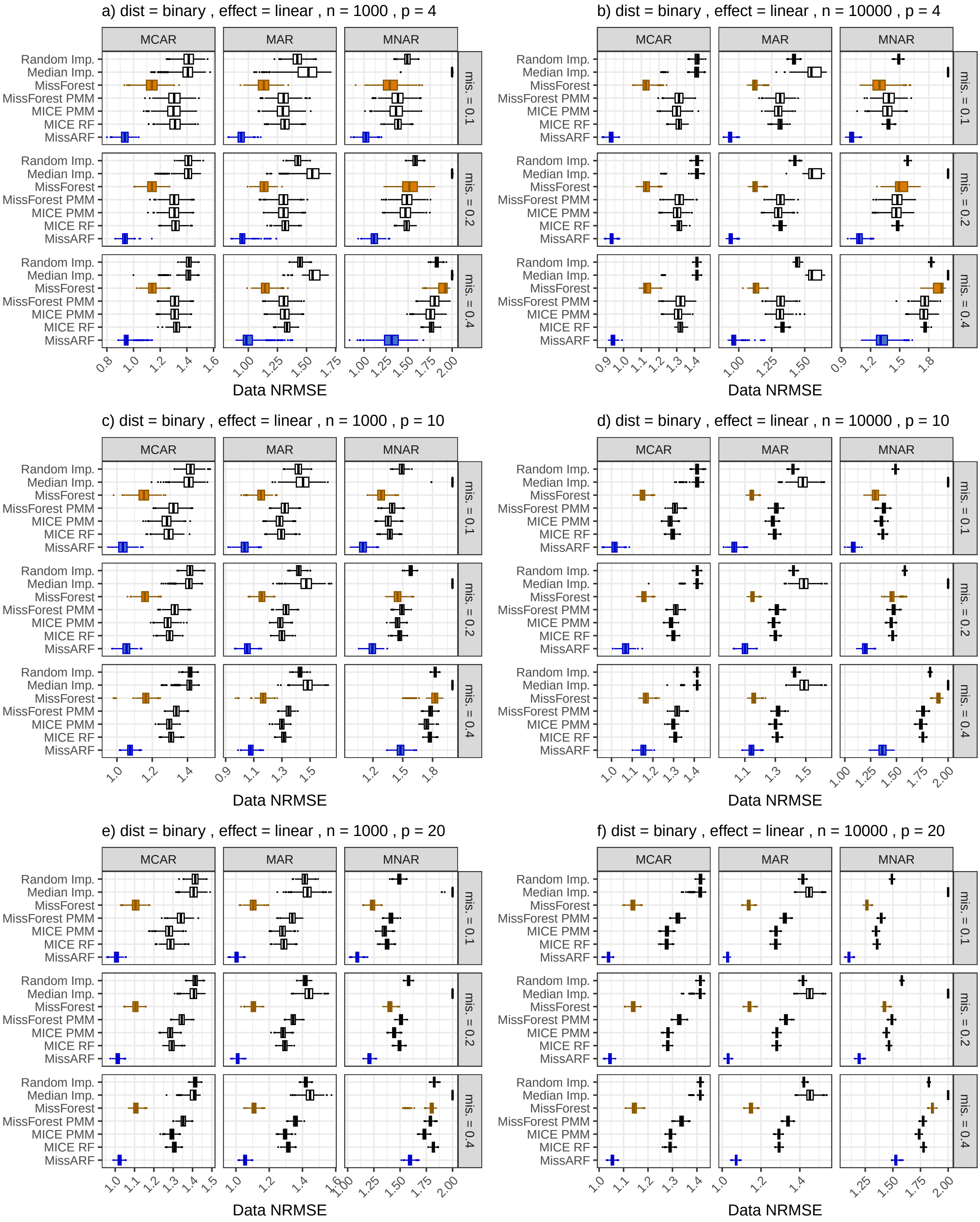

Figure S10: **NRMSE** of the binary distribution setting with a linear effect over different missingness patterns, dimensionality (p) and missingness rates (mis.) with $n = 1000$ (left) and $n = 10,000$ (right). The boxplots are plotted over the replicates, with MissARF (blue) and MissForest (orange) highlighted.

1.1.2 Squared effect

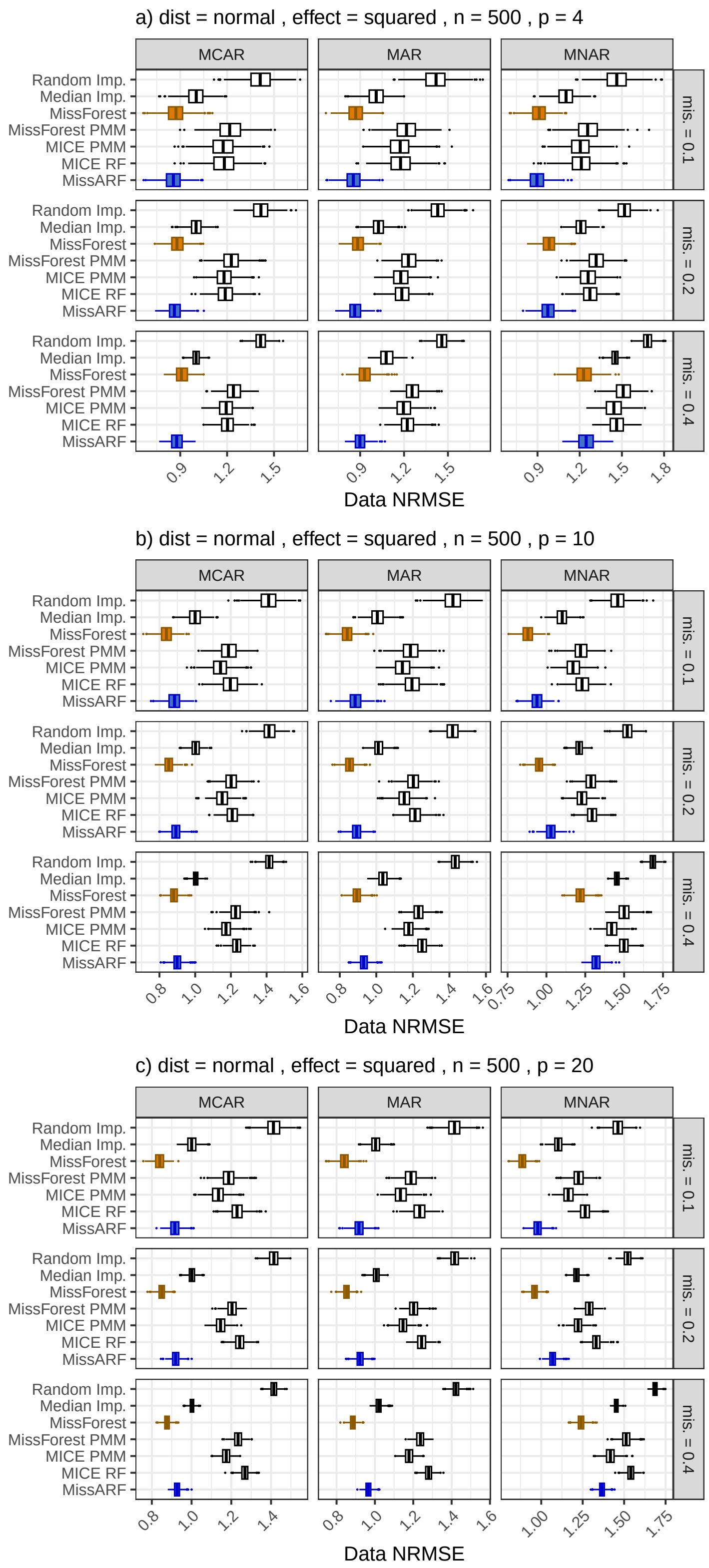

Figure S11: **NRMSE** of the normal distribution setting with a squared effect over different missingness patterns, dimensionality (p) and missingness rates (mis.) with $n = 500$. The boxplots are plotted over the replicates, with MissARF (blue) and MissForest (orange) highlighted.

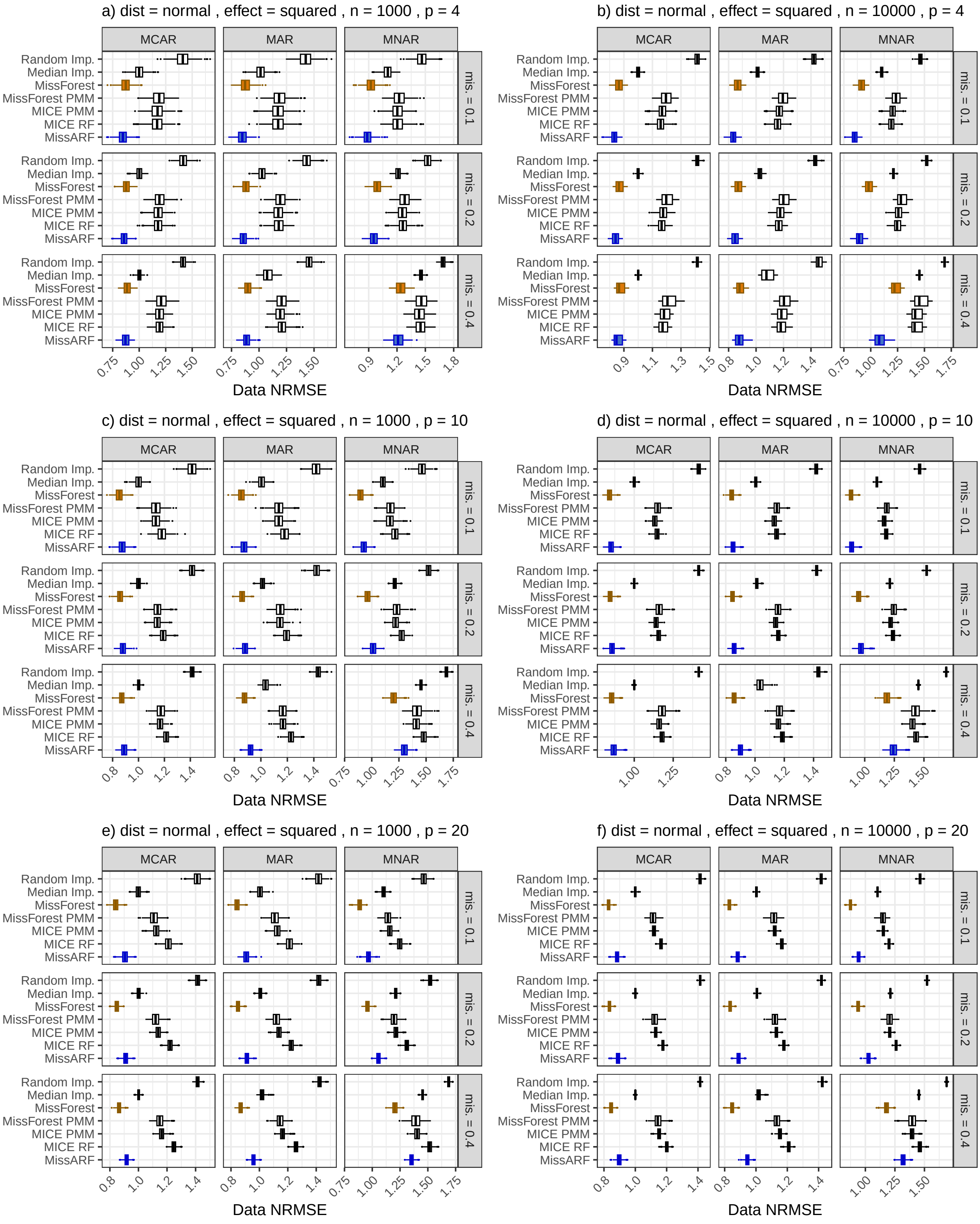

Figure S12: **NRMSE** of the normal distribution setting with a squared effect over different missingness patterns, dimensionality (p) and missingness rates (mis.) with $n = 1000$ (left) and $n = 10,000$ (right). The boxplots are plotted over the replicates, with MissARF (blue) and MissForest (orange) highlighted.

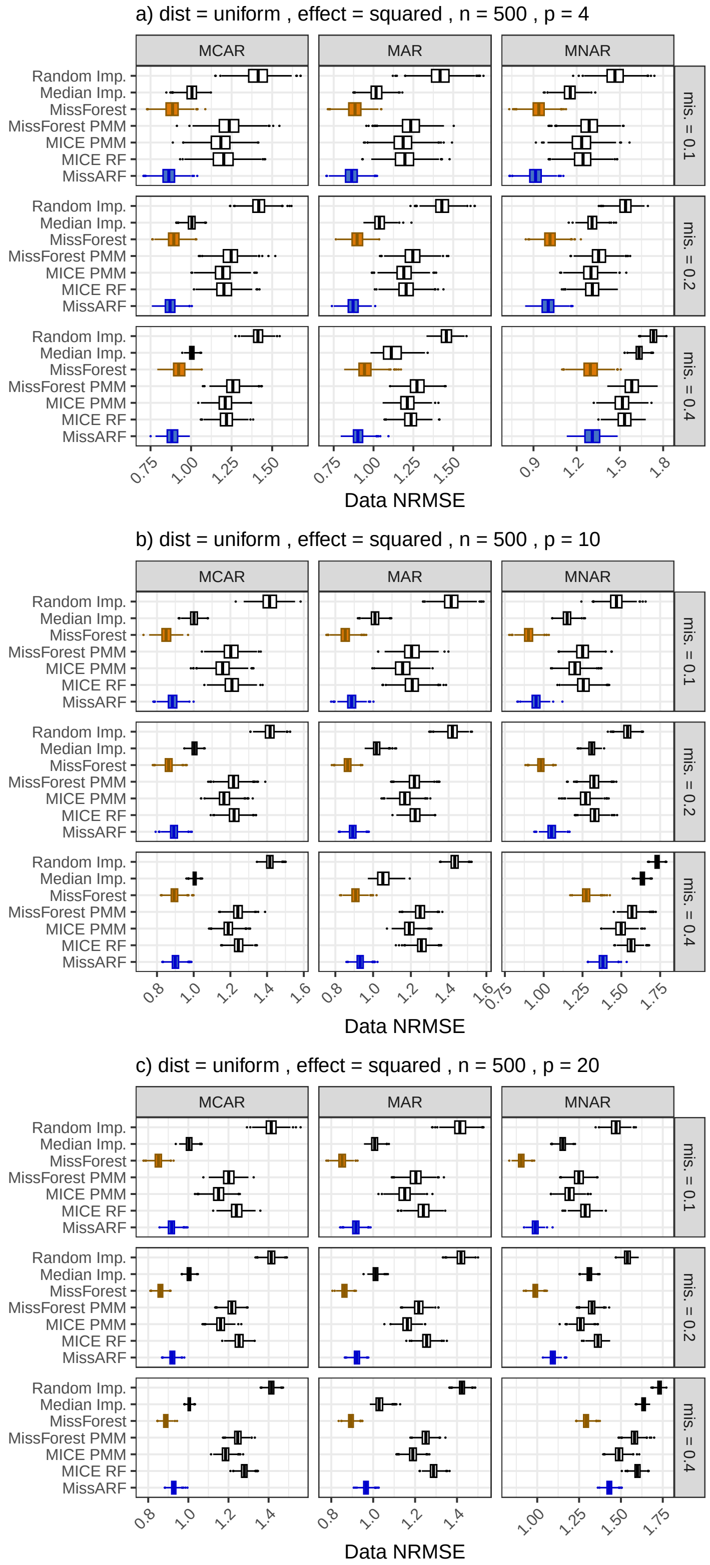

Figure S13: **NRMSE** of the uniform distribution setting with a squared effect over different missingness patterns, dimensionality (p) and missingness rates (mis.) with $n = 500$. The boxplots are plotted over the replicates, with MissARF (blue) and MissForest (orange) highlighted.

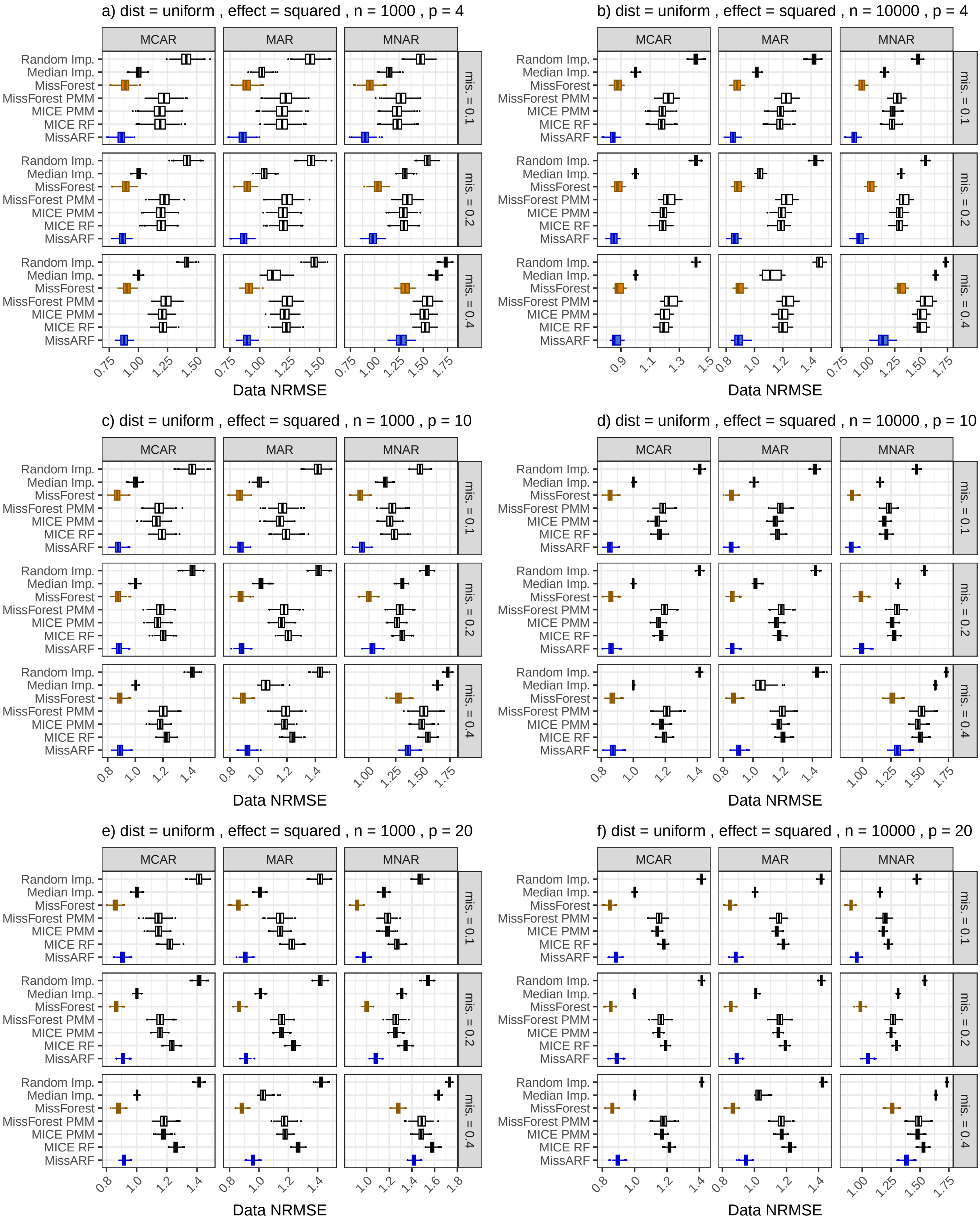

Figure S14: **NRMSE** of the uniform distribution setting with a squared effect over different missingness patterns, dimensionality (p) and missingness rates (mis.) with $n = 1000$ (left) and $n = 10,000$ (right). The boxplots are plotted over the replicates, with MissARF (blue) and MissForest (orange) highlighted.

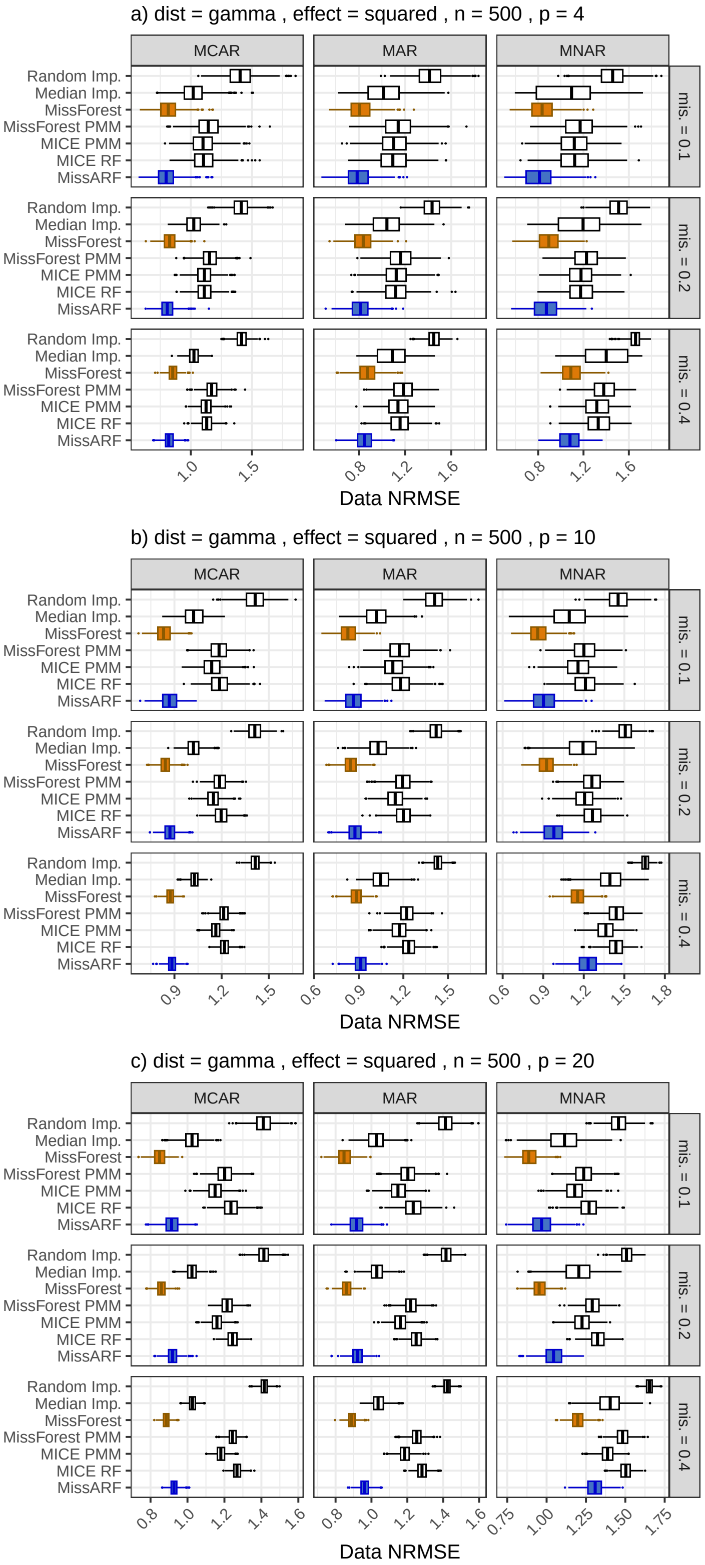

Figure S15: **NRMSE** of the gamma distribution setting with a squared effect over different missingness patterns, dimensionality (p) and missingness rates (mis.) with $n = 500$. The boxplots are plotted over the replicates, with MissARF (blue) and MissForest (orange) highlighted.

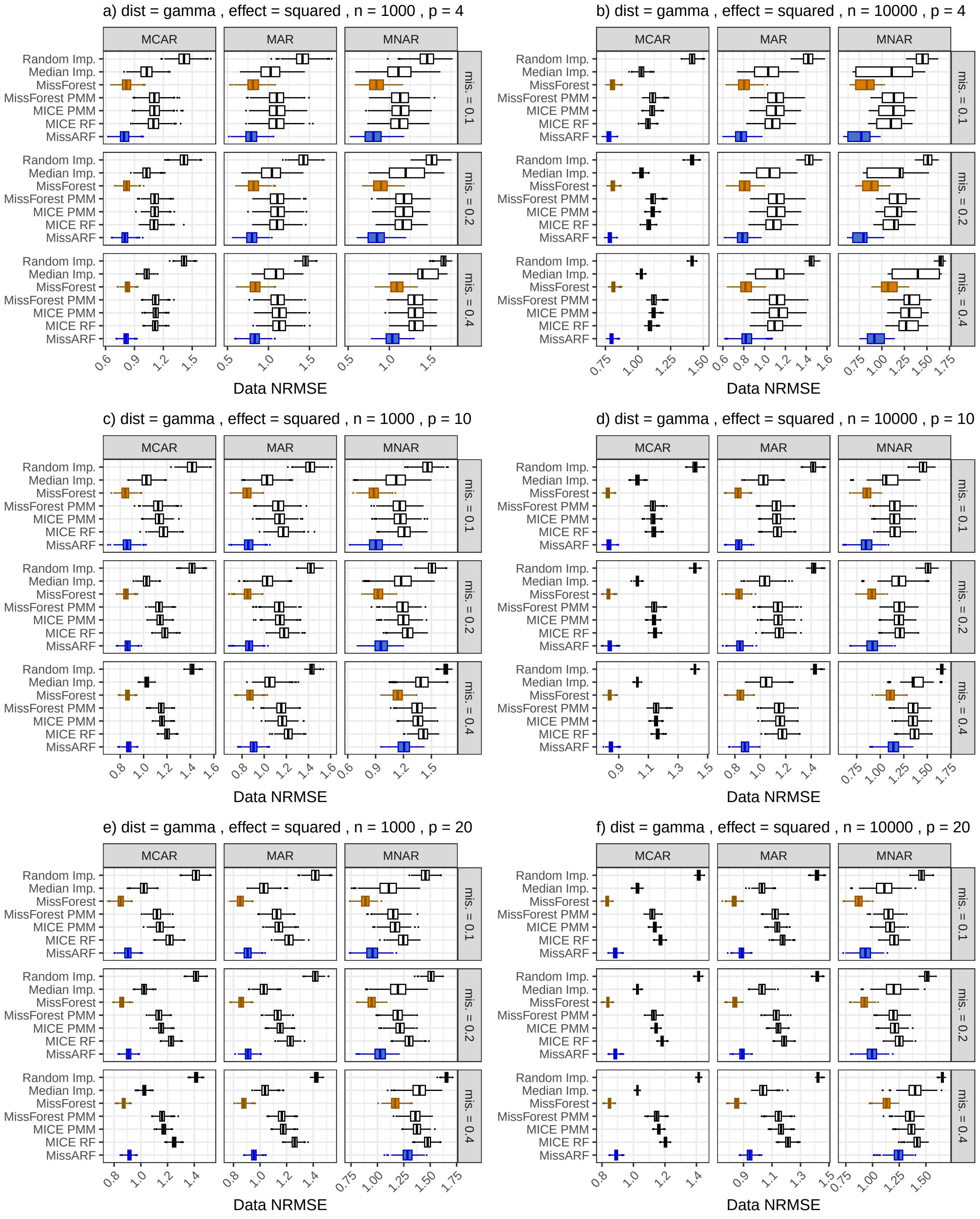

Figure S16: **NRMSE** of the gamma distribution setting with a squared effect over different missingness patterns, dimensionality (p) and missingness rates (mis.) with $n = 1000$ (left) and $n = 10,000$ (right). The boxplots are plotted over the replicates, with MissARF (blue) and MissForest (orange) highlighted.

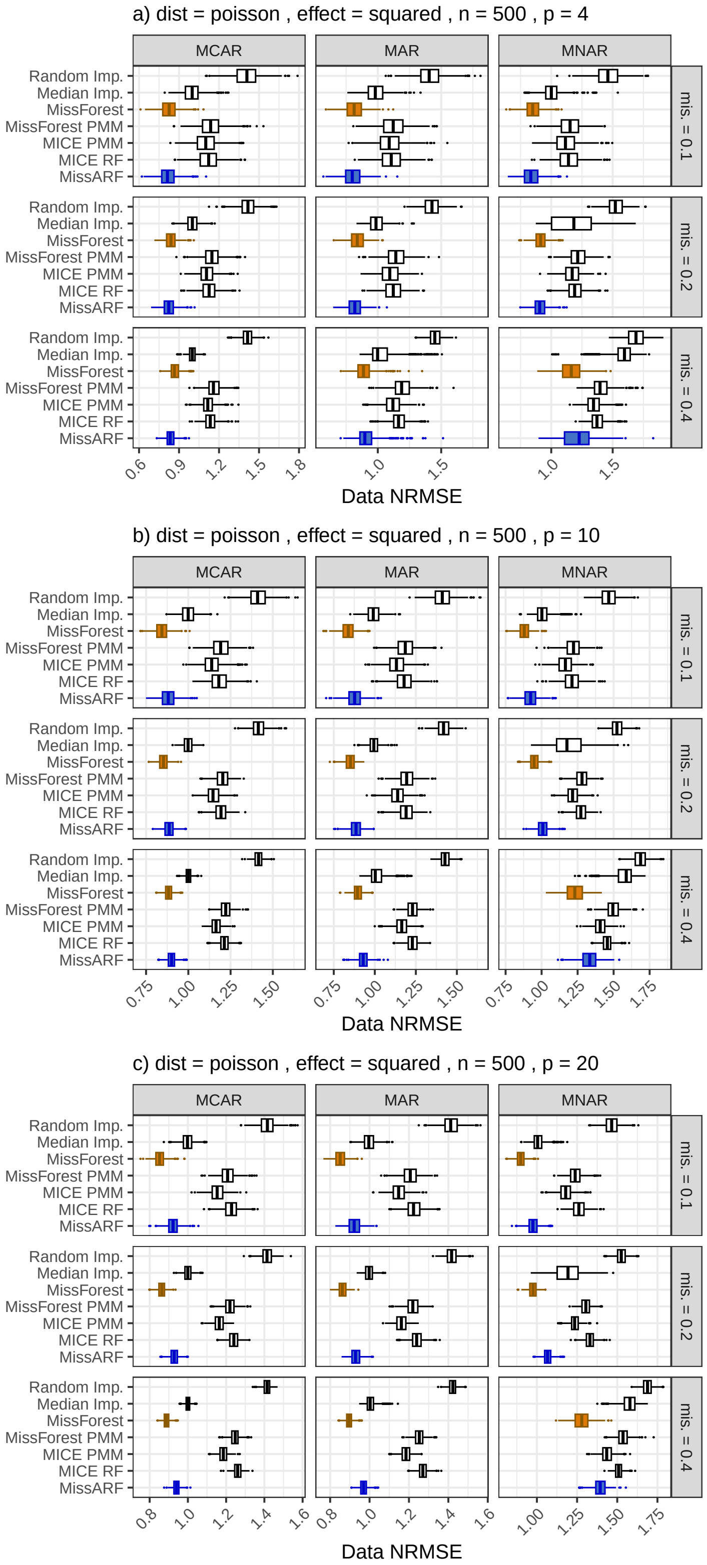

Figure S17: **NRMSE** of the Poisson distribution setting with a squared effect over different missingness patterns, dimensionality (p) and missingness rates (mis.) with $n = 500$. The boxplots are plotted over the replicates, with MissARF (blue) and MissForest (orange) highlighted.

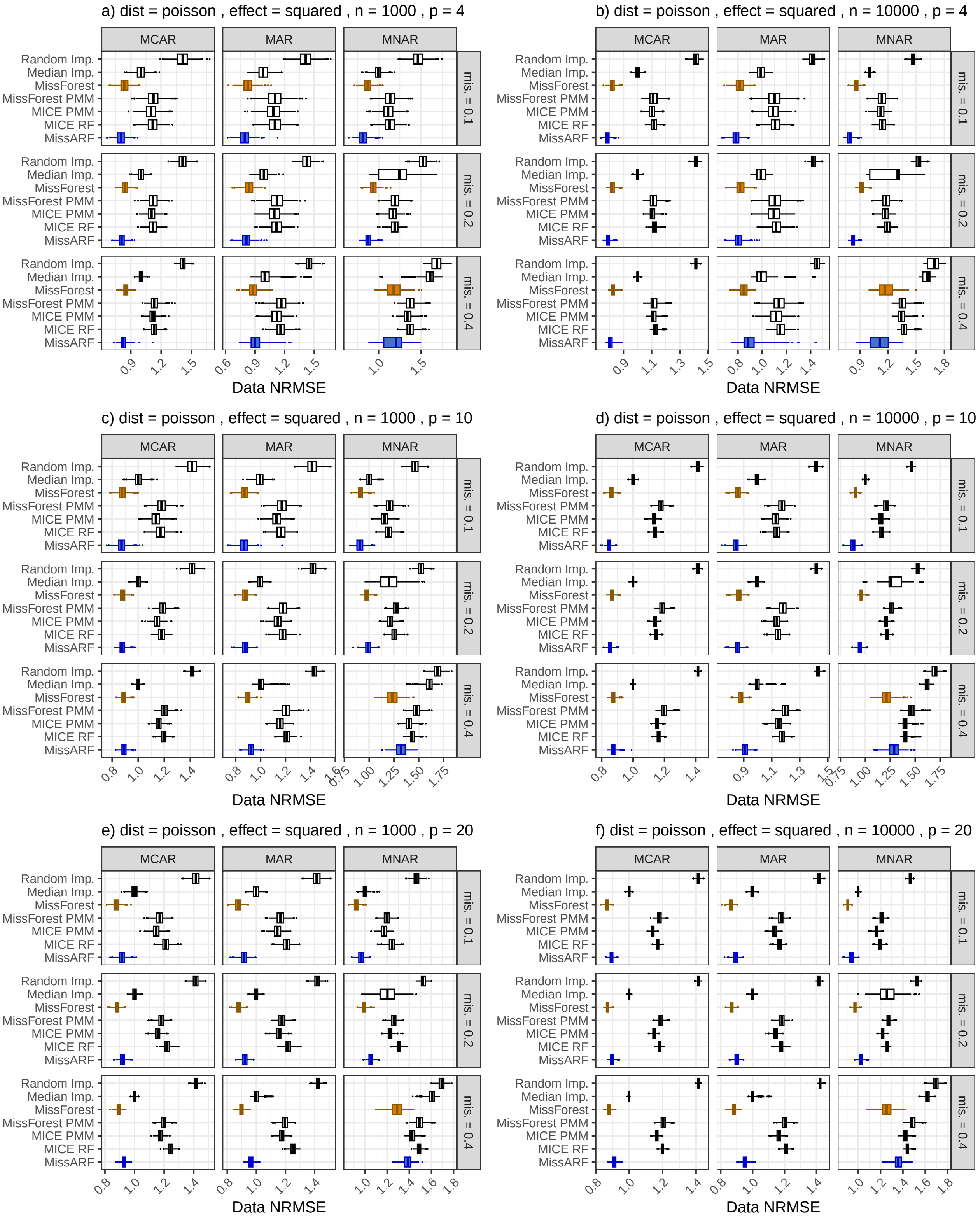

Figure S18: **NRMSE** of the Poisson distribution setting with a squared effect over different missingness patterns, dimensionality (p) and missingness rates (mis.) with $n = 1000$ (left) and $n = 10,000$ (right). The boxplots are plotted over the replicates, with MissARF (blue) and MissForest (orange) highlighted.

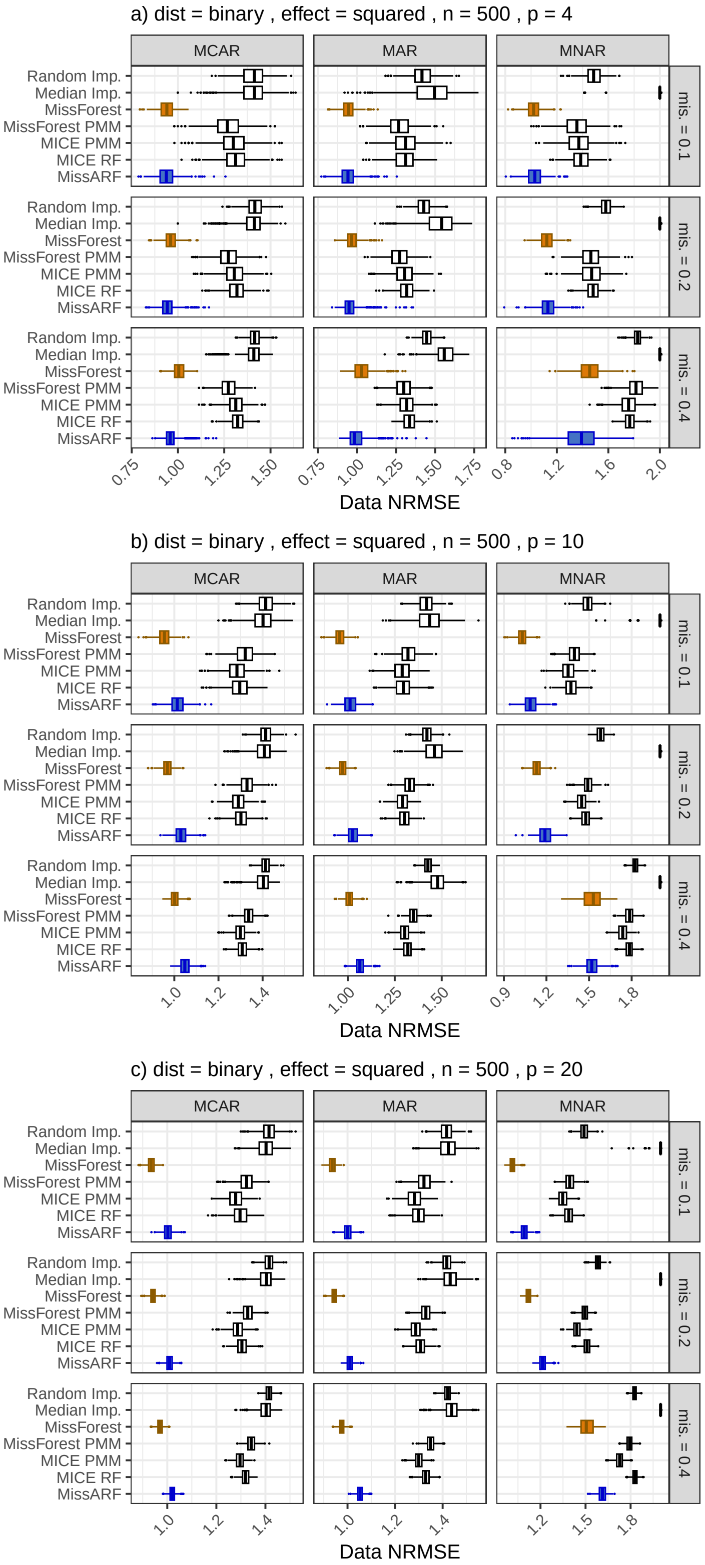

Figure S19: **NRMSE** of the binary distribution setting with a squared effect over different missingness patterns, dimensionality (p) and missingness rates (mis.) with $n = 500$. The boxplots are plotted over the replicates, with MissARF (blue) and MissForest (orange) highlighted.

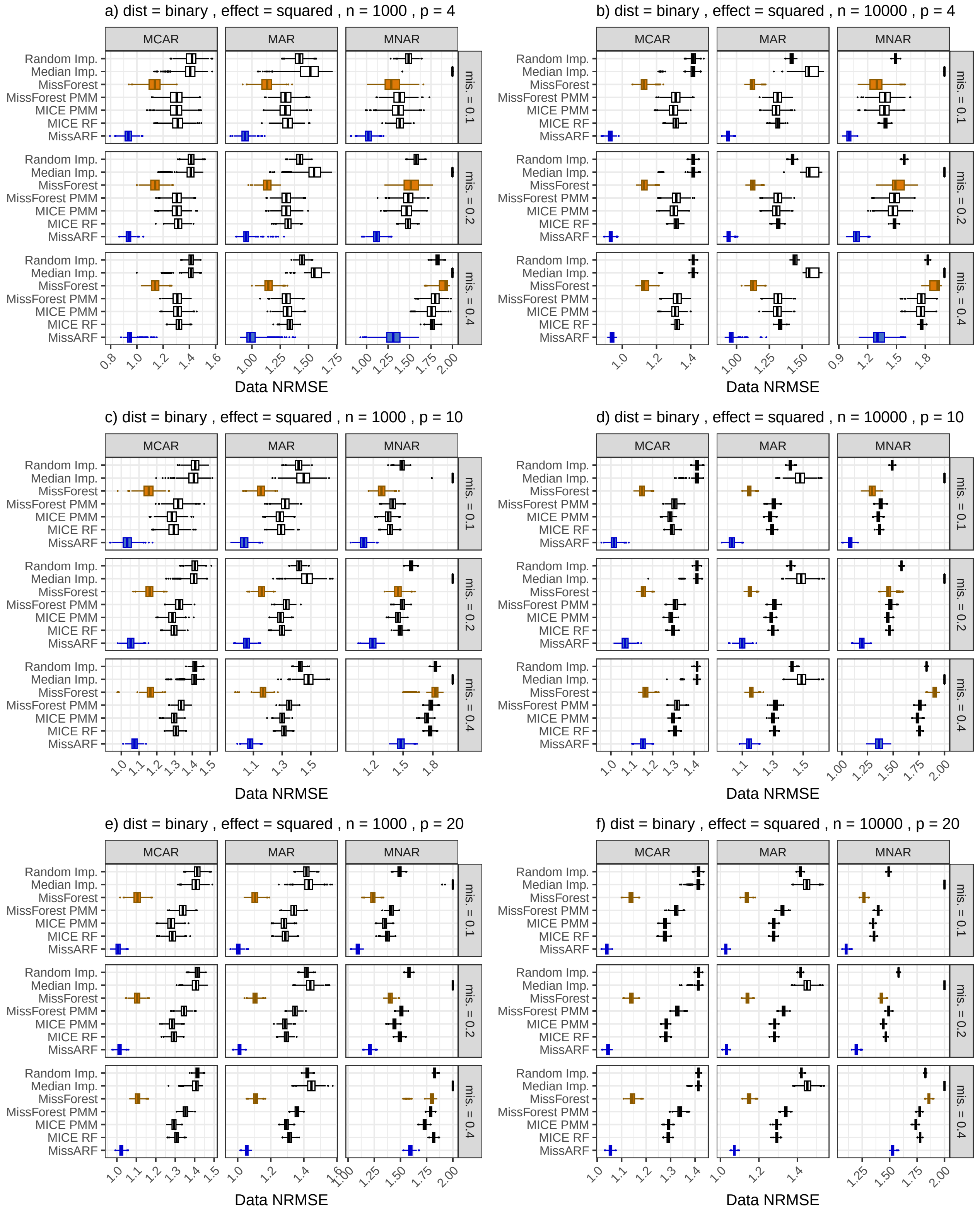

Figure S20: **NRMSE** of the binary distribution setting with a squared effect over different missingness patterns, dimensionality (p) and missingness rates (mis.) with $n = 1000$ (left) and $n = 10,000$ (right). The boxplots are plotted over the replicates, with MissARF (blue) and MissForest (orange) highlighted.

1.2 Brier Score

1.2.1 Linear effect

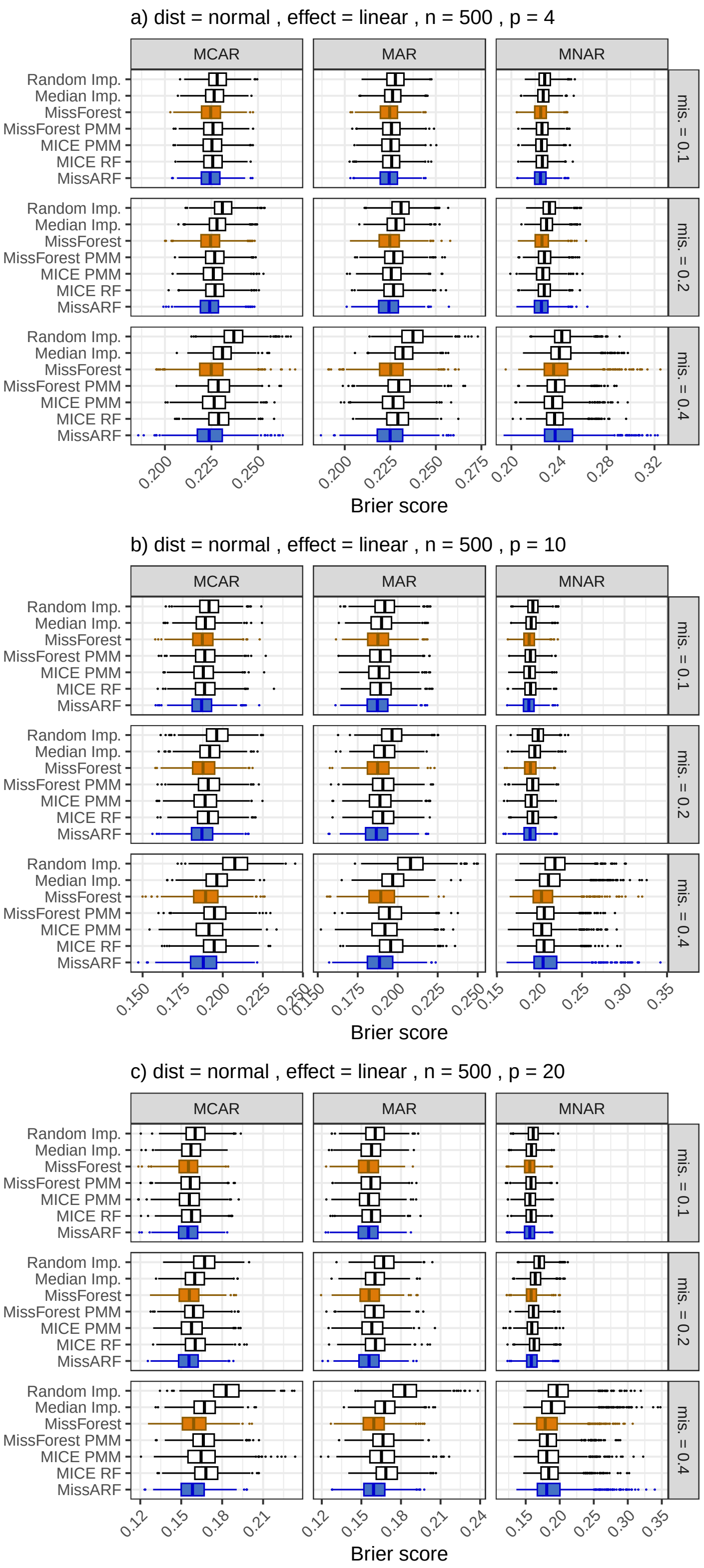

Figure S21: **Brier Score** of the normal distribution setting with a linear effect over different missingness patterns, dimensionality (p) and missingness rates (mis.) with $n = 500$. The boxplots are plotted over the replicates, with MissARF (blue) and MissForest (orange) highlighted.

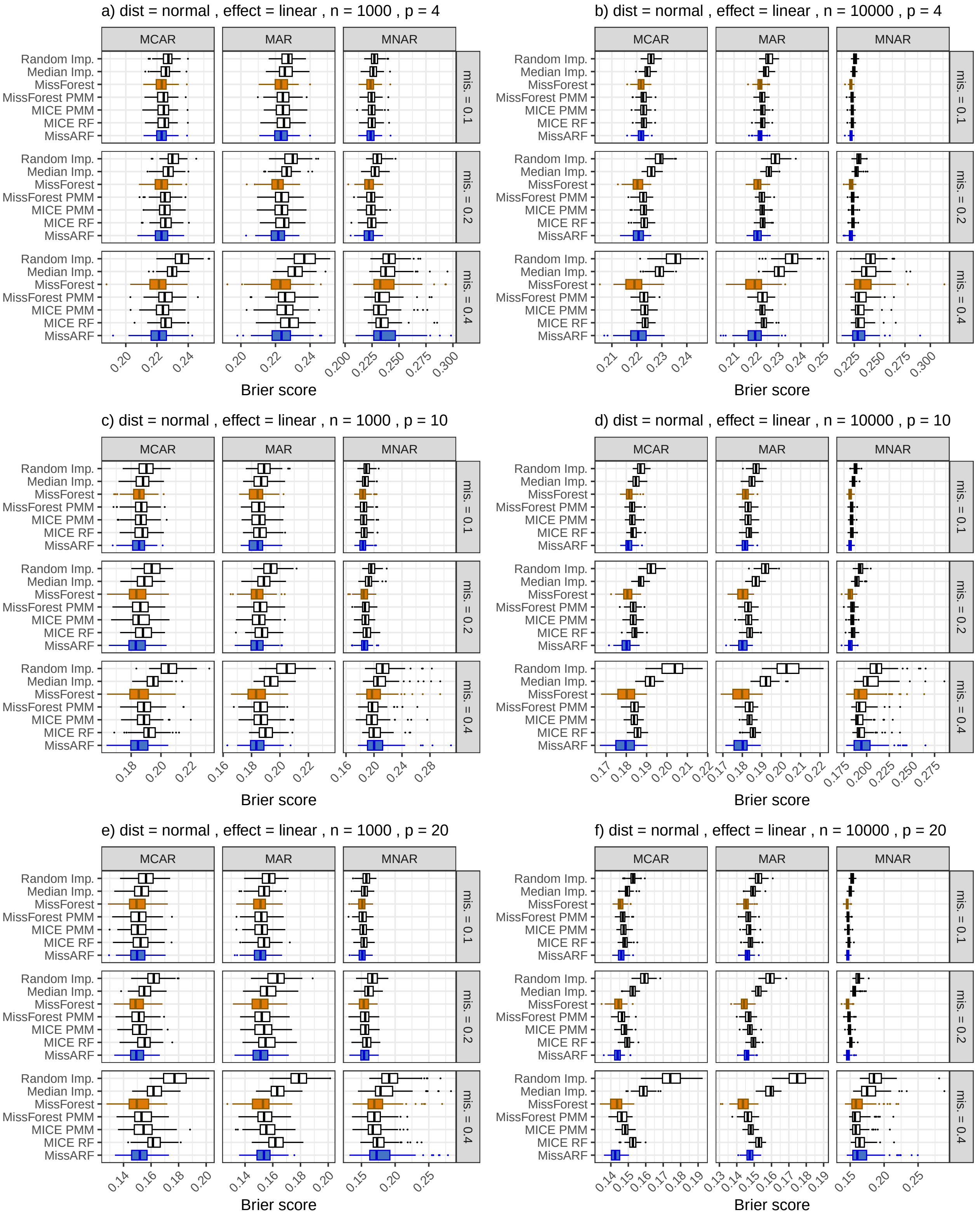

Figure S22: **Brier Score** of the normal distribution setting with a linear effect over different missingness patterns, dimensionality (p) and missingness rates (mis.) with $n = 1000$ (left) and $n = 10,000$ (right). The boxplots are plotted over the replicates, with MissARF (blue) and MissForest (orange) highlighted.

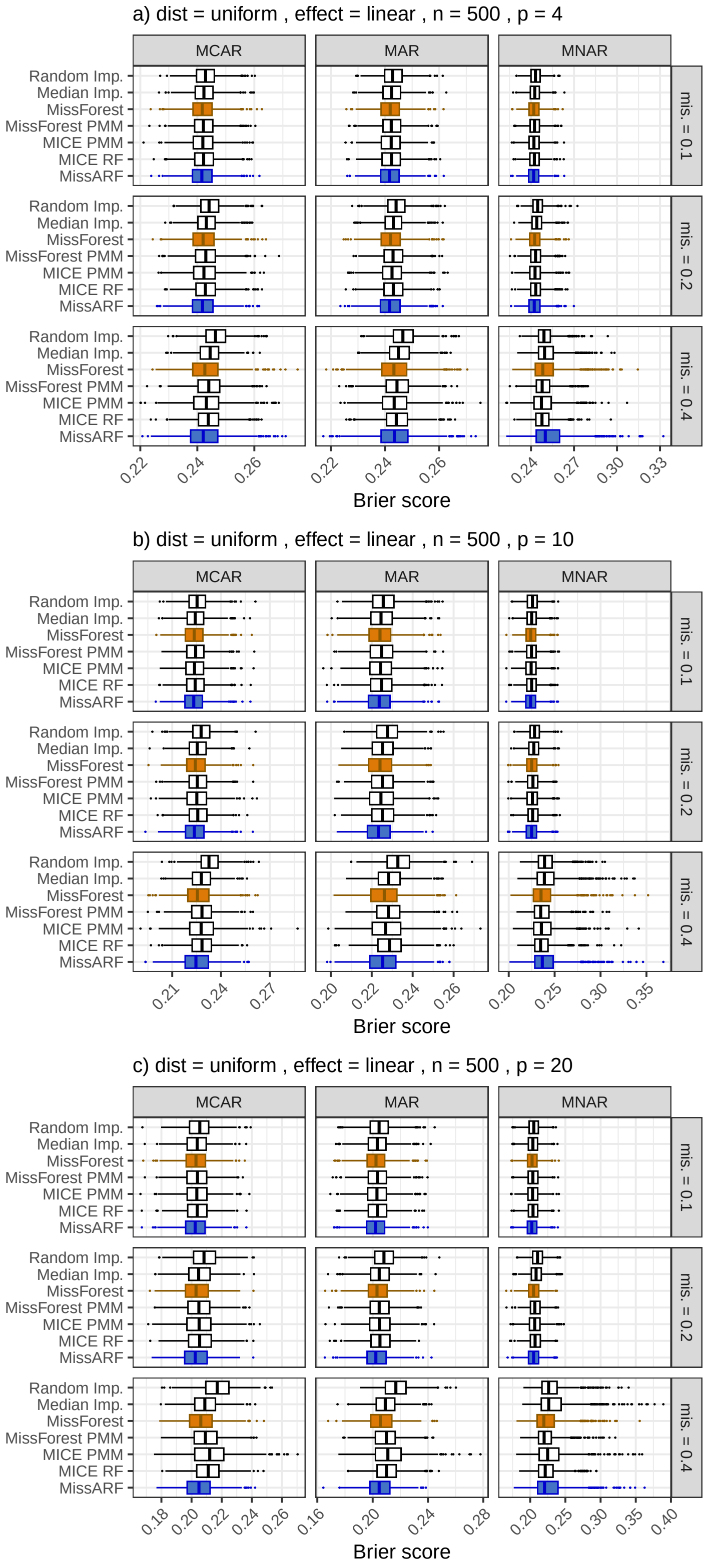

Figure S23: **Brier Score** of the uniform distribution setting with a linear effect over different missingness patterns, dimensionality (p) and missingness rates (mis.) with $n = 500$. The boxplots are plotted over the replicates, with MissARF (blue) and MissForest (orange) highlighted.

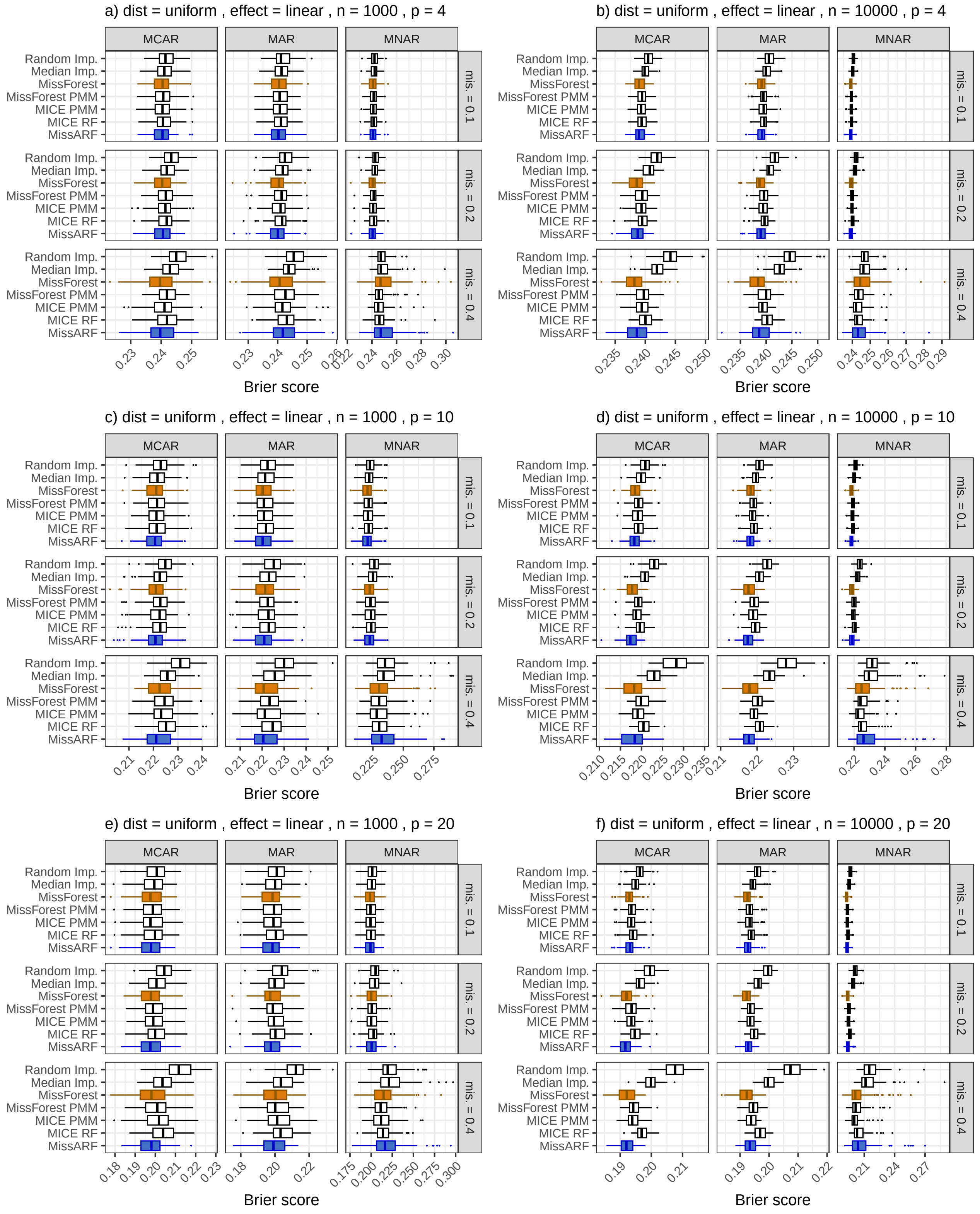

Figure S24: **Brier Score** of the uniform distribution setting with a linear effect over different missingness patterns, dimensionality (p) and missingness rates (mis.) with $n = 1000$ (left) and $n = 10,000$ (right). The boxplots are plotted over the replicates, with MissARF (blue) and MissForest (orange) highlighted.

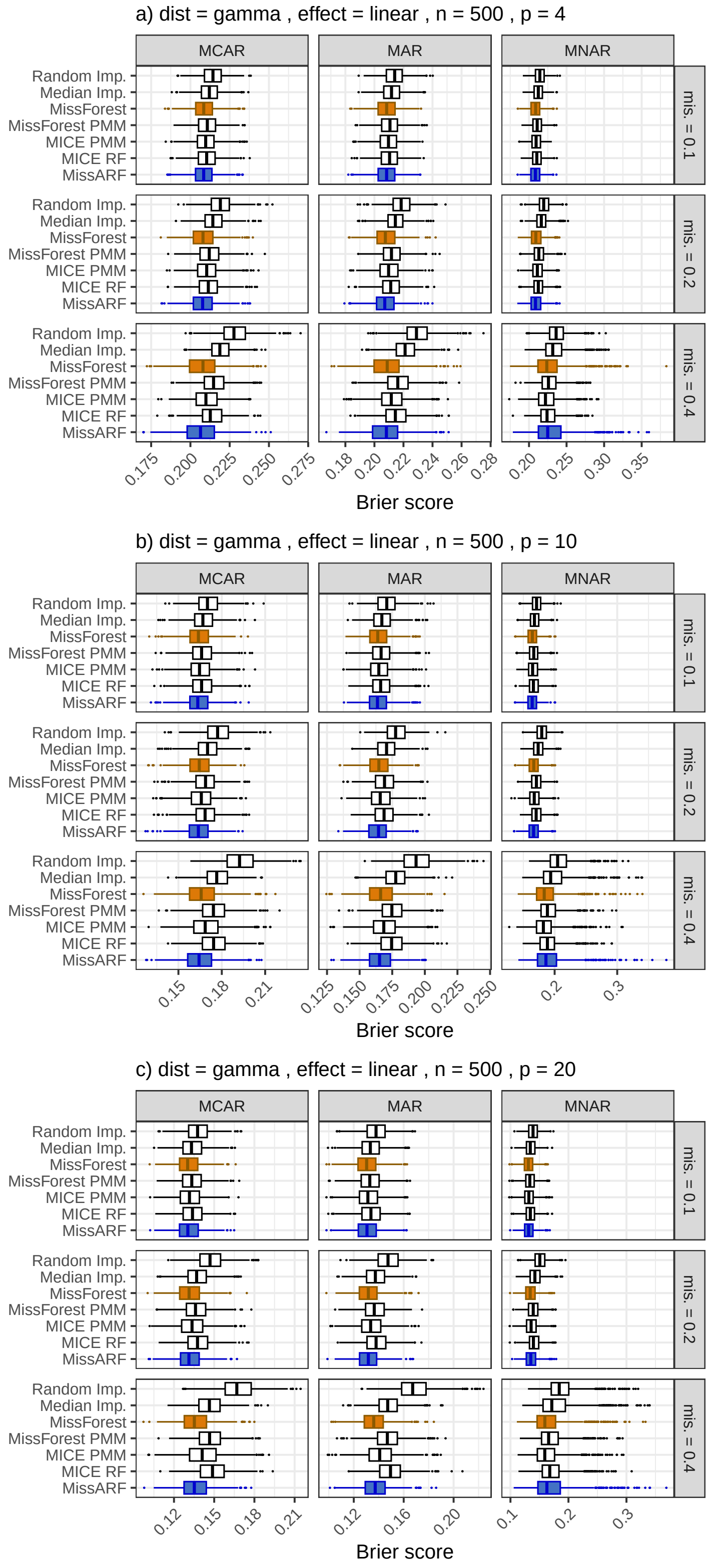

Figure S25: **Brier Score** of the gamma distribution setting with a linear effect over different missingness patterns, dimensionality (p) and missingness rates (mis.) with $n = 500$. The boxplots are plotted over the replicates, with MissARF (blue) and MissForest (orange) highlighted.

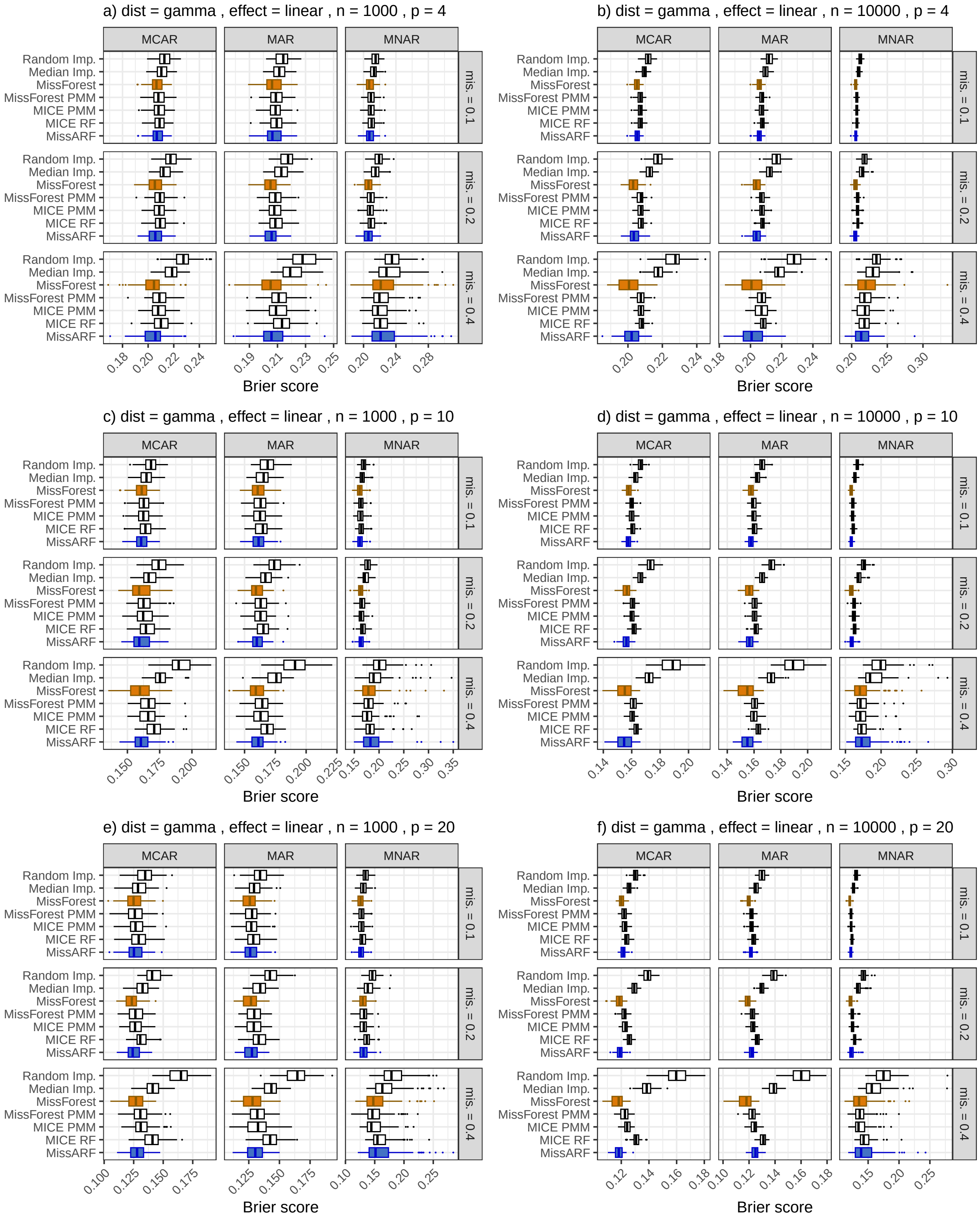

Figure S26: **Brier Score** of the gamma distribution setting with a linear effect over different missingness patterns, dimensionality (p) and missingness rates (mis.) with $n = 1000$ (left) and $n = 10,000$ (right). The boxplots are plotted over the replicates, with MissARF (blue) and MissForest (orange) highlighted.

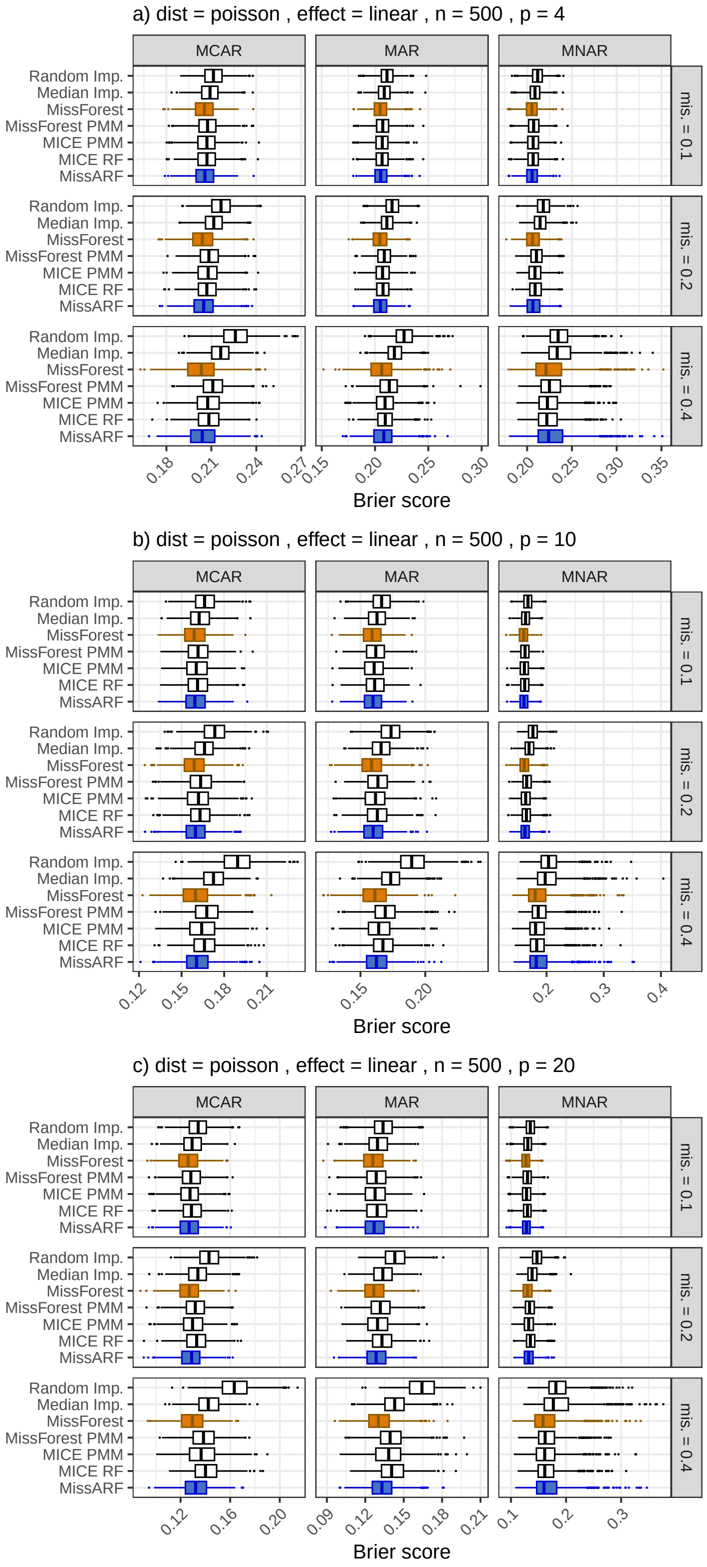

Figure S27: **Brier Score** of the Poisson distribution setting with a linear effect over different missingness patterns, dimensionality (p) and missingness rates (mis.) with $n = 500$. The boxplots are plotted over the replicates, with MissARF (blue) and MissForest (orange) highlighted.

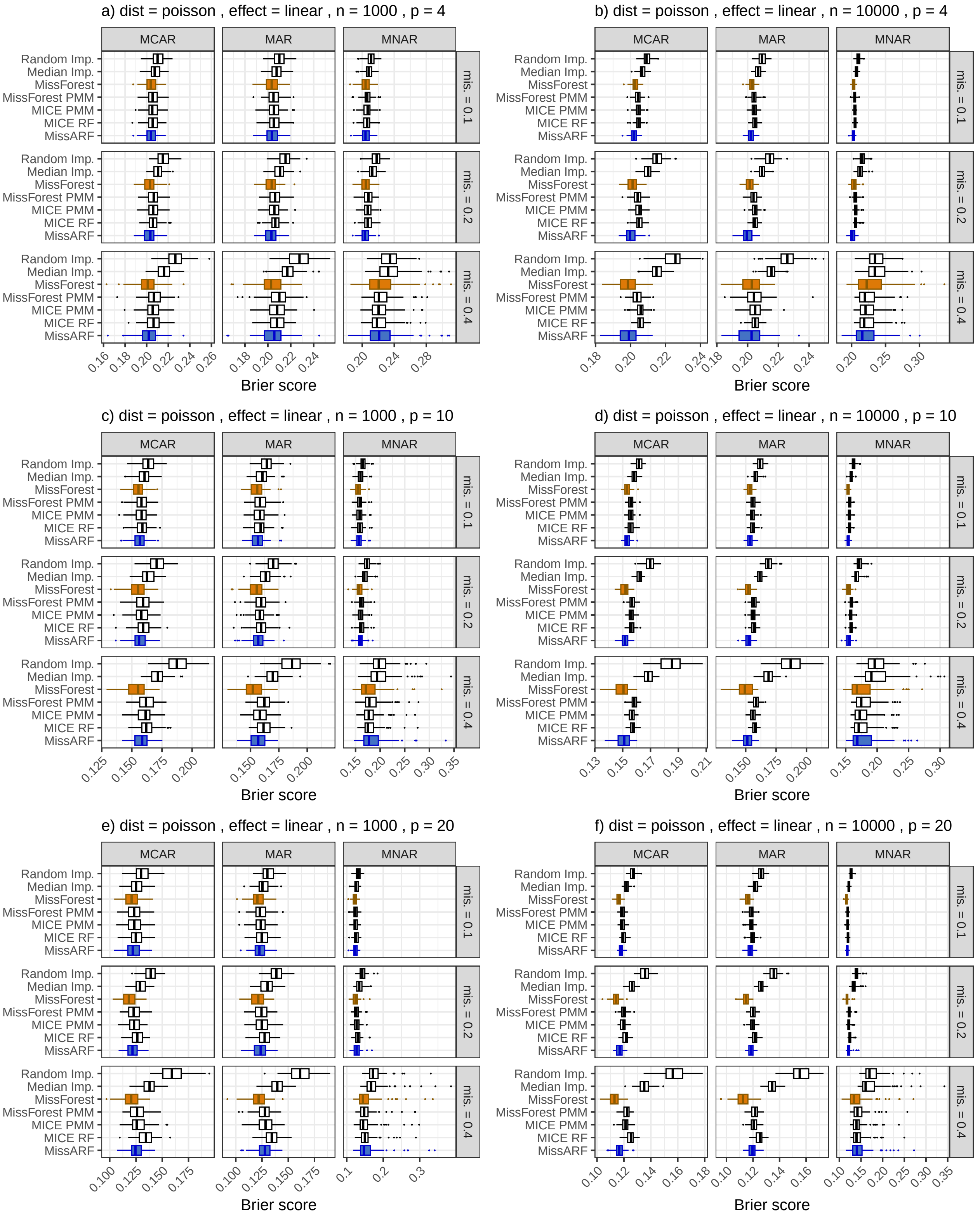

Figure S28: **Brier Score** of the Poisson distribution setting with a linear effect over different missingness patterns, dimensionality (p) and missingness rates (mis.) with $n = 1000$ (left) and $n = 10,000$ (right). The boxplots are plotted over the replicates, with MissARF (blue) and MissForest (orange) highlighted.

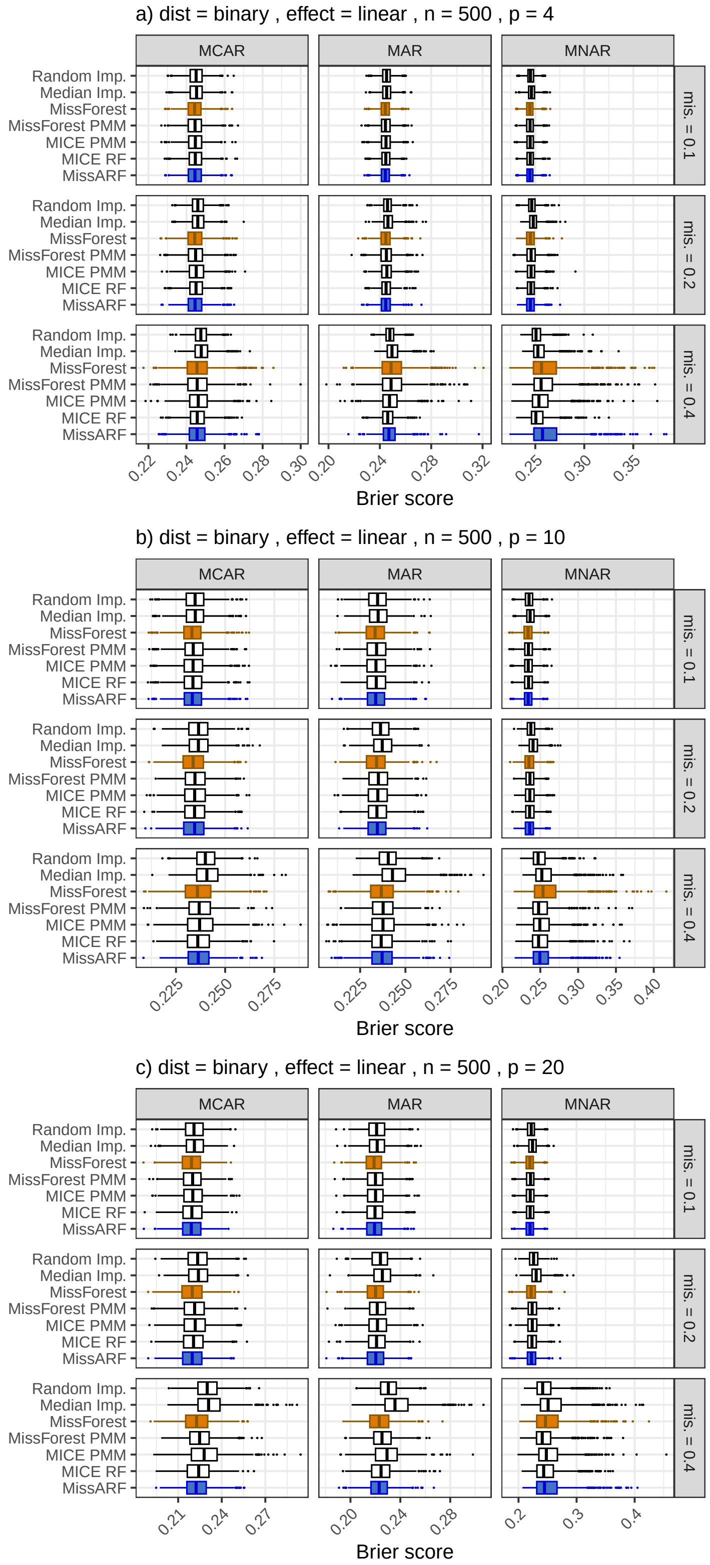

Figure S29: **Brier Score** of the binary distribution setting with a linear effect over different missingness patterns, dimensionality (p) and missingness rates (mis.) with $n = 500$. The boxplots are plotted over the replicates, with MissARF (blue) and MissForest (orange) highlighted.

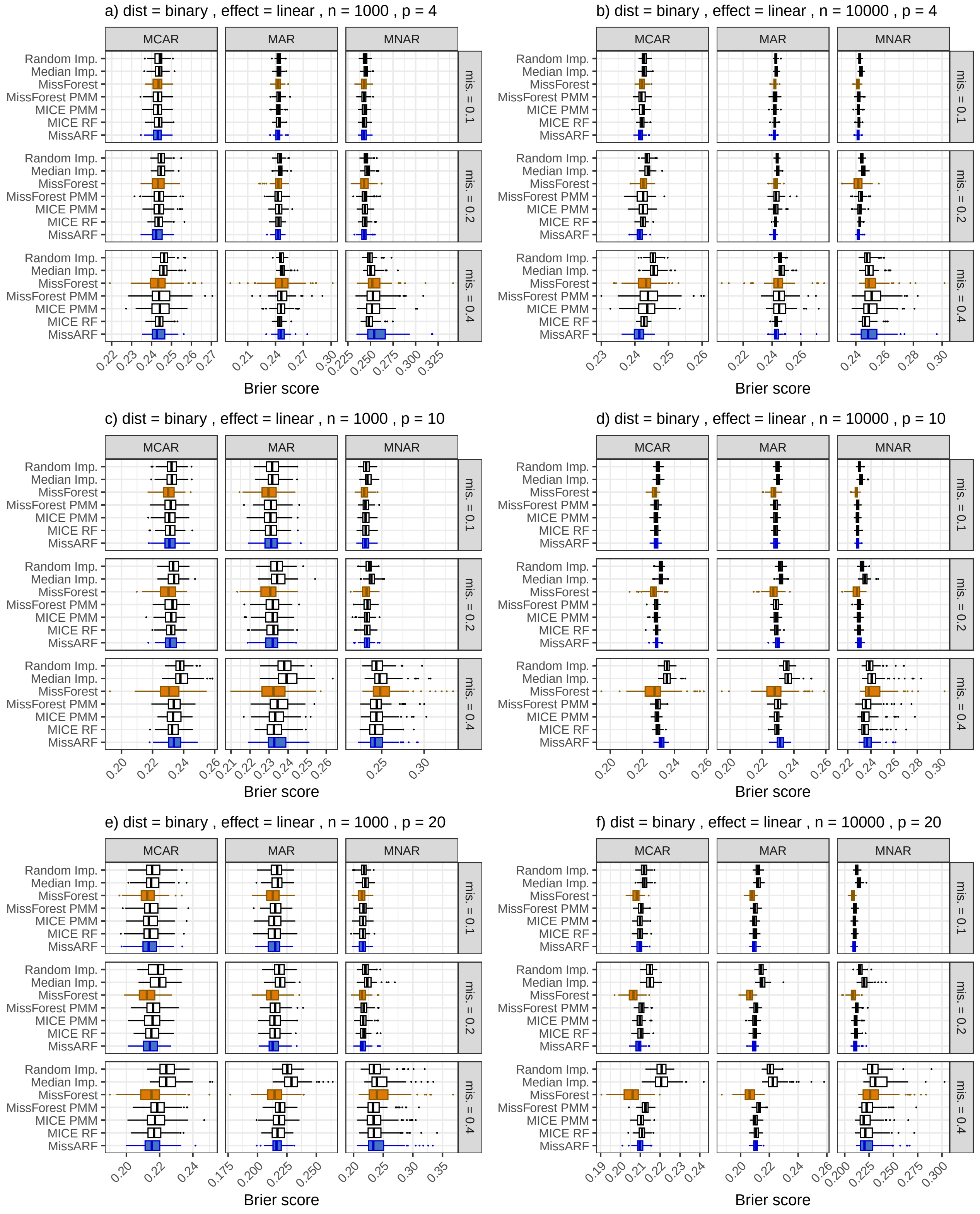

Figure S30: **Brier Score** of the binary distribution setting with a linear effect over different missingness patterns, dimensionality (p) and missingness rates (mis.) with $n = 1000$ (left) and $n = 10,000$ (right). The boxplots are plotted over the replicates, with MissARF (blue) and MissForest (orange) highlighted.

1.2.2 Squared effect

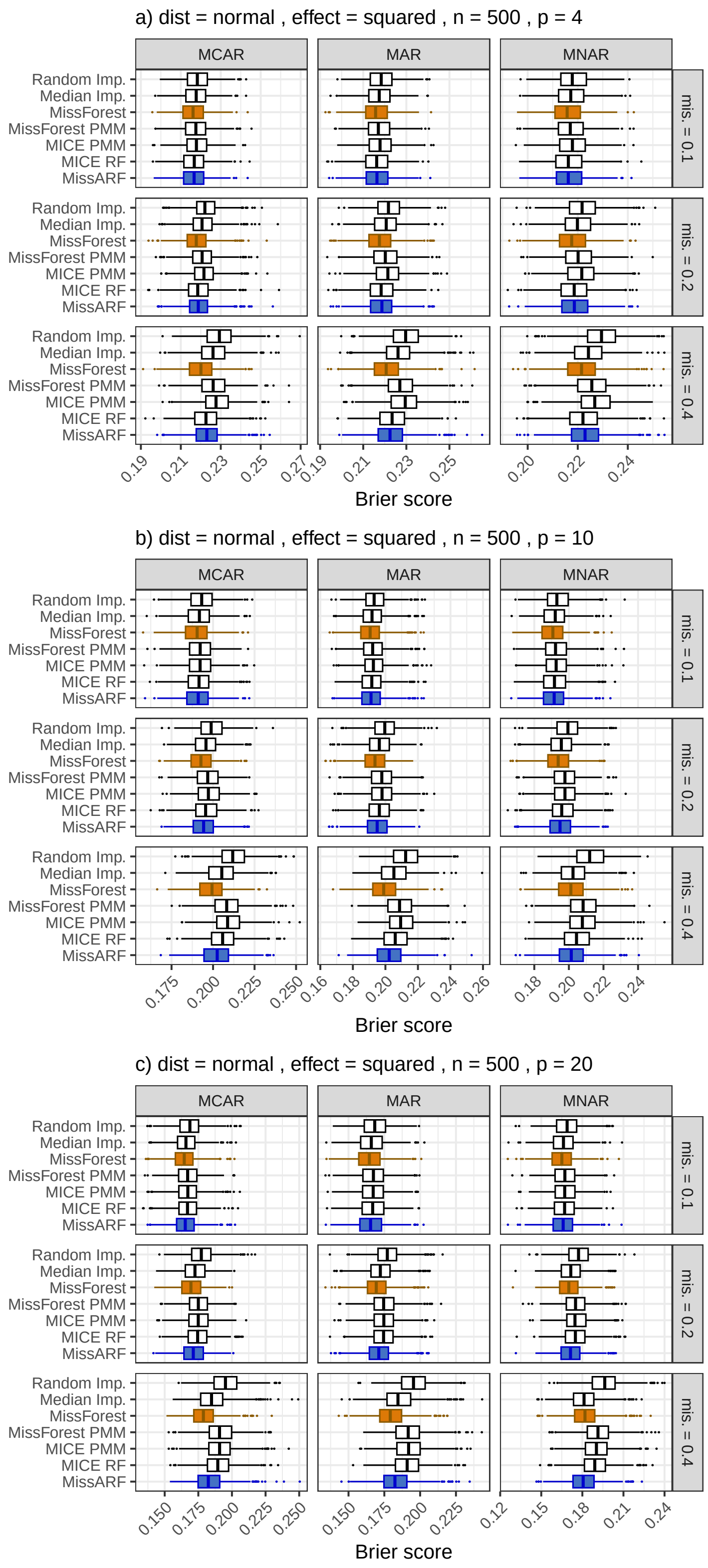

Figure S31: **Brier Score** of the normal distribution setting with a squared effect over different missingness patterns, dimensionality (p) and missingness rates (mis.) with $n = 500$. The boxplots are plotted over the replicates, with MissARF (blue) and MissForest (orange) highlighted.

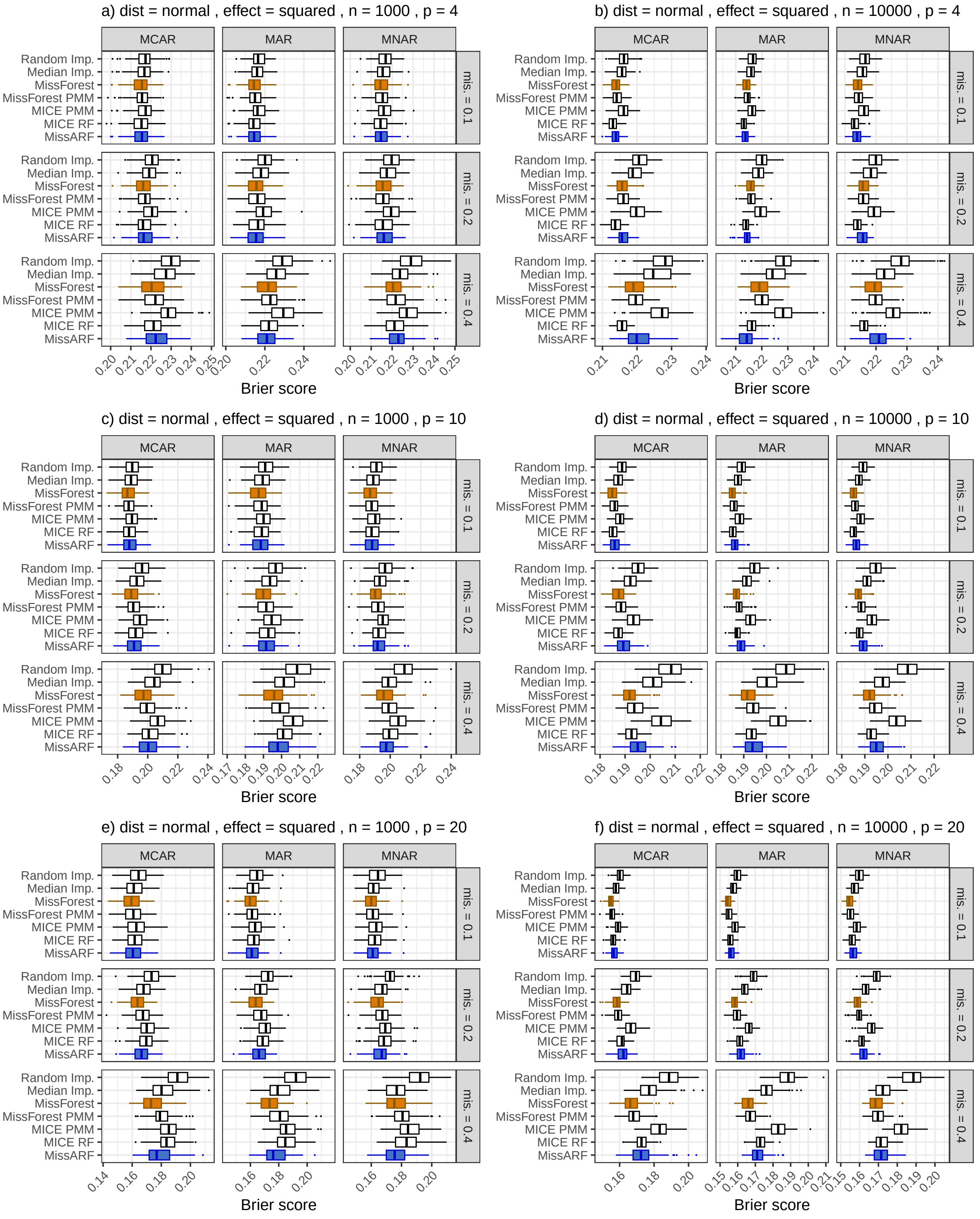

Figure S32: **Brier Score** of the normal distribution setting with a squared effect over different missingness patterns, dimensionality (p) and missingness rates (mis.) with $n = 1000$ (left) and $n = 10,000$ (right). The boxplots are plotted over the replicates, with MissARF (blue) and MissForest (orange) highlighted.

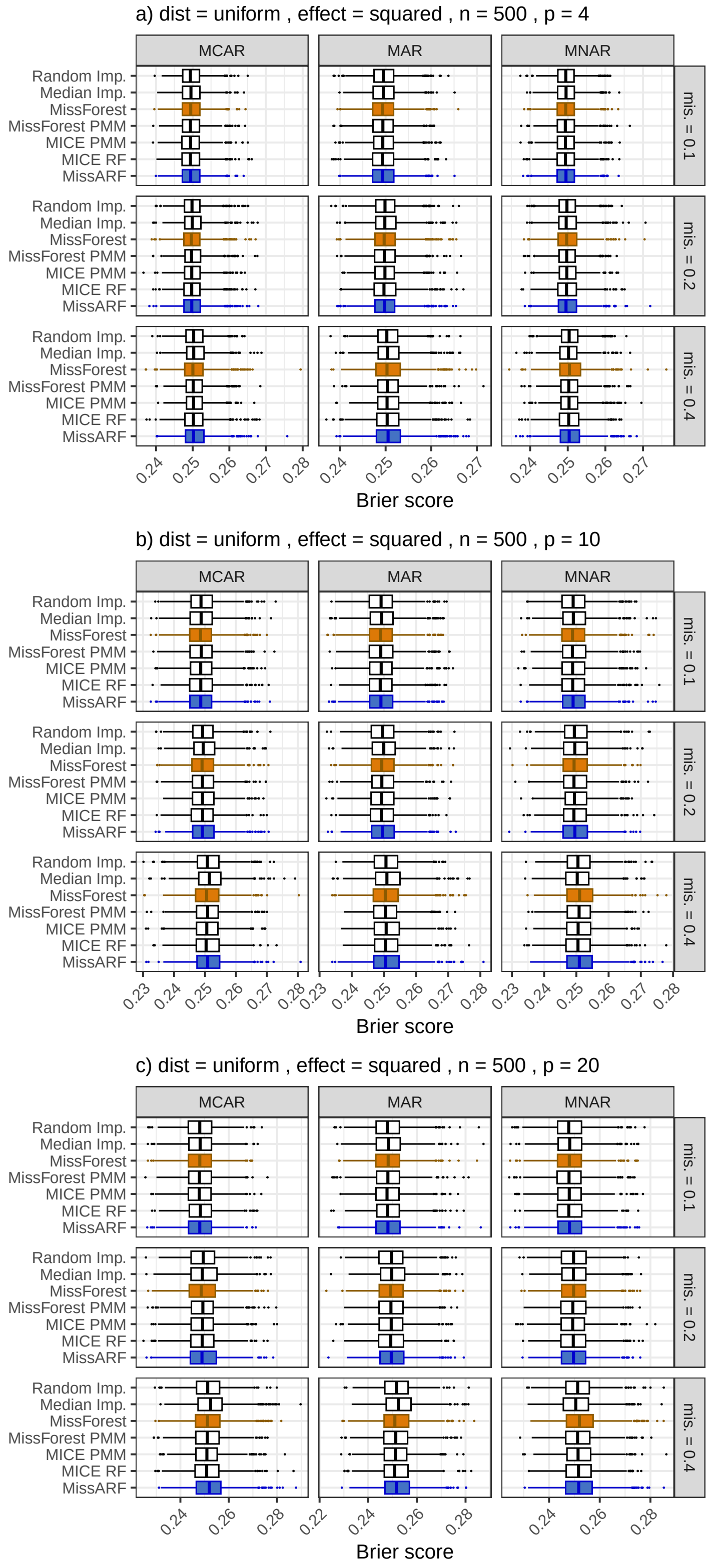

Figure S33: **Brier Score** of the uniform distribution setting with a squared effect over different missingness patterns, dimensionality (p) and missingness rates (mis.) with $n = 500$. The boxplots are plotted over the replicates, with MissARF (blue) and MissForest (orange) highlighted.

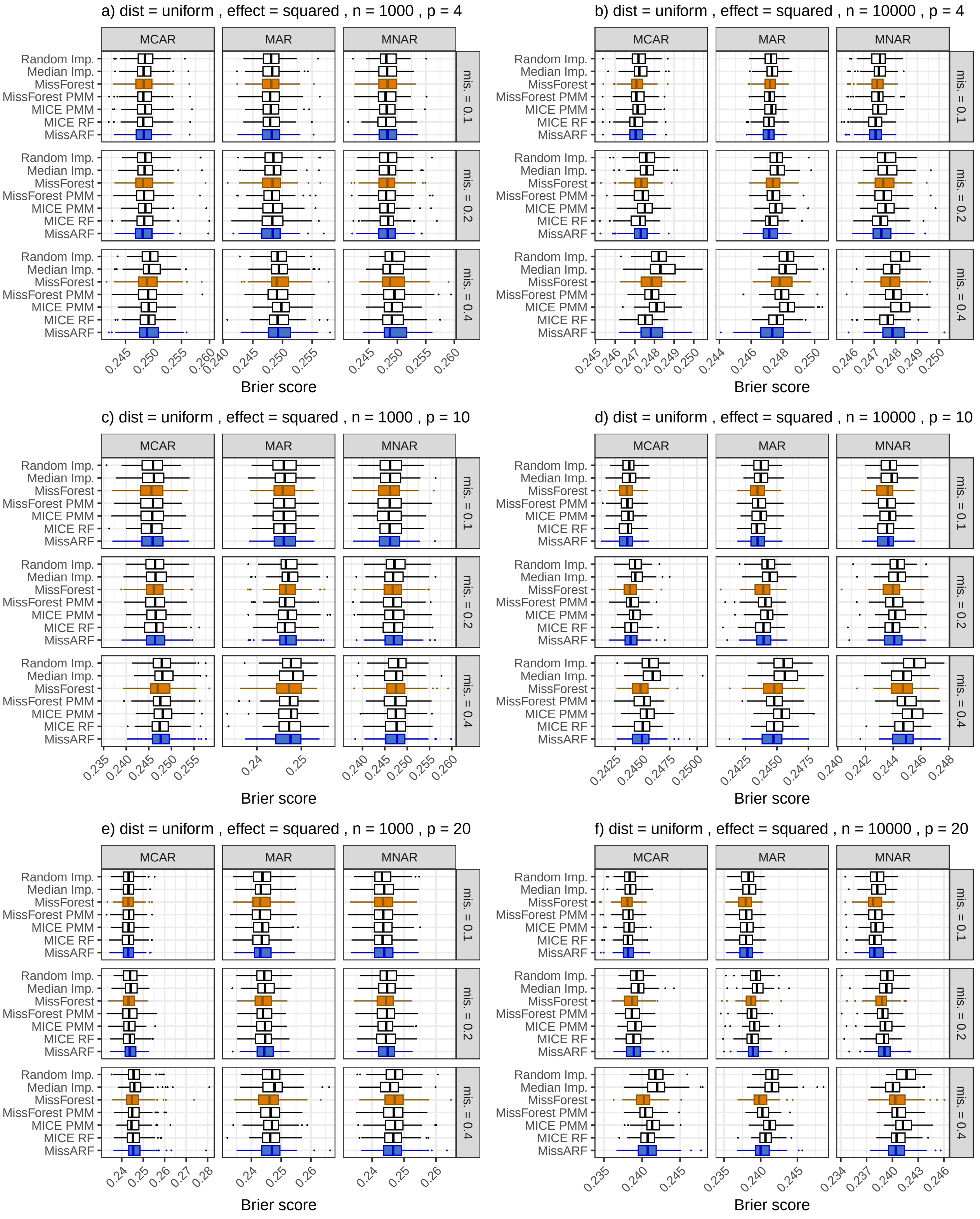

Figure S34: **Brier Score** of the uniform distribution setting with a squared effect over different missingness patterns, dimensionality (p) and missingness rates (mis.) with $n = 1000$ (left) and $n = 10,000$ (right). The boxplots are plotted over the replicates, with MissARF (blue) and MissForest (orange) highlighted.

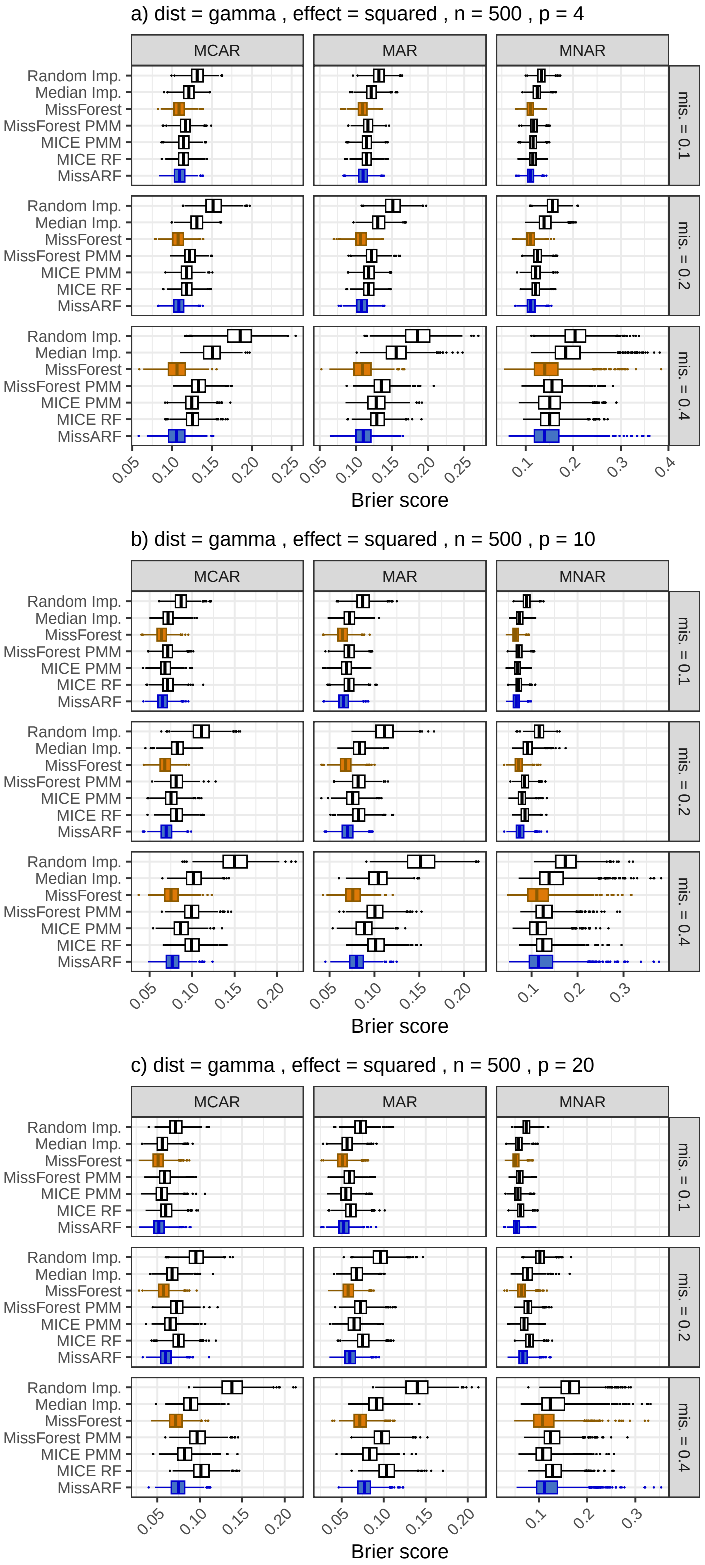

Figure S35: **Brier Score** of the gamma distribution setting with a squared effect over different missingness patterns, dimensionality (p) and missingness rates (mis.) with $n = 500$. The boxplots are plotted over the replicates, with MissARF (blue) and MissForest (orange) highlighted.

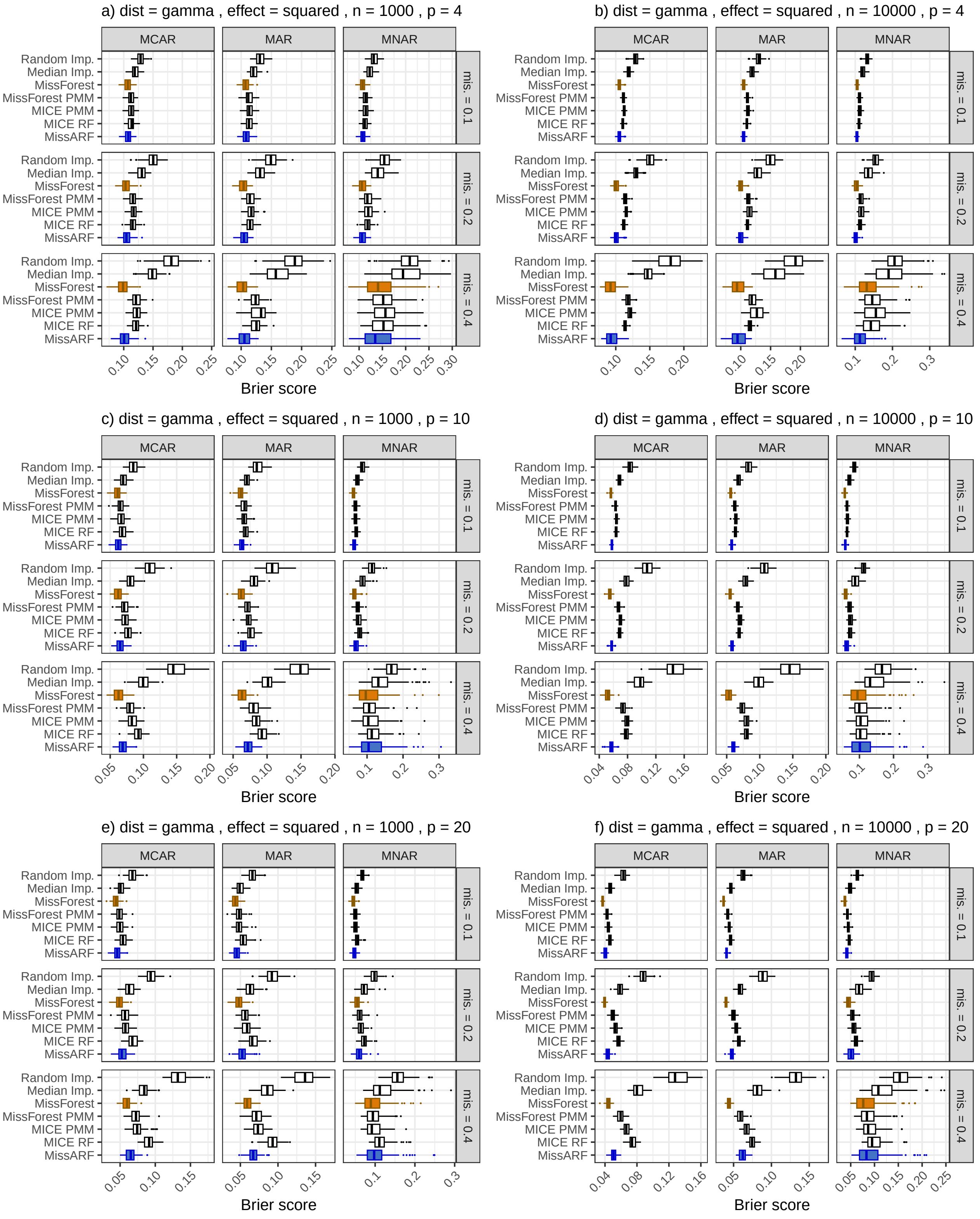

Figure S36: **Brier Score** of the gamma distribution setting with a squared effect over different missingness patterns, dimensionality (p) and missingness rates (mis.) with $n = 1000$ (left) and $n = 10,000$ (right). The boxplots are plotted over the replicates, with MissARF (blue) and MissForest (orange) highlighted.

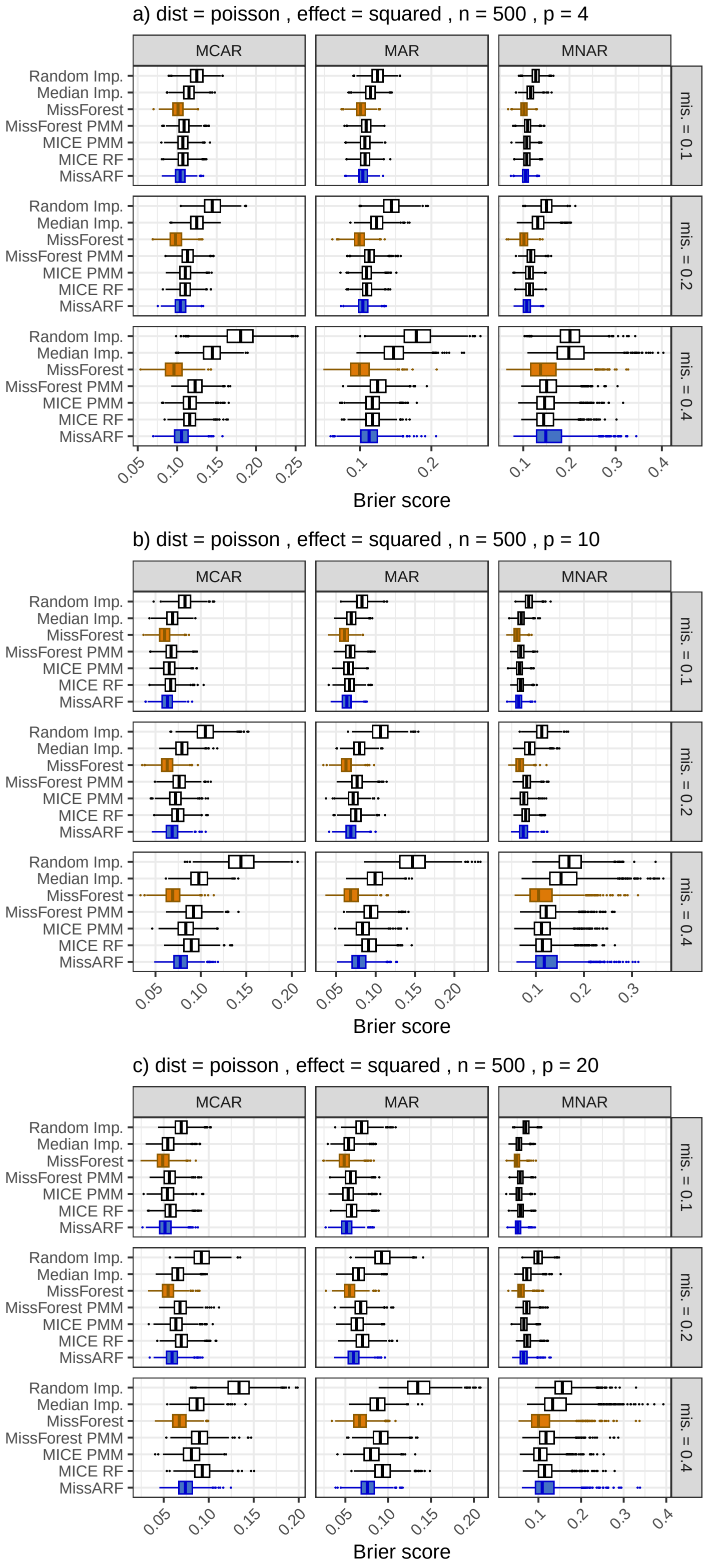

Figure S37: **Brier Score** of the Poisson distribution setting with a squared effect over different missingness patterns, dimensionality (p) and missingness rates (mis.) with $n = 500$. The boxplots are plotted over the replicates, with MissARF (blue) and MissForest (orange) highlighted.

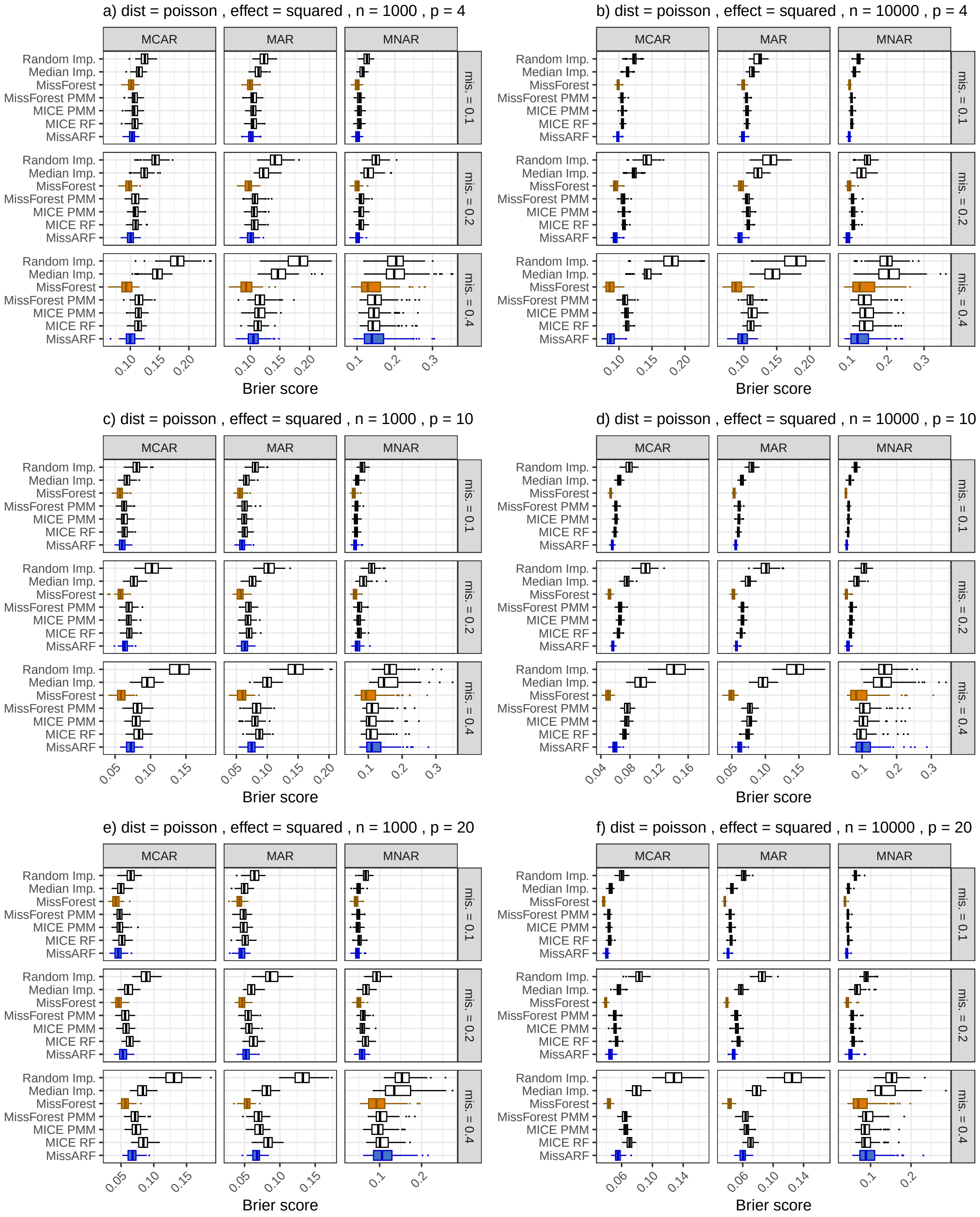

Figure S38: **Brier Score** of the Poisson distribution setting with a squared effect over different missingness patterns, dimensionality (p) and missingness rates (mis.) with $n = 1000$ (left) and $n = 10,000$ (right). The boxplots are plotted over the replicates, with MissARF (blue) and MissForest (orange) highlighted.

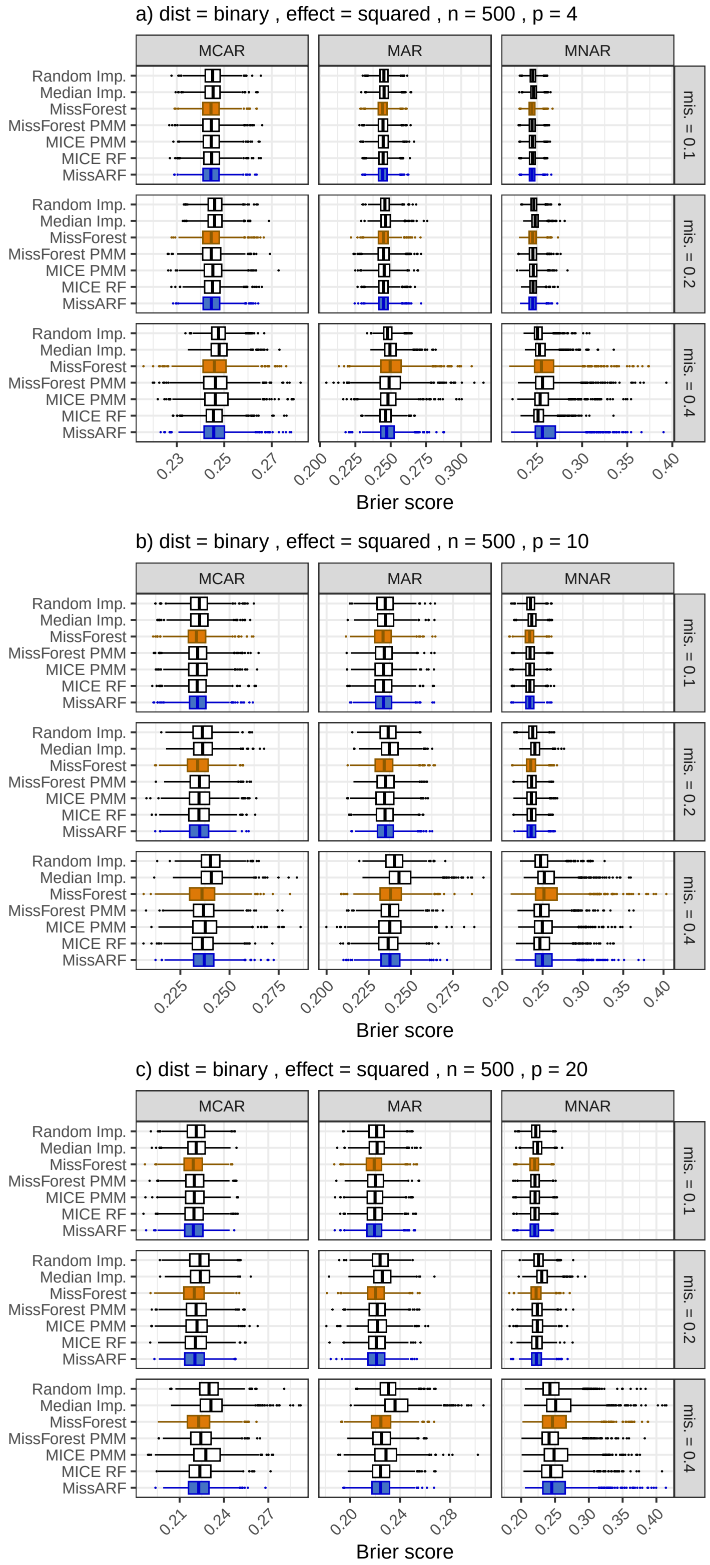

Figure S39: **Brier Score** of the binary distribution setting with a squared effect over different missingness patterns, dimensionality (p) and missingness rates (mis.) with $n = 500$. The boxplots are plotted over the replicates, with MissARF (blue) and MissForest (orange) highlighted.

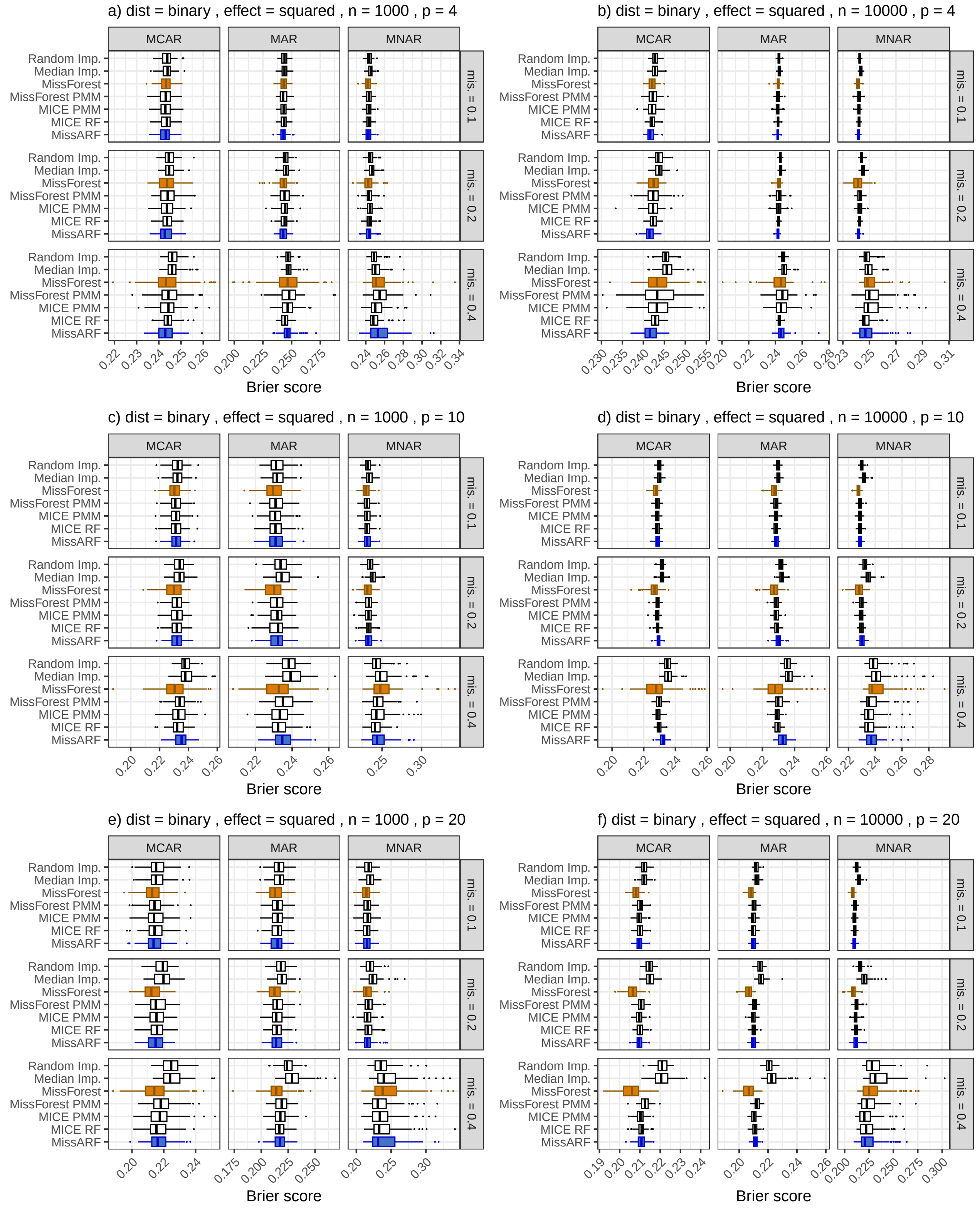

Figure S40: **Brier Score** of the binary distribution setting with a squared effect over different missingness patterns, dimensionality (p) and missingness rates (mis.) with $n = 1000$ (left) and $n = 10,000$ (right). The boxplots are plotted over the replicates, with MissARF (blue) and MissForest (orange) highlighted.

2 Setting II: Multiple imputation

2.1 Coverage rate

2.1.1 Category 1: Similar performance across all methods, MissARF with smallest average width

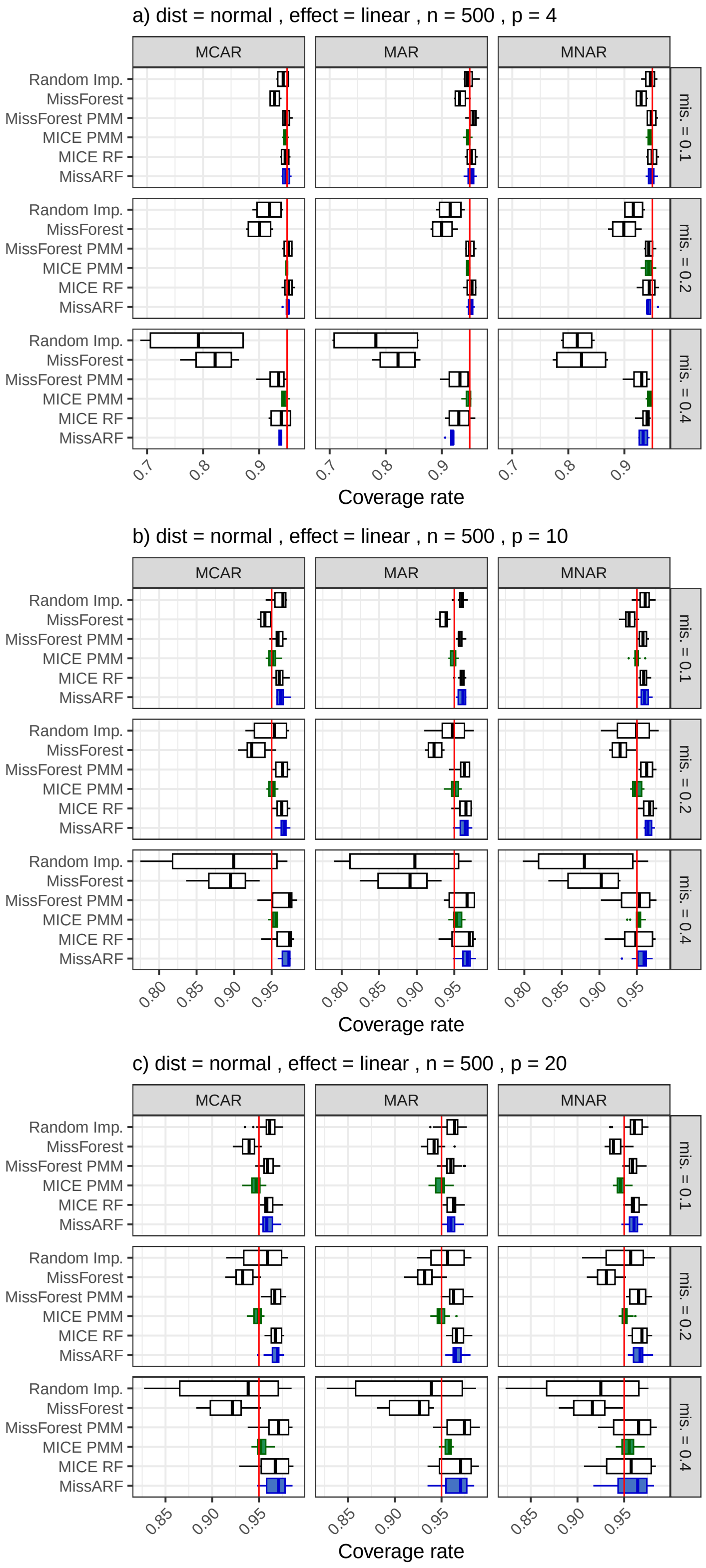

Figure S41: **Coverage rate** of the normal distribution setting with a linear effect over different missingness patterns, dimensionality (p) and missingness rates (mis.) with $n = 500$. The red vertical line shows the nominal coverage level of 0.95. Boxplots are plotted over features, with MissARF (blue) and MICE PMM (green).

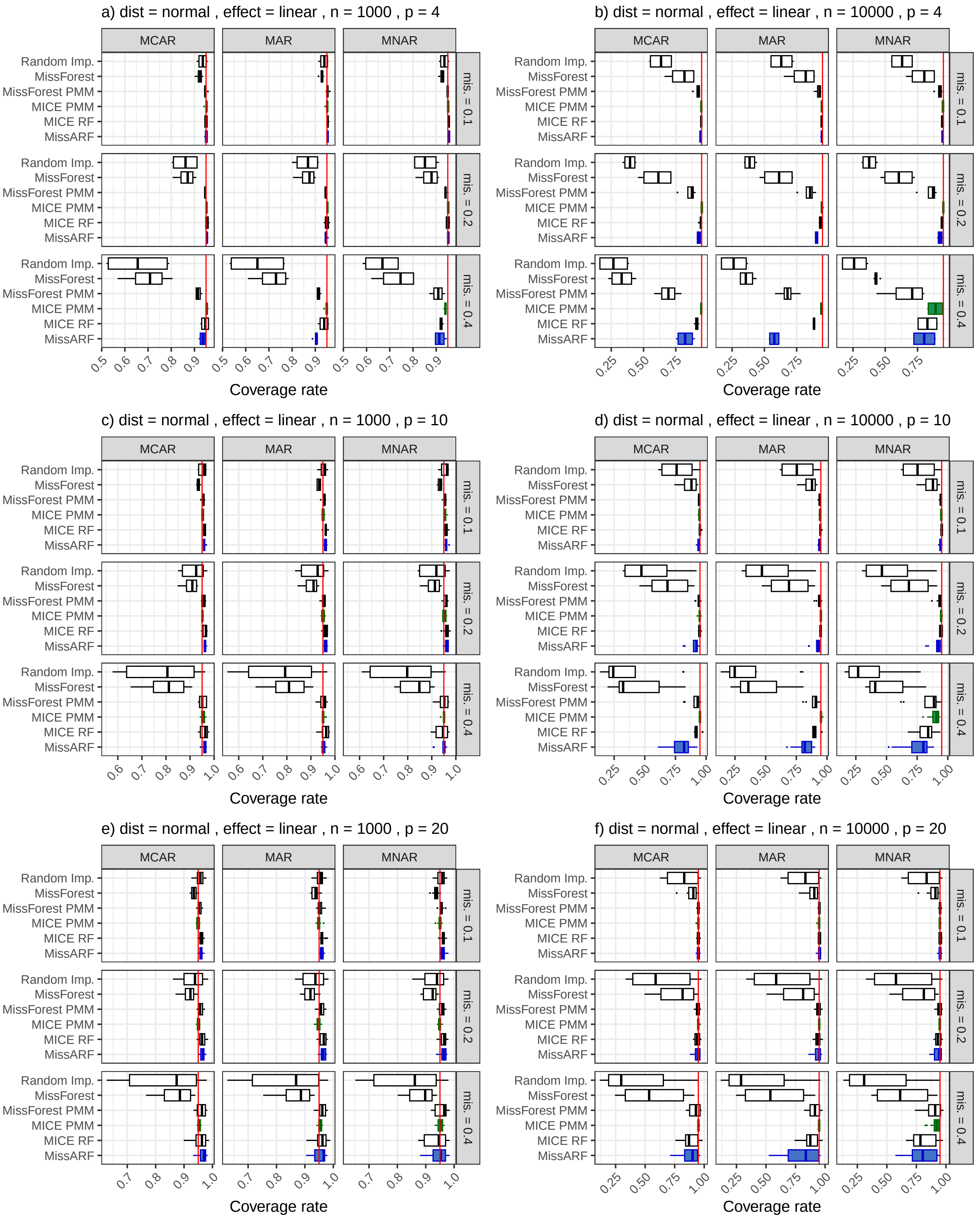

Figure S42: **Coverage rate** of the normal distribution setting with a linear effect over different missingness patterns, dimensionality (p) and missingness rates (mis.) with $n = 1000$ (left) and $n = 10,000$ (right). The red vertical line shows the nominal coverage level of 0.95. Boxplots are plotted over features, with MissARF (blue) and MICE PMM (green).

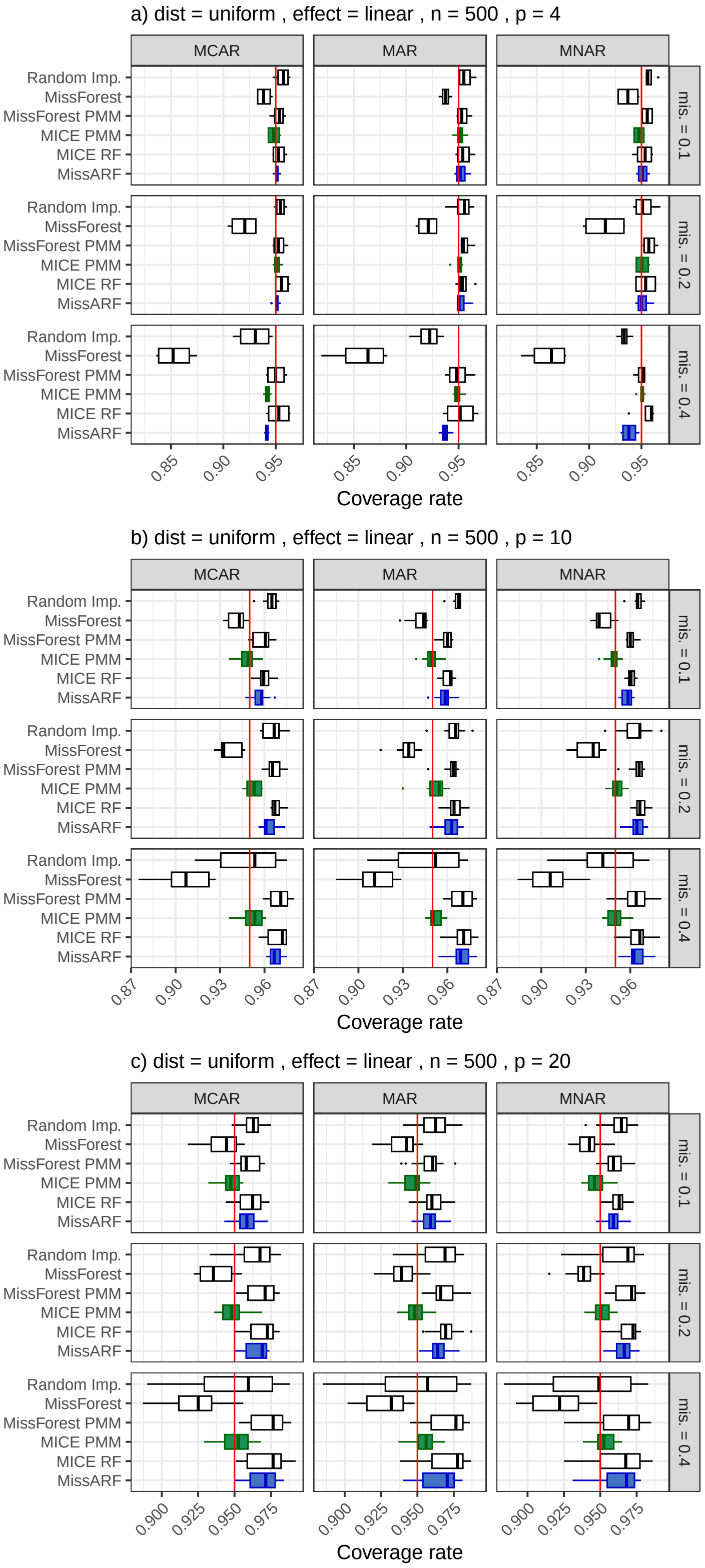

Figure S43: **Coverage rate** of the uniform distribution setting with a linear effect over different missingness patterns, dimensionality (p) and missingness rates (mis.) with $n = 500$. The red vertical line shows the nominal coverage level of 0.95. Boxplots are plotted over features, with MissARF (blue) and MICE PMM (green).

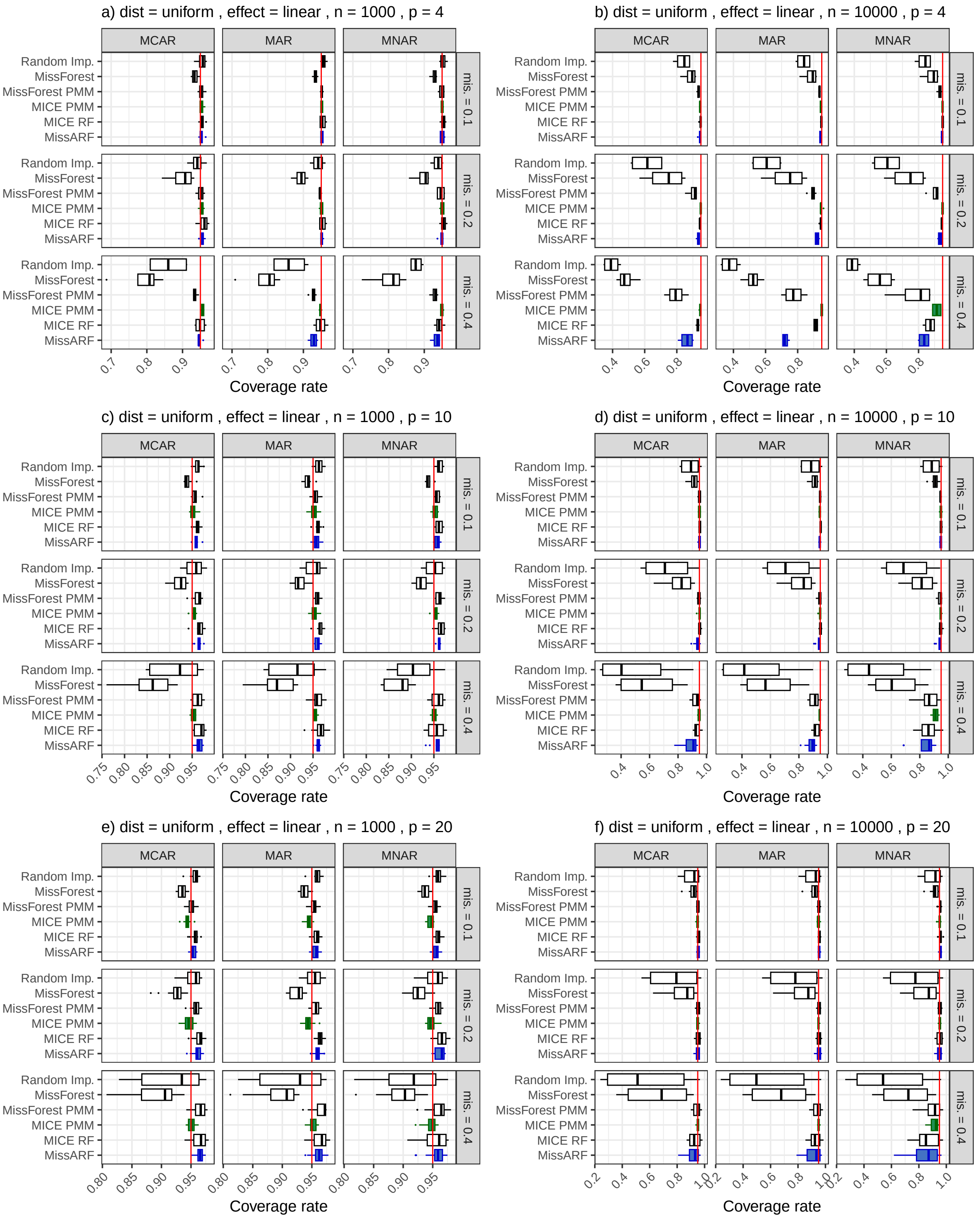

Figure S44: **Coverage rate** of the uniform distribution setting with a linear effect over different missingness patterns, dimensionality (p) and missingness rates (mis.) with $n = 1000$ (left) and $n = 10,000$ (right). The red vertical line shows the nominal coverage level of 0.95. Boxplots are plotted over features, with MissARF (blue) and MICE PMM (green).

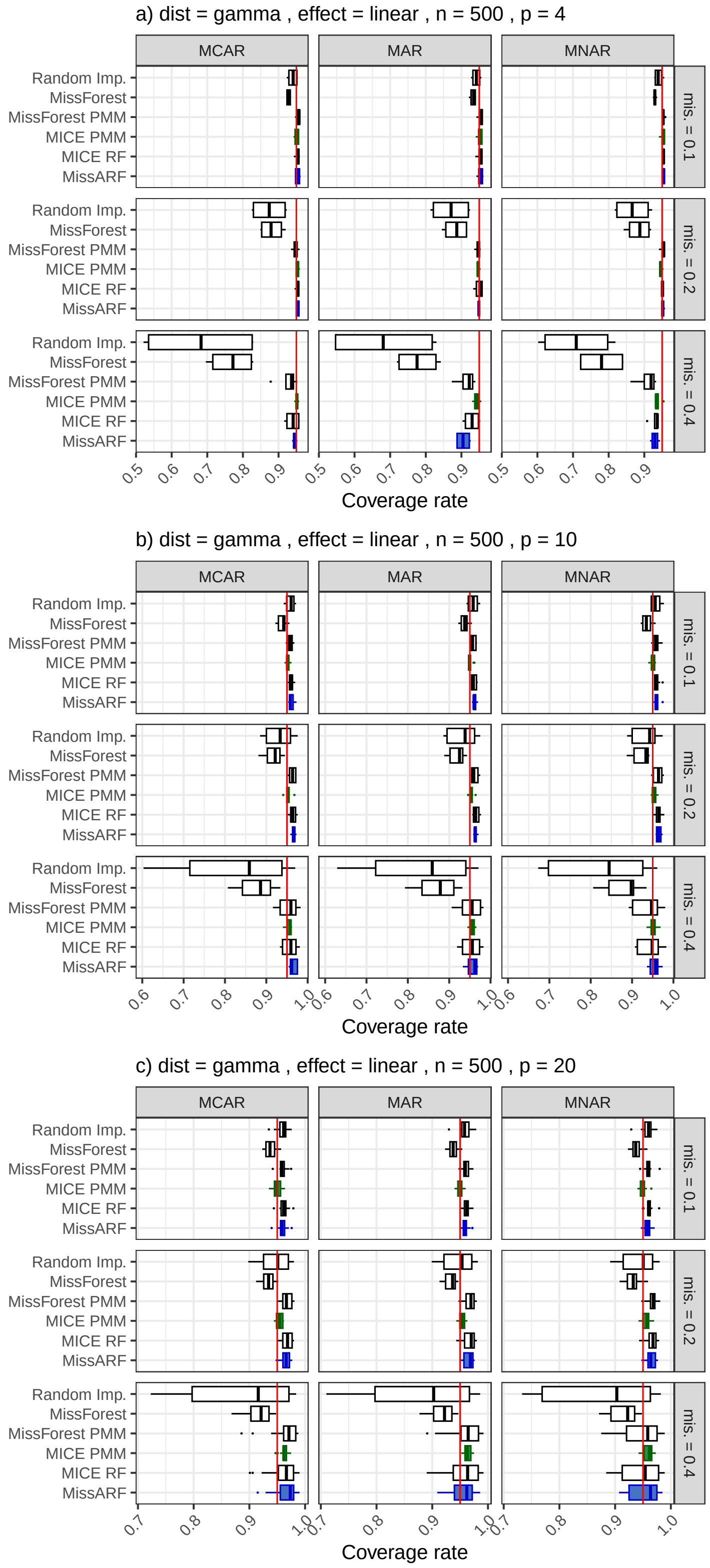

Figure S45: **Coverage rate** of the gamma distribution setting with a linear effect over different missingness patterns, dimensionality (p) and missingness rates (mis.) with $n = 500$. The red vertical line shows the nominal coverage level of 0.95. Boxplots are plotted over features, with MissARF (blue) and MICE PMM (green).

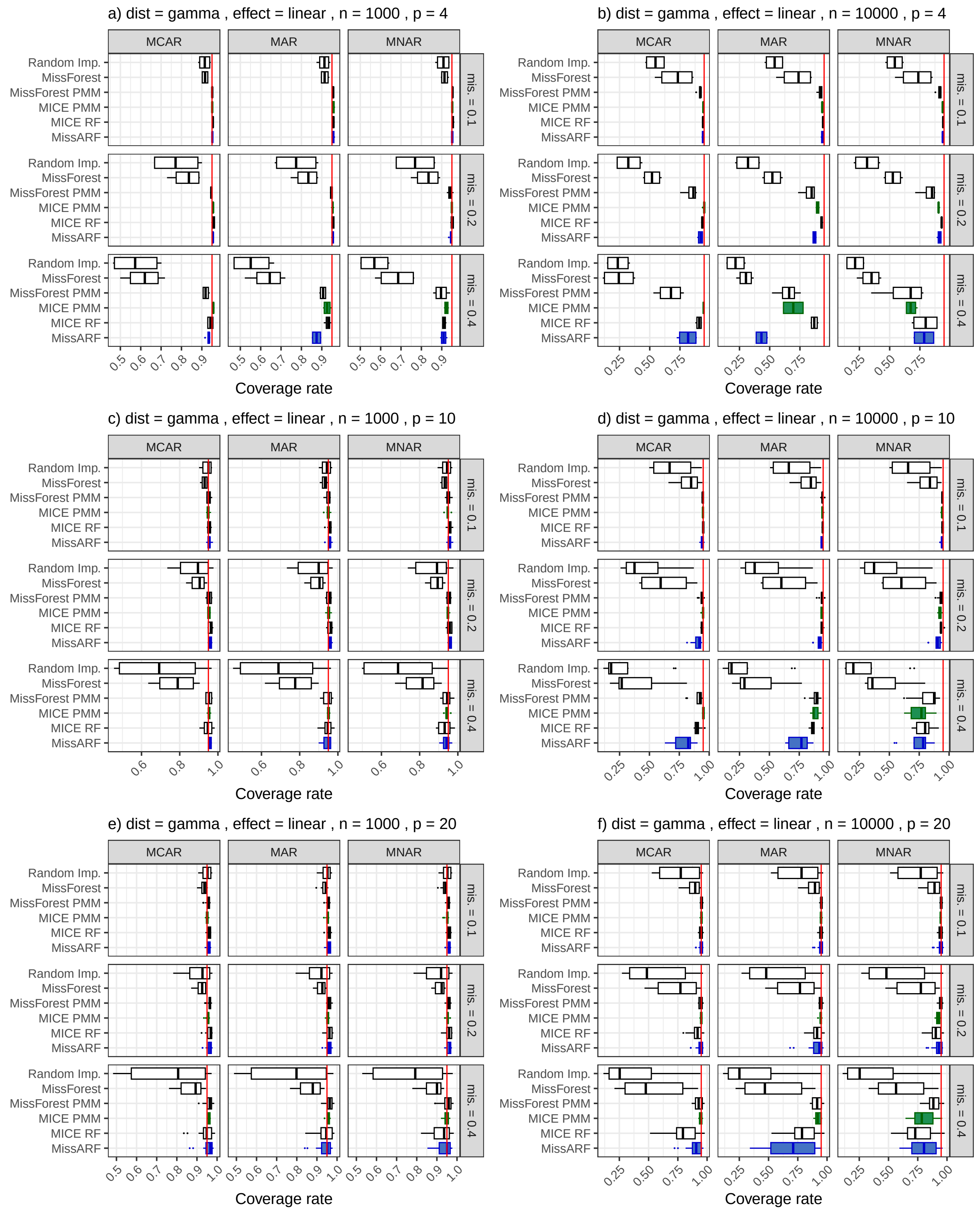

Figure S46: **Coverage rate** of the gamma distribution setting with a linear effect over different missingness patterns, dimensionality (p) and missingness rates (mis.) with $n = 1000$ (left) and $n = 10,000$ (right). The red vertical line shows the nominal coverage level of 0.95. Boxplots are plotted over features, with MissARF (blue) and MICE PMM (green).

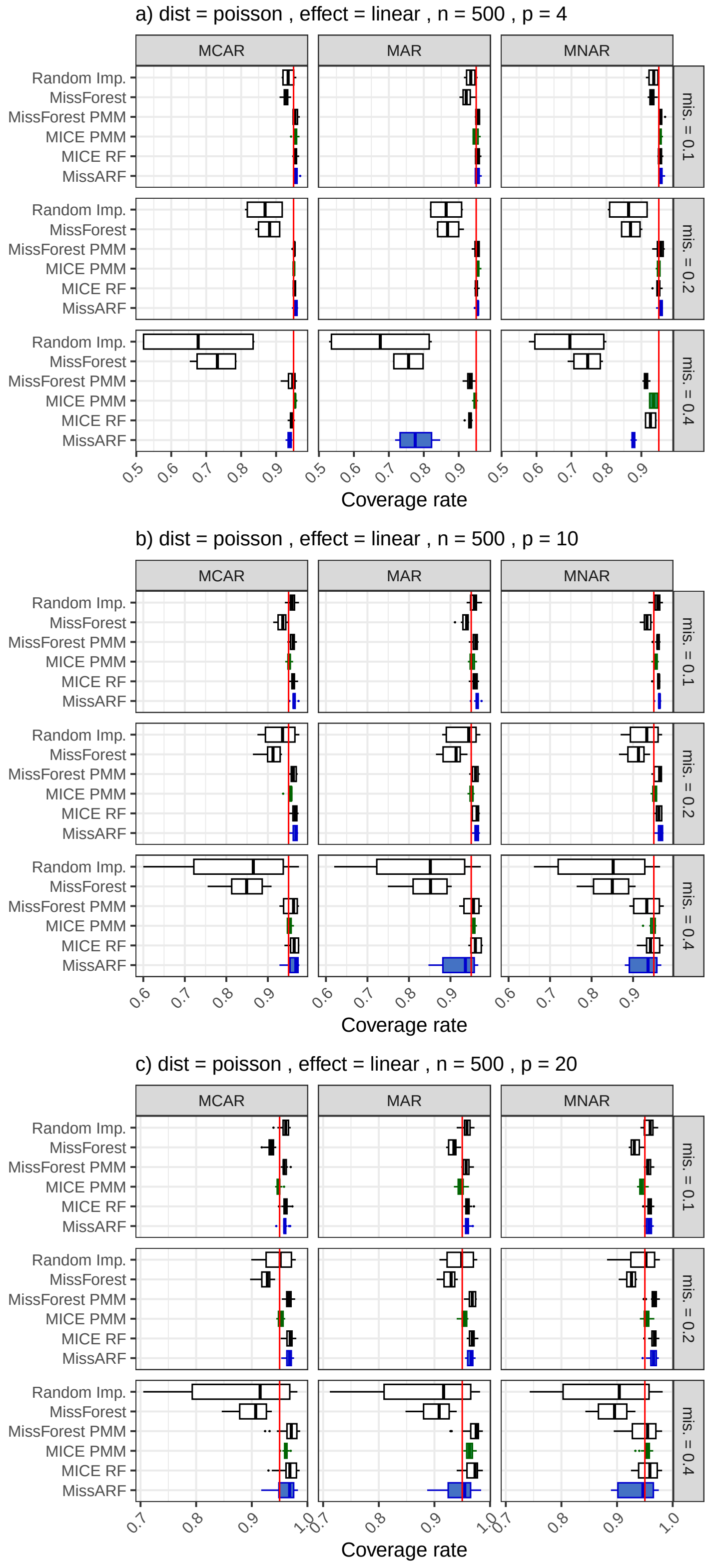

Figure S47: **Coverage rate** of the Poisson distribution setting with a linear effect over different missingness patterns, dimensionality (p) and missingness rates (mis.) with $n = 500$. The red vertical line shows the nominal coverage level of 0.95. Boxplots are plotted over features, with MissARF (blue) and MICE PMM (green).

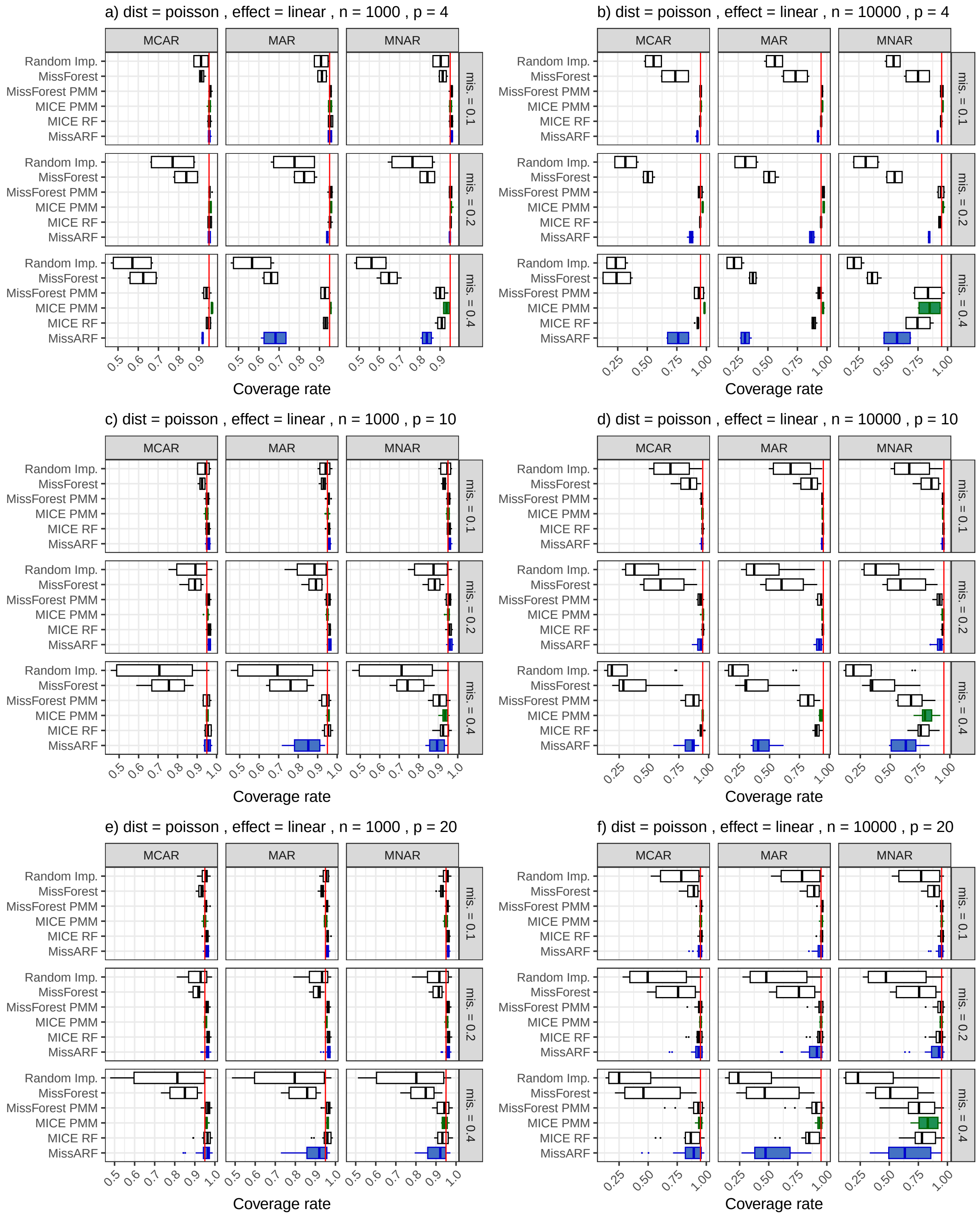

Figure S48: **Coverage rate** of the Poisson distribution setting with a linear effect over different missingness patterns, dimensionality (p) and missingness rates (mis.) with $n = 1000$ (left) and $n = 10,000$ (right). The red vertical line shows the nominal coverage level of 0.95. Boxplots are plotted over features, with MissARF (blue) and MICE PMM (green).

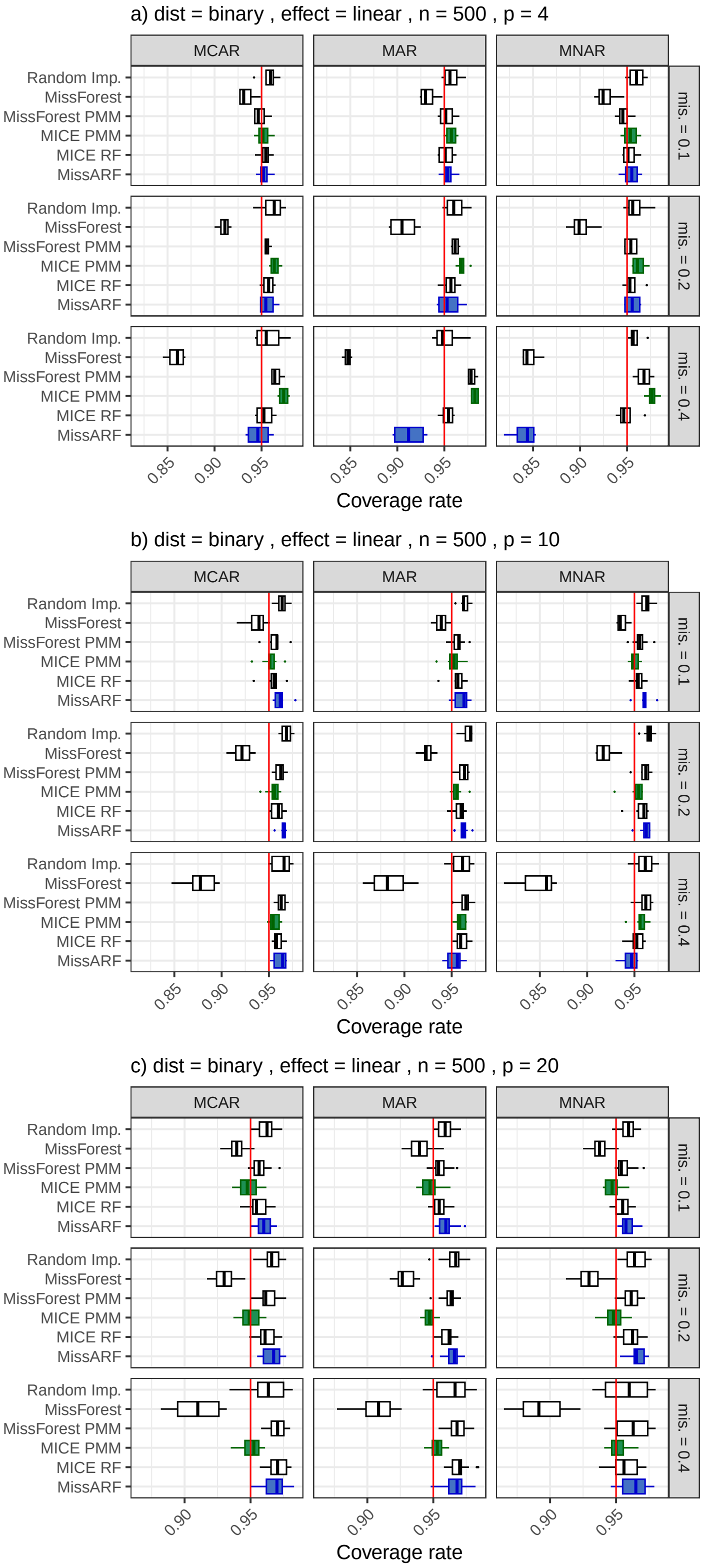

Figure S49: **Coverage rate** of the binary distribution setting with a linear effect over different missingness patterns, dimensionality (p) and missingness rates (mis.) with $n = 500$. The red vertical line shows the nominal coverage level of 0.95. Boxplots are plotted over features, with MissARF (blue) and MICE PMM (green).

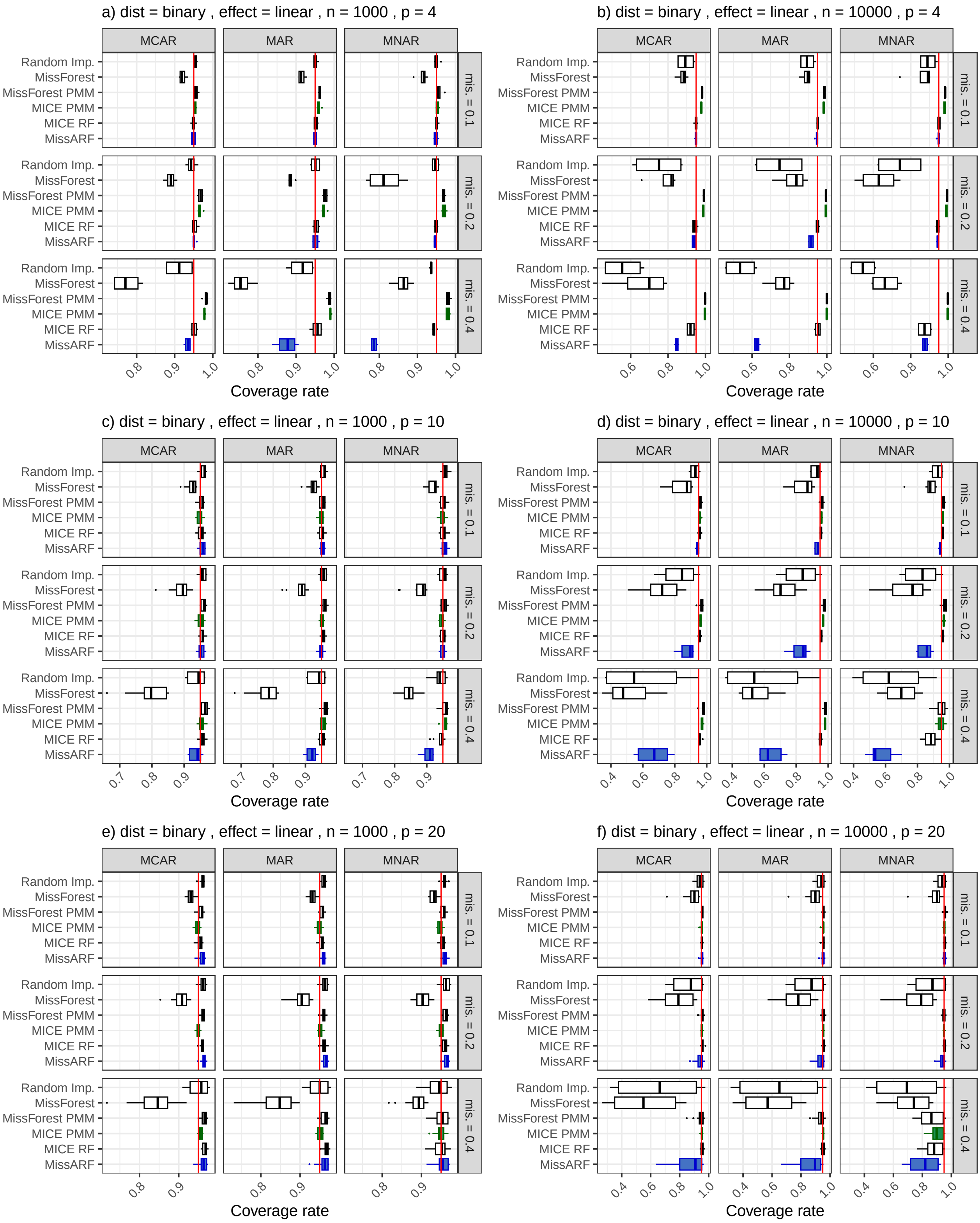

Figure S50: **Coverage rate** of the binary distribution setting with a linear effect over different missingness patterns, dimensionality (p) and missingness rates (mis.) with $n = 1000$ (left) and $n = 10,000$ (right). The red vertical line shows the nominal coverage level of 0.95. Boxplots are plotted over features, with MissARF (blue) and MICE PMM (green).

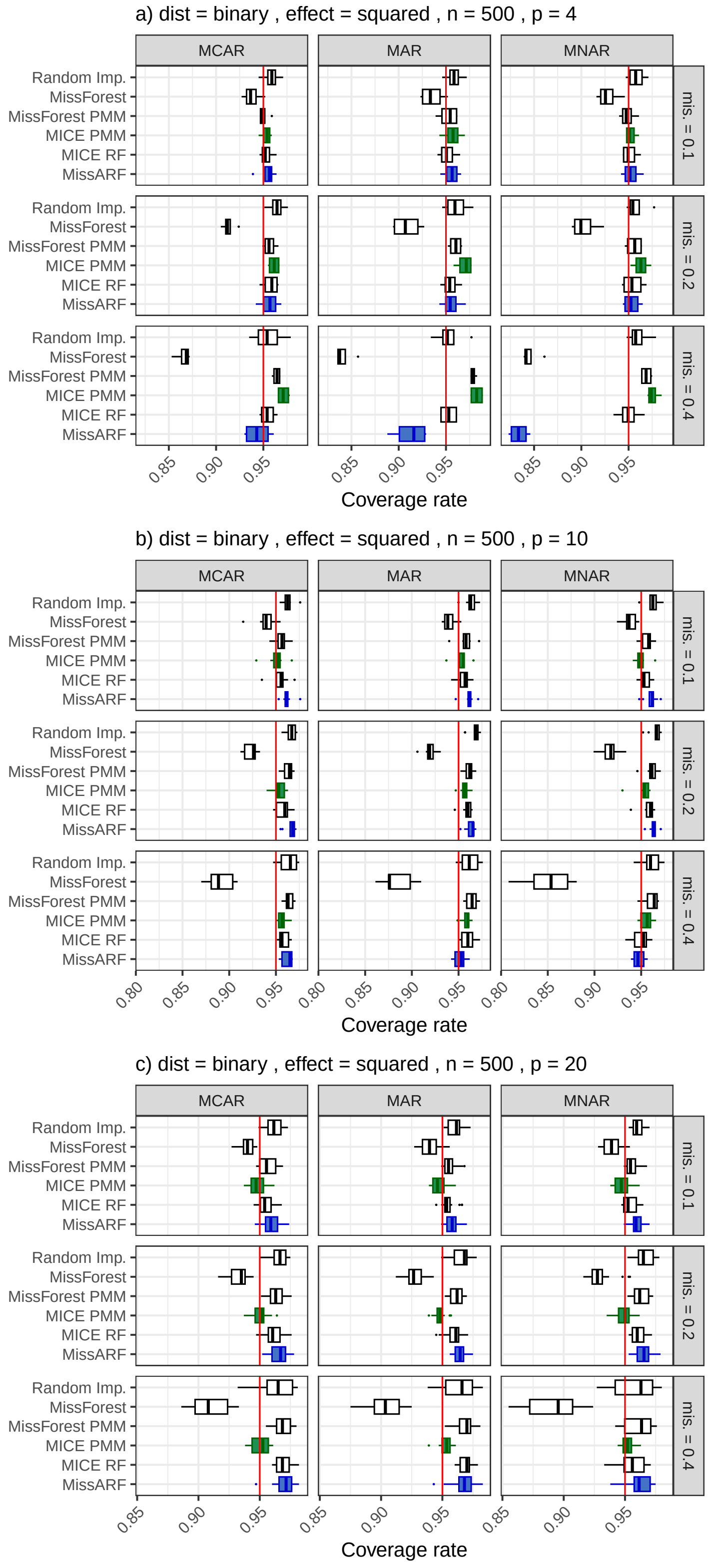

Figure S51: **Coverage rate** of the binary distribution setting with a squared effect over different missingness patterns, dimensionality (p) and missingness rates (mis.) with $n = 500$. The red vertical line shows the nominal coverage level of 0.95. Boxplots are plotted over features, with MissARF (blue) and MICE PMM (green).

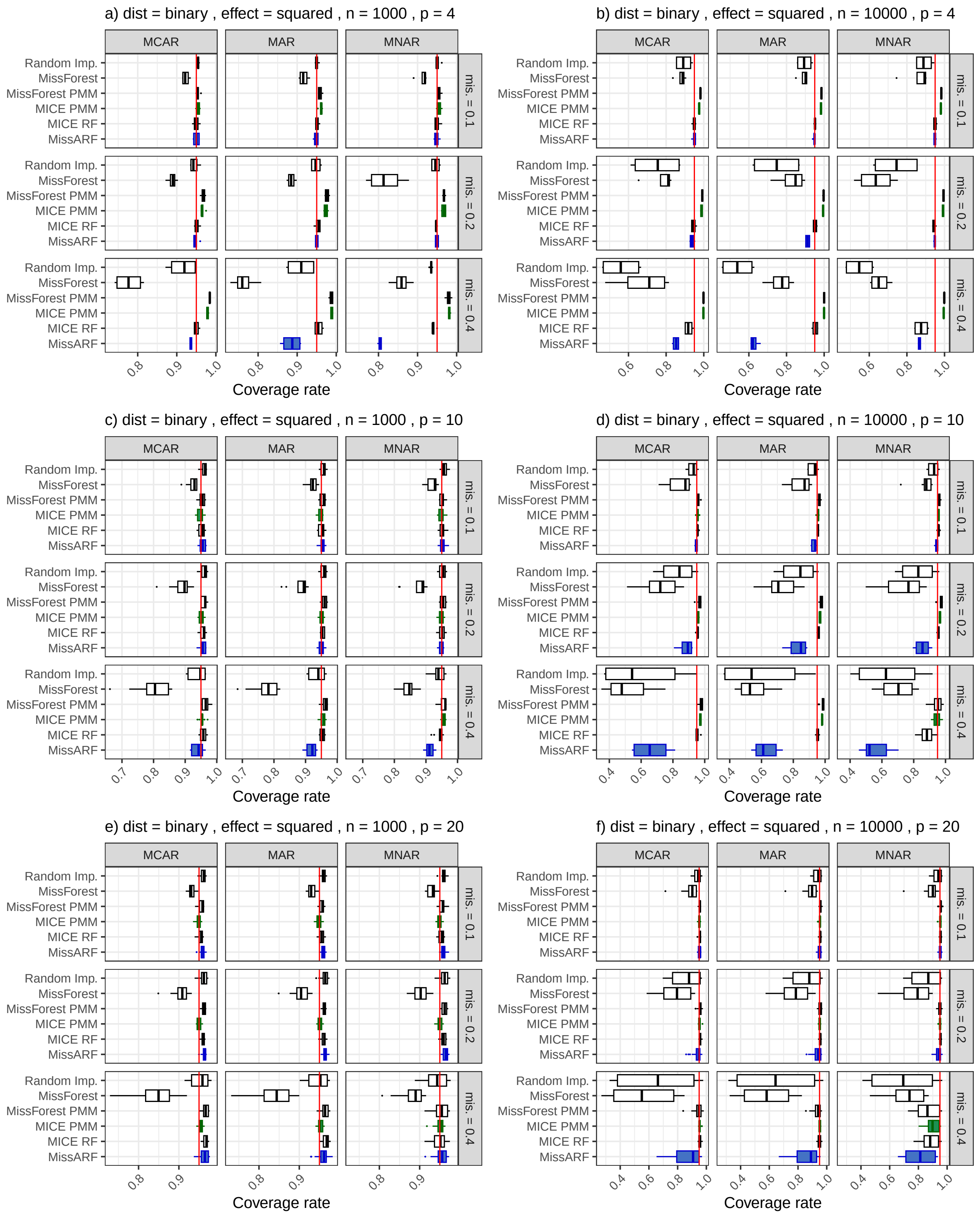

Figure S52: **Coverage rate** of the binary distribution setting with a squared effect over different missingness patterns, dimensionality (p) and missingness rates (mis.) with $n = 1000$ (left) and $n = 10,000$ (right). The red vertical line shows the nominal coverage level of 0.95. Boxplots are plotted over features, with MissARF (blue) and MICE PMM (green).

2.1.2 Category 2: PMM methods struggle, MissARF performs well

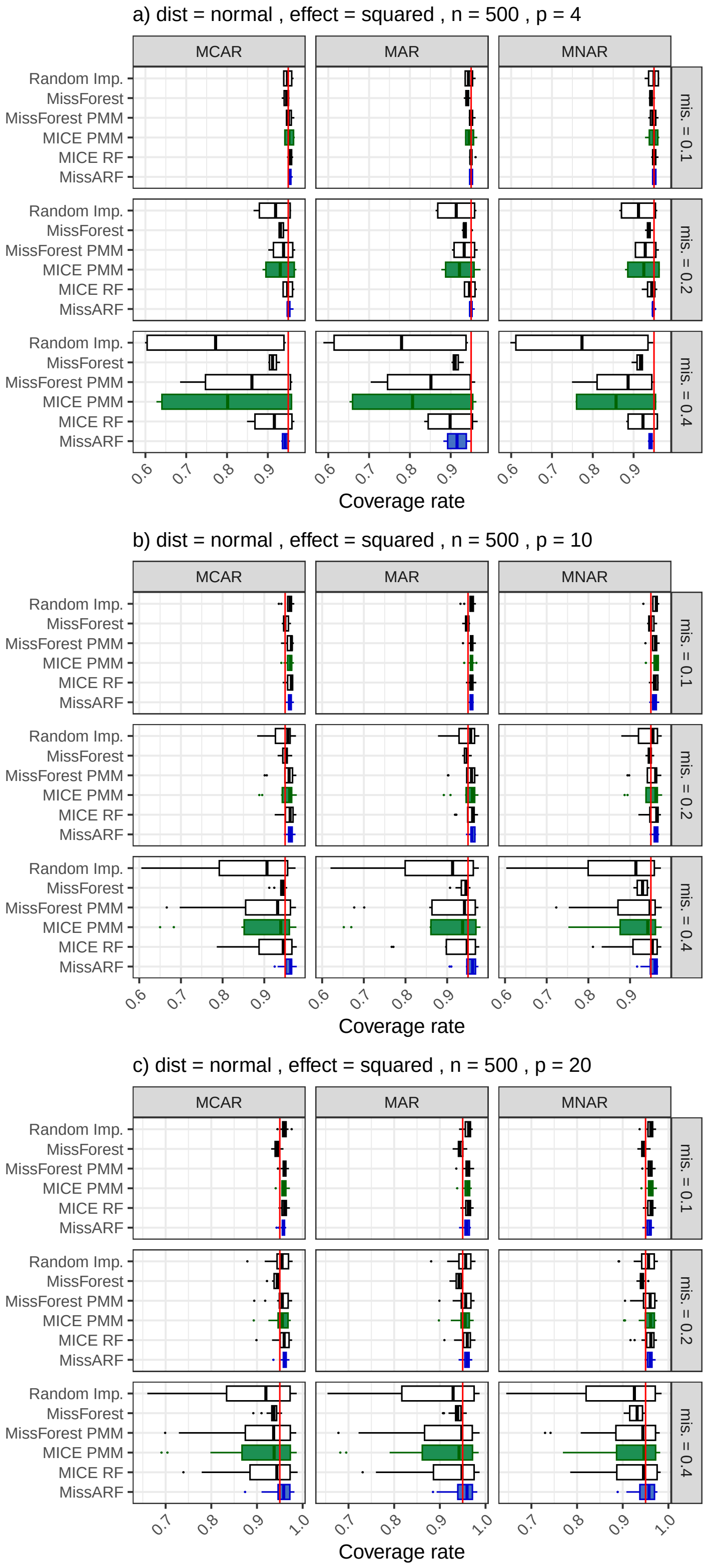

Figure S53: **Coverage rate** of the normal distribution setting with a squared effect over different missingness patterns, dimensionality (p) and missingness rates (mis.) with $n = 500$. The red vertical line shows the nominal coverage level of 0.95. Boxplots are plotted over features, with MissARF (blue) and MICE PMM (green).

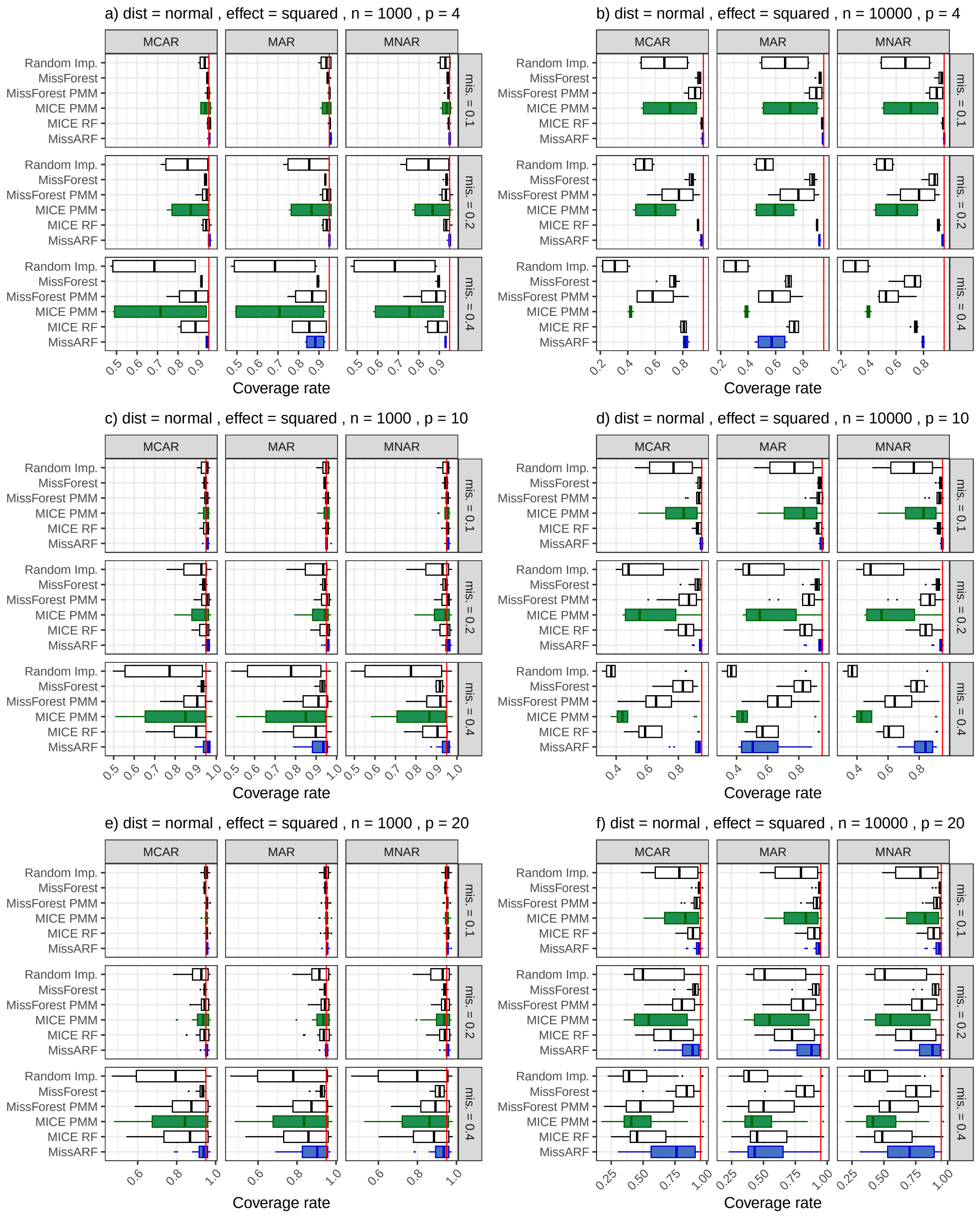

Figure S54: **Coverage rate** of the normal distribution setting with a squared effect over different missingness patterns, dimensionality (p) and missingness rates (mis.) with $n = 1000$ (left) and $n = 10,000$ (right). The red vertical line shows the nominal coverage level of 0.95. Boxplots are plotted over features, with MissARF (blue) and MICE PMM (green).

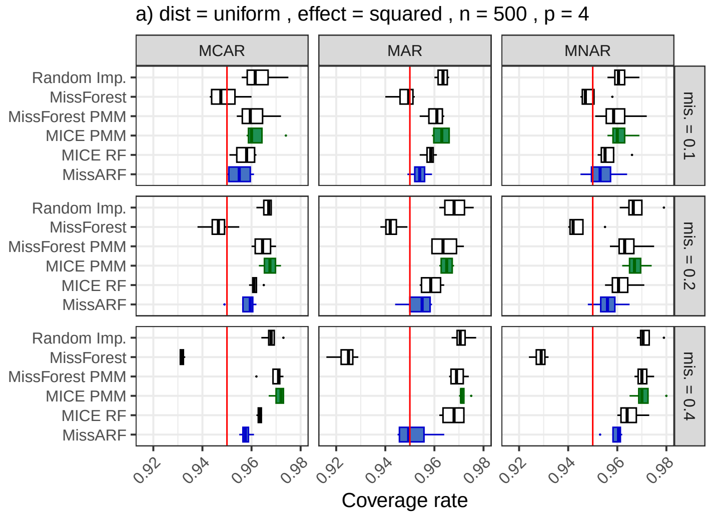

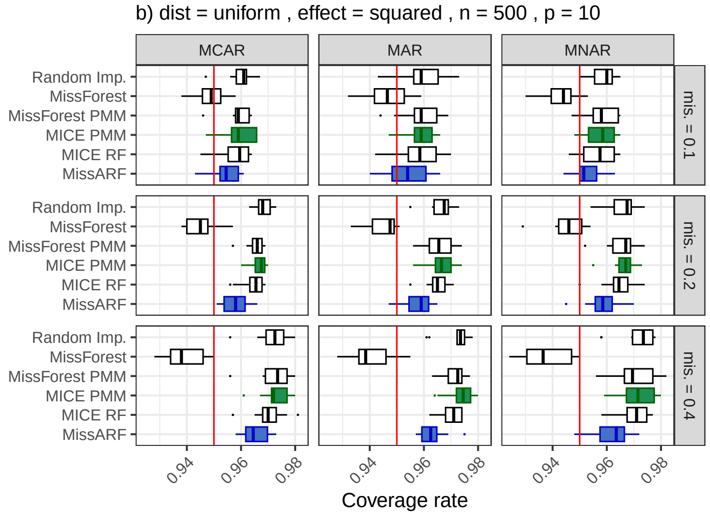

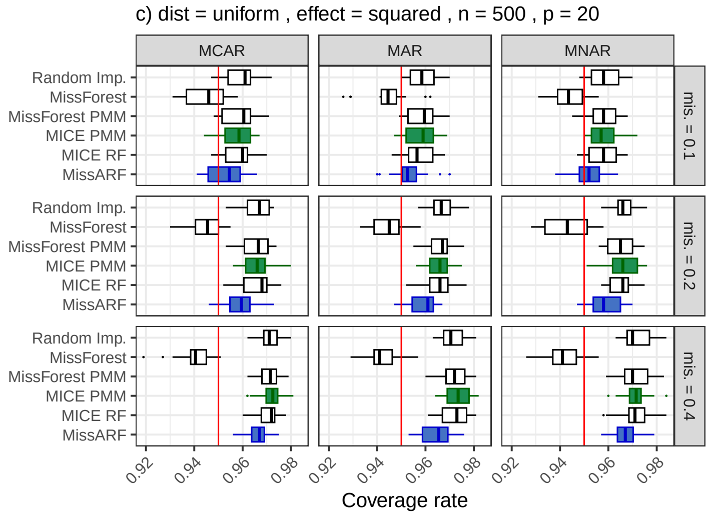

Figure S55: **Coverage rate** of the uniform distribution setting with a squared effect over different missingness patterns, dimensionality (p) and missingness rates (mis.) with $n = 500$. The red vertical line shows the nominal coverage level of 0.95. Boxplots are plotted over features, with MissARF (blue) and MICE PMM (green).

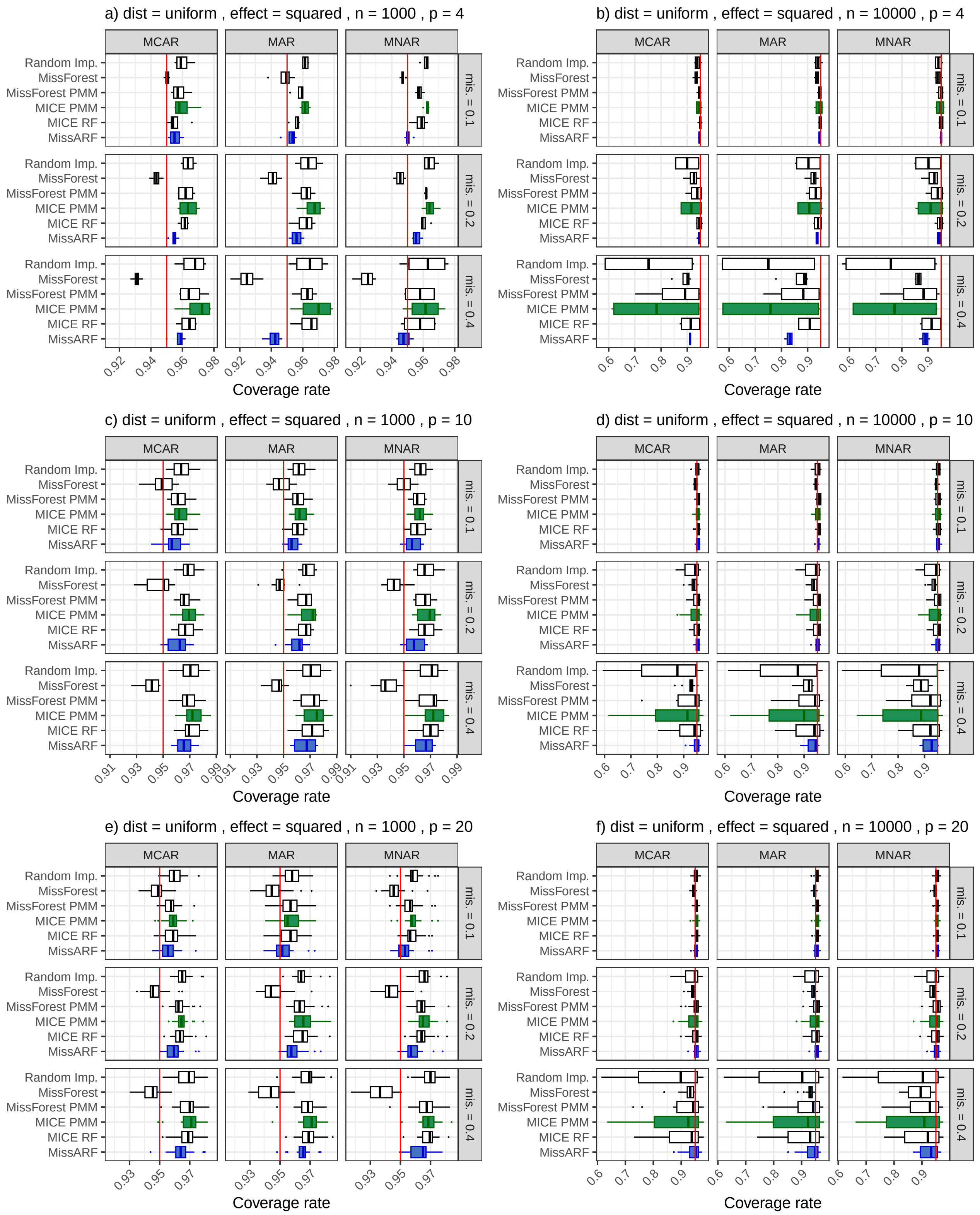

Figure S56: **Coverage rate** of the uniform distribution setting with a squared effect over different missingness patterns, dimensionality (p) and missingness rates (mis.) with $n = 1000$ (left) and $n = 10,000$ (right). The red vertical line shows the nominal coverage level of 0.95. Boxplots are plotted over features, with MissARF (blue) and MICE PMM (green).

2.1.3 Category 3: All methods perform poorly

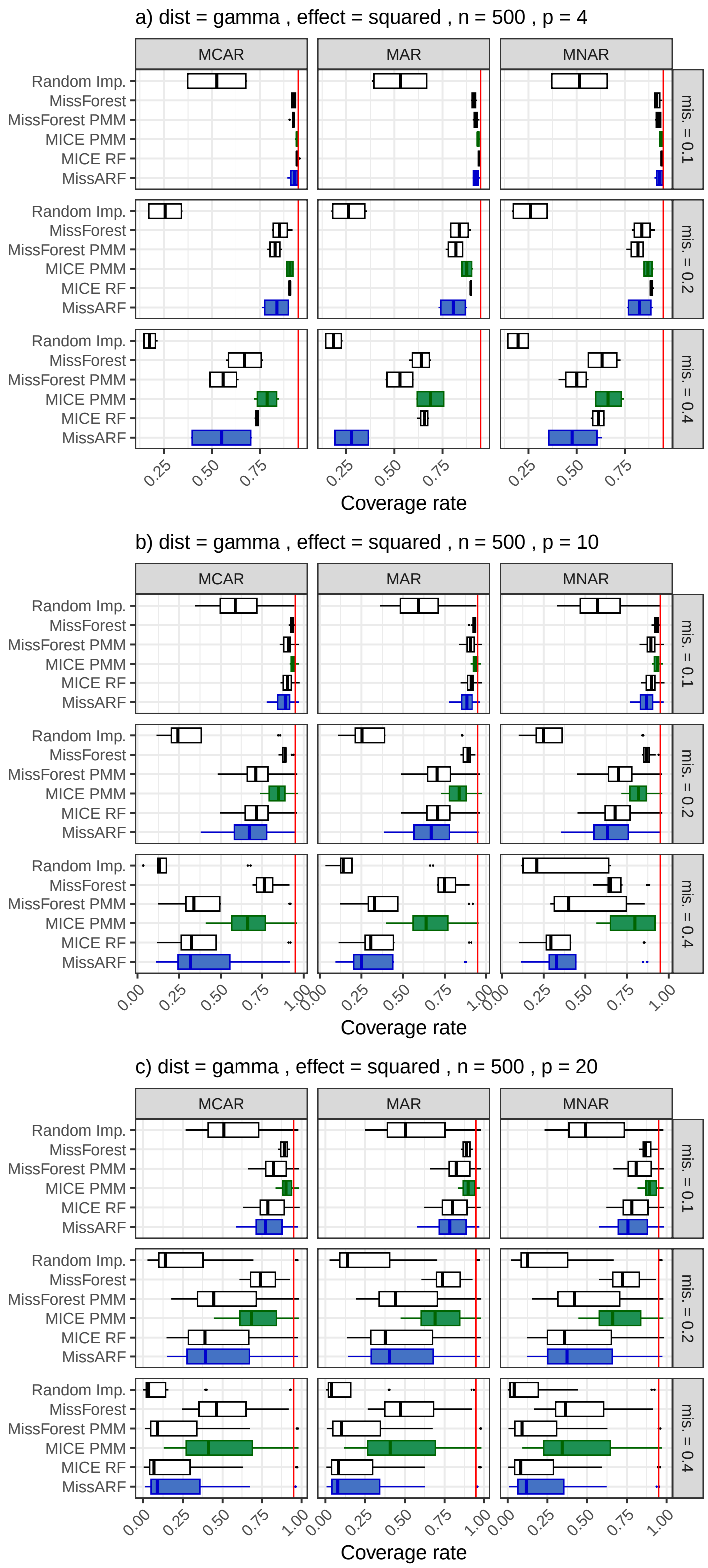

Figure S57: **Coverage rate** of the gamma distribution setting with a squared effect over different missingness patterns, dimensionality (p) and missingness rates (mis.) with $n = 500$. The red vertical line shows the nominal coverage level of 0.95. Boxplots are plotted over features, with MissARF (blue) and MICE PMM (green).

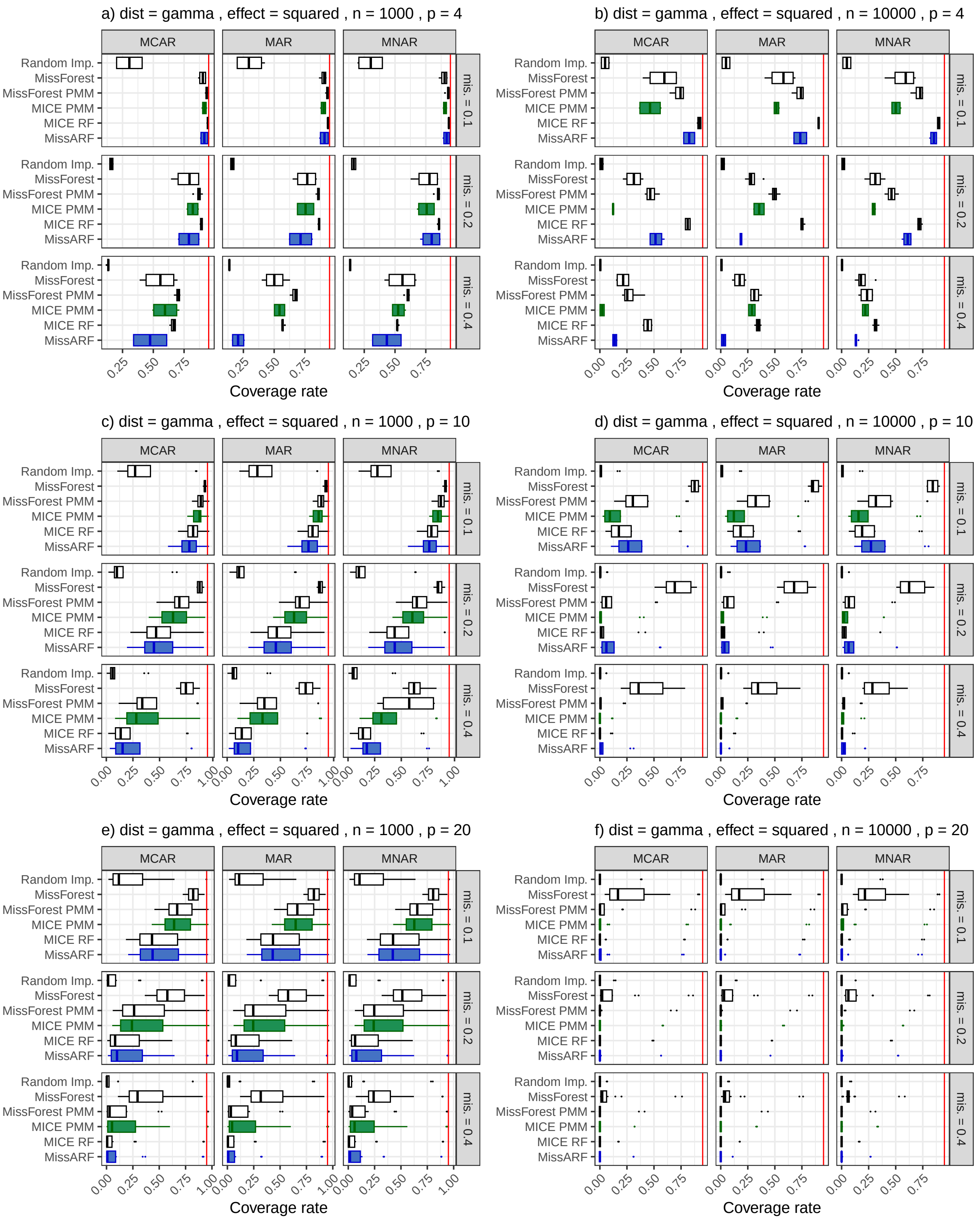

Figure S58: **Coverage rate** of the gamma distribution setting with a squared effect over different missingness patterns, dimensionality (p) and missingness rates (mis.) with $n = 1000$ (left) and $n = 10,000$ (right). The red vertical line shows the nominal coverage level of 0.95. Boxplots are plotted over features, with MissARF (blue) and MICE PMM (green).

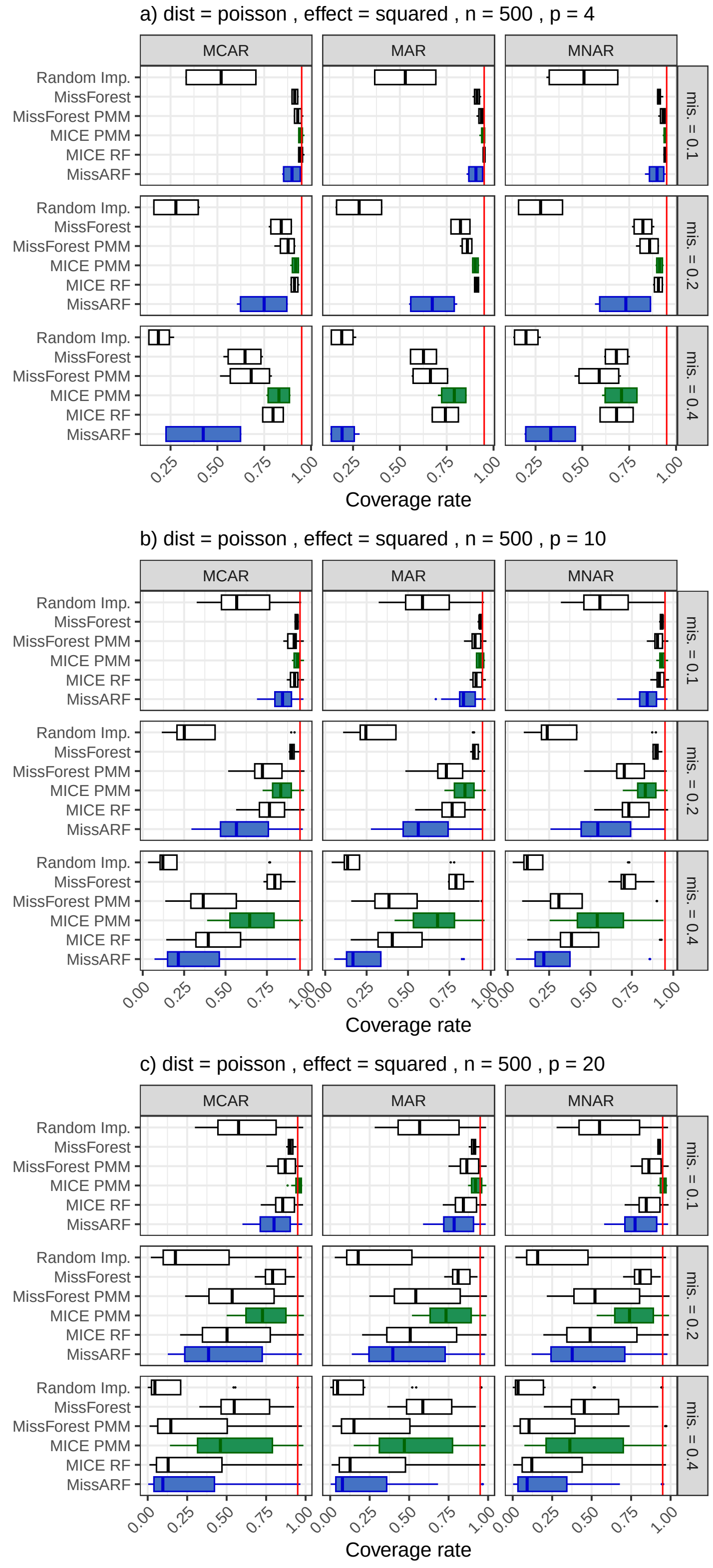

Figure S59: **Coverage rate** of the Poisson distribution setting with a squared effect over different missingness patterns, dimensionality (p) and missingness rates (mis.) with $n = 500$. The red vertical line shows the nominal coverage level of 0.95. Boxplots are plotted over features, with MissARF (blue) and MICE PMM (green).

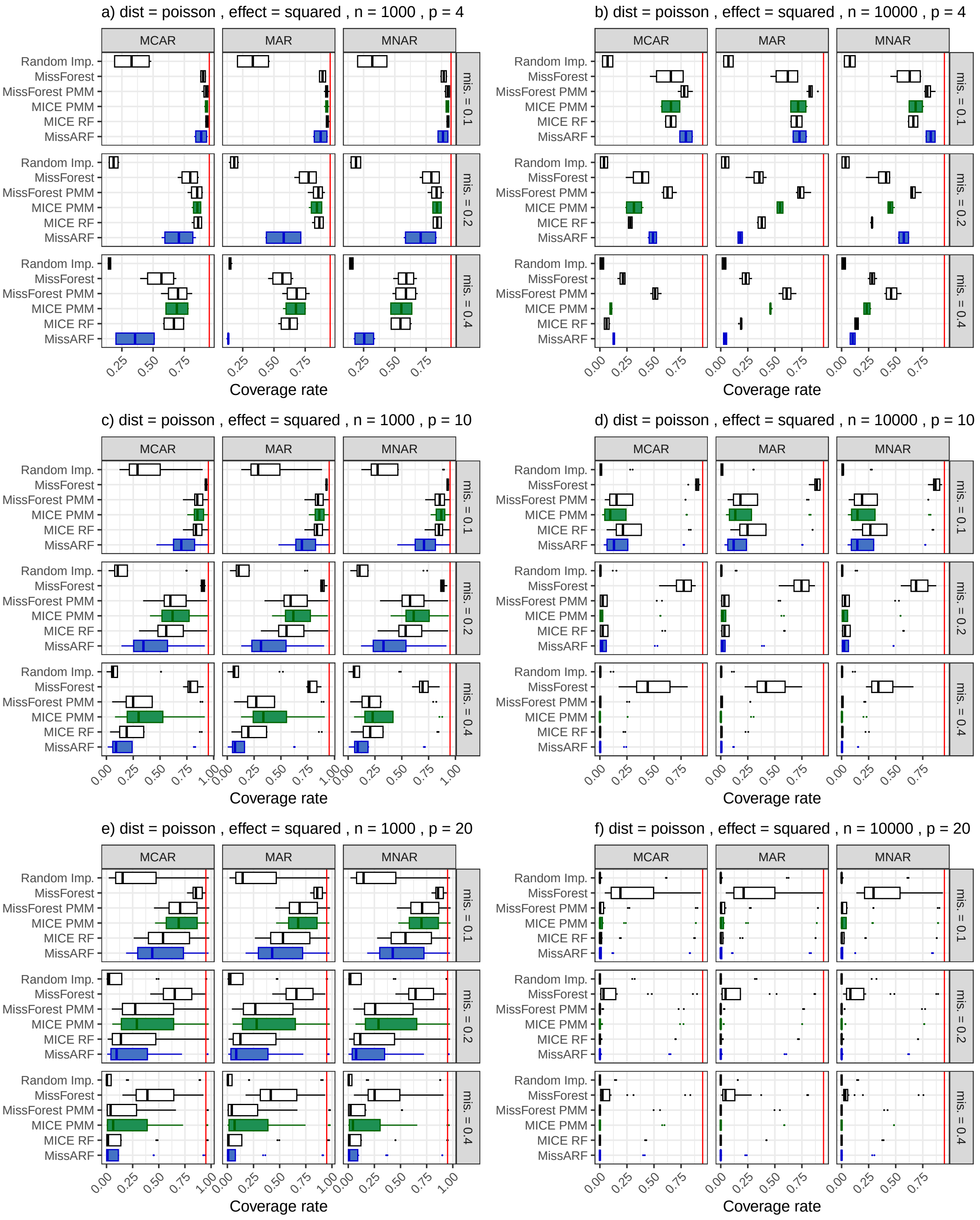

Figure S60: **Coverage rate** of the Poisson distribution setting with a squared effect over different missingness patterns, dimensionality (p) and missingness rates (mis.) with $n = 1000$ (left) and $n = 10,000$ (right). The red vertical line shows the nominal coverage level of 0.95. Boxplots are plotted over features, with MissARF (blue) and MICE PMM (green).

2.2 Average CI Width

2.2.1 Category 1: Similar performance across all methods, MissARF with smallest average width

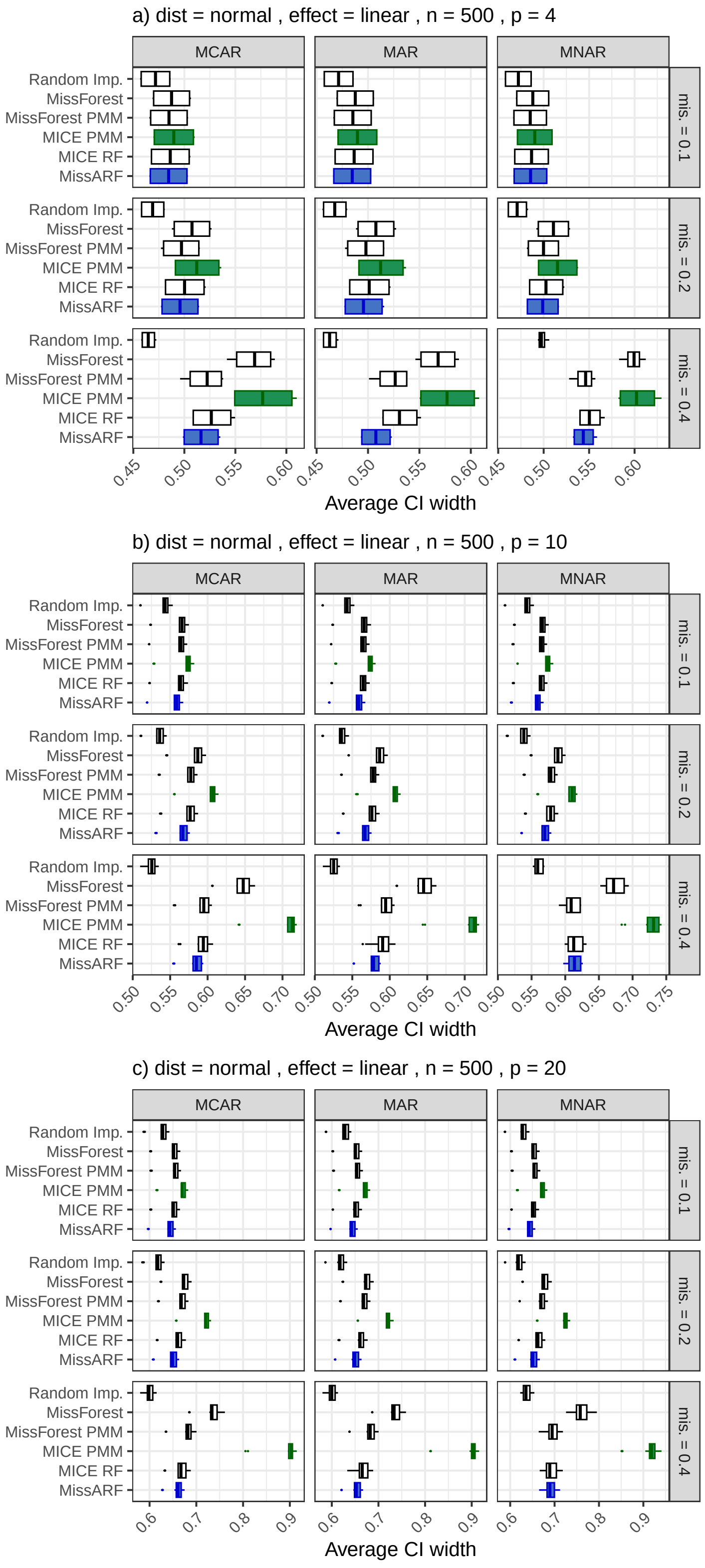

Figure S61: **Average width of the confidence intervals** of the normal distribution setting with a linear effect over different missingness patterns, dimensionality (p) and missingness rates (mis.) with $n = 500$. The boxplots are plotted over the features, with MissARF (blue) and MICE PMM (green) highlighted.

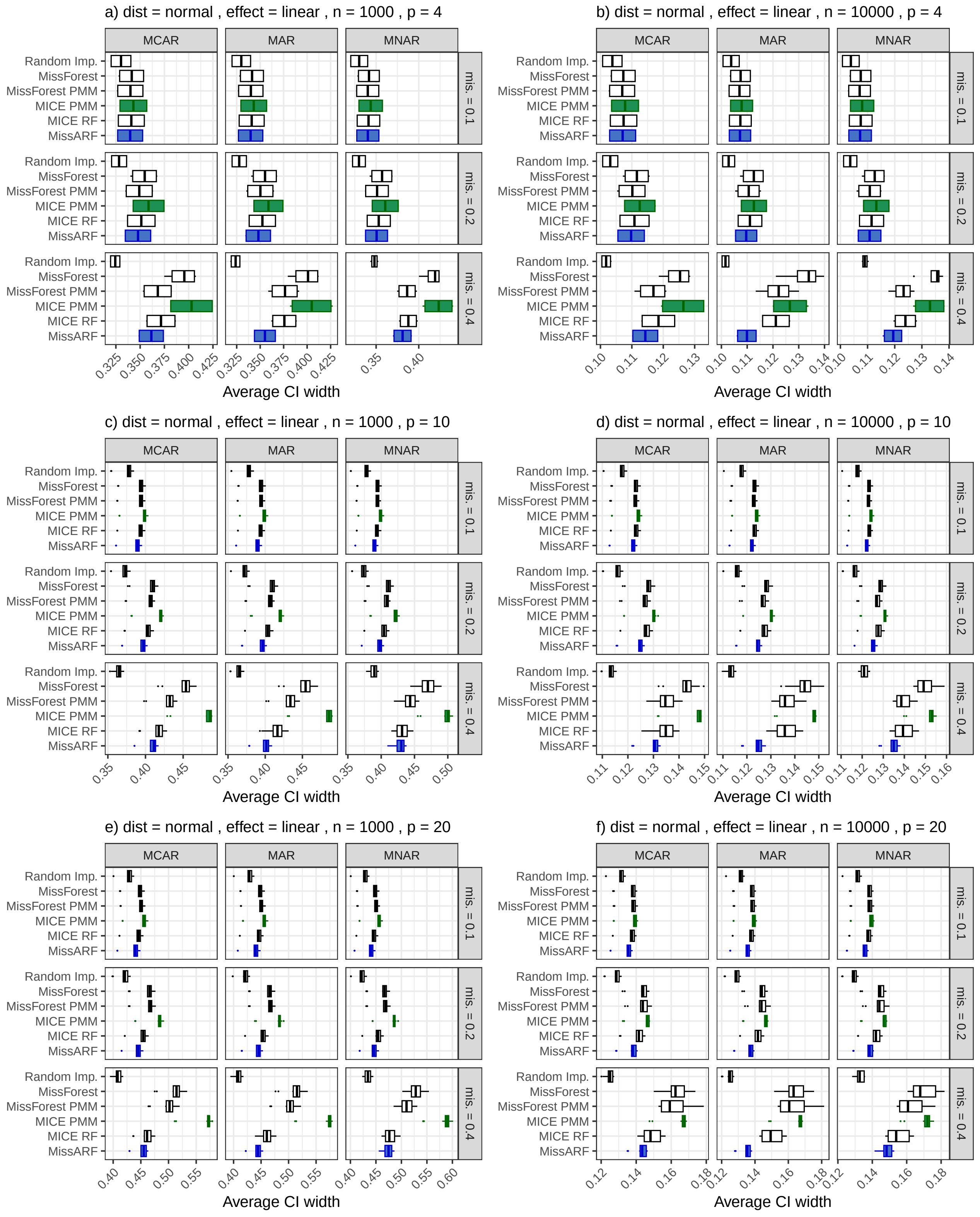

Figure S62: **Average width of the confidence intervals** of the normal distribution setting with a linear effect over different missingness patterns, dimensionality (p) and missingness rates (mis.) with $n = 1000$ (left) and $n = 10,000$ (right). The boxplots are plotted over the features, with MissARF (blue) and MICE PMM (green) highlighted.

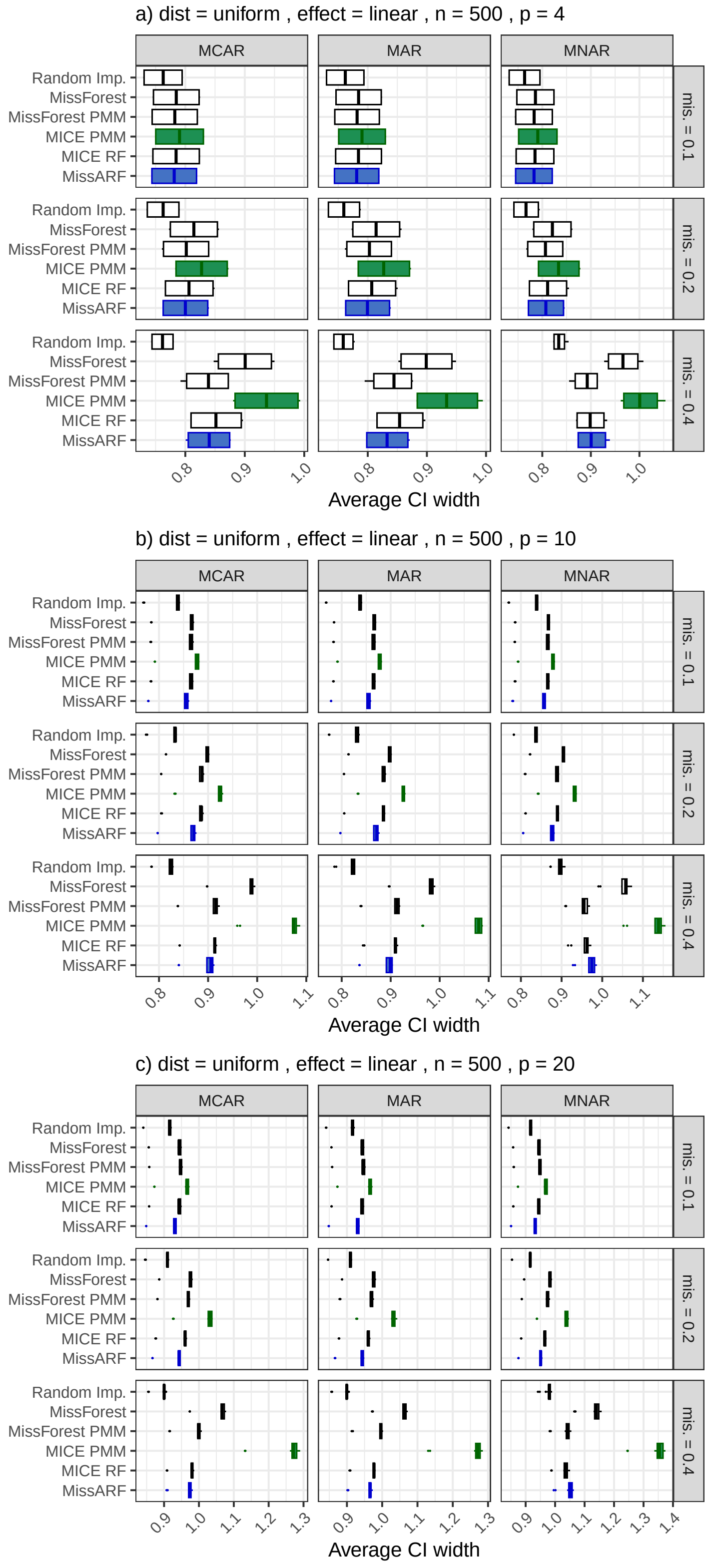

Figure S63: **Average width of the confidence intervals** of the uniform distribution setting with a linear effect over different missingness patterns, dimensionality (p) and missingness rates (mis.) with $n = 500$. The boxplots are plotted over the features, with MissARF (blue) and MICE PMM (green) highlighted.

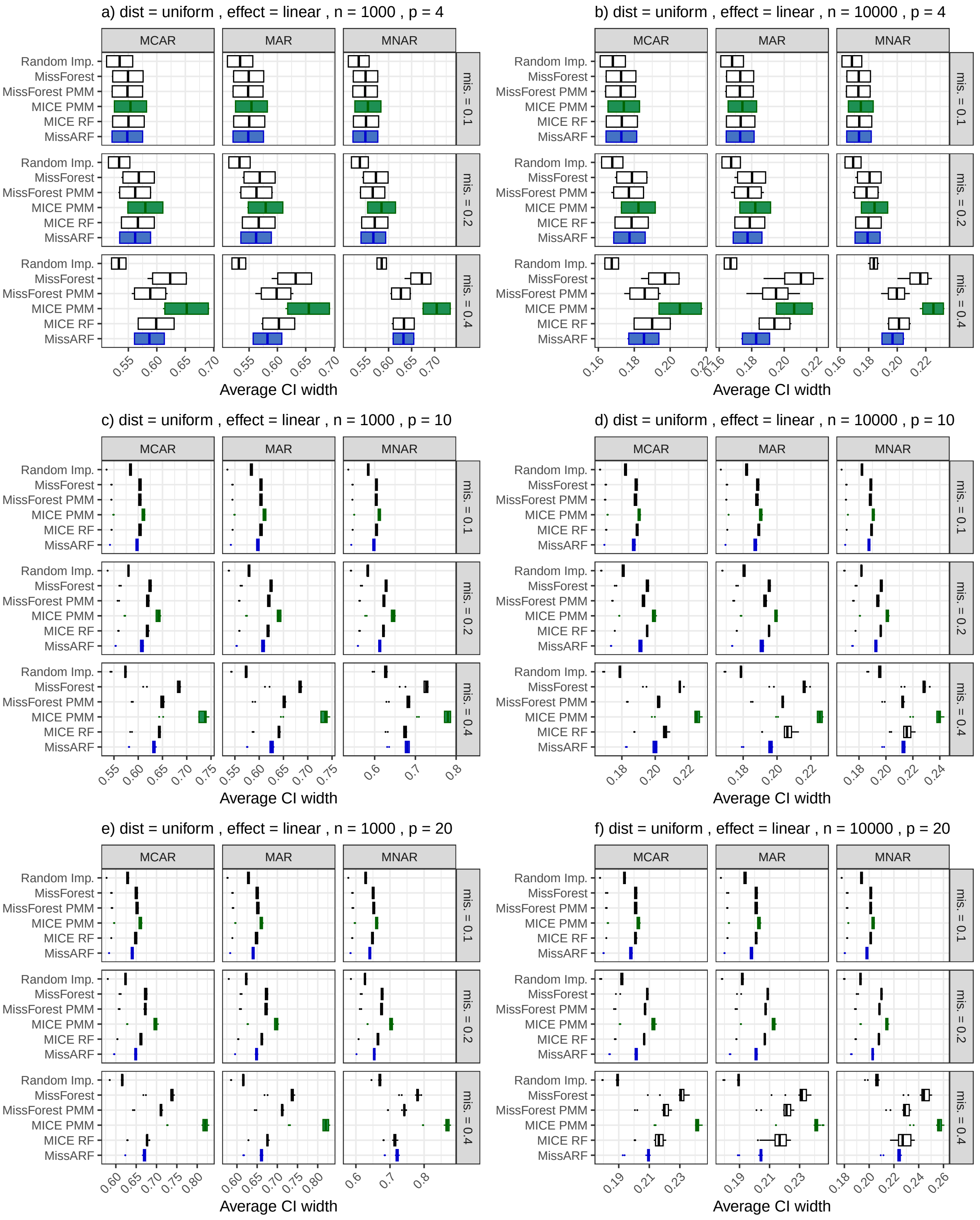

Figure S64: **Average width of the confidence intervals** of the uniform distribution setting with a linear effect over different missingness patterns, dimensionality (p) and missingness rates (mis.) with $n = 1000$ (left) and $n = 10,000$ (right). The boxplots are plotted over the features, with MissARF (blue) and MICE PMM (green) highlighted.

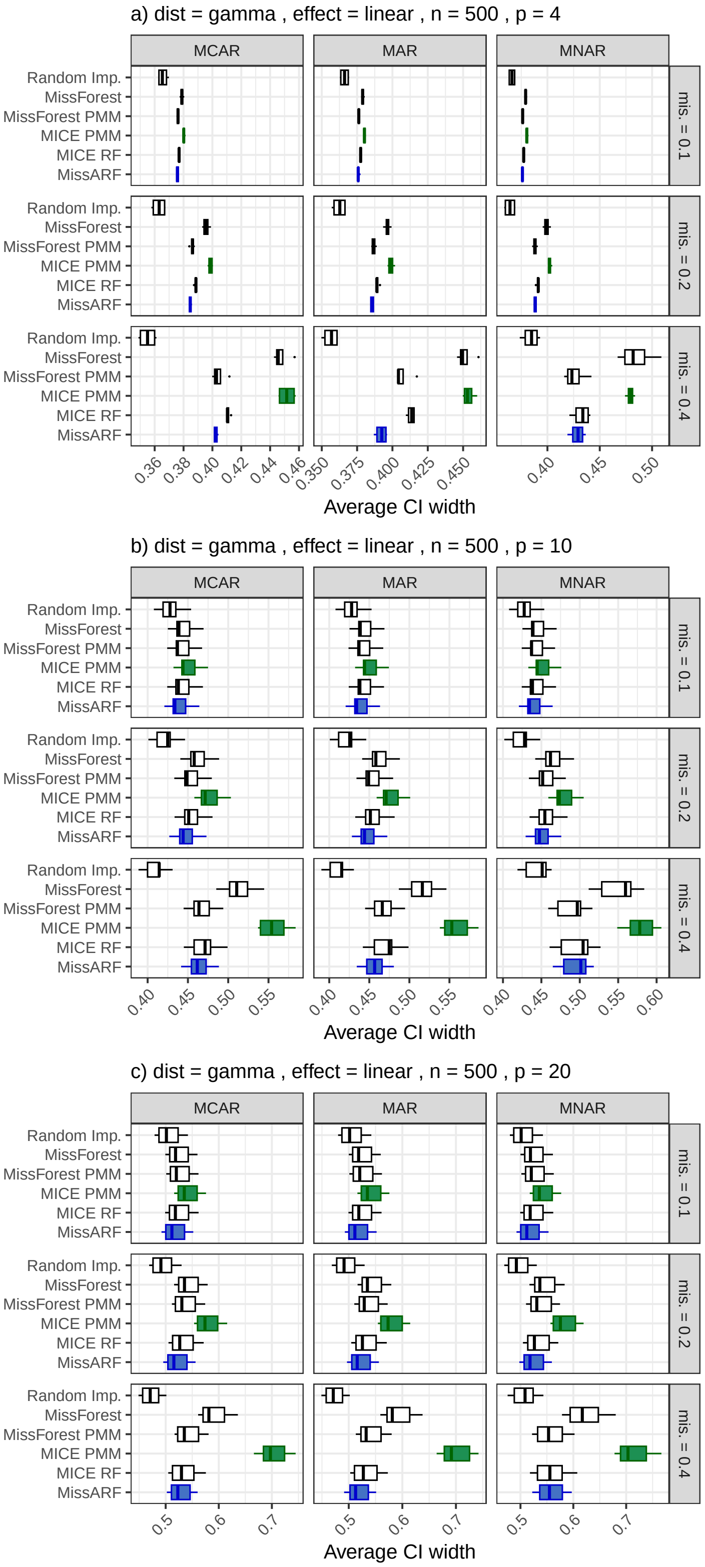

Figure S65: **Average width of the confidence intervals** of the gamma distribution setting with a linear effect over different missingness patterns, dimensionality (p) and missingness rates (mis.) with $n = 500$. The boxplots are plotted over the features, with MissARF (blue) and MICE PMM (green) highlighted.

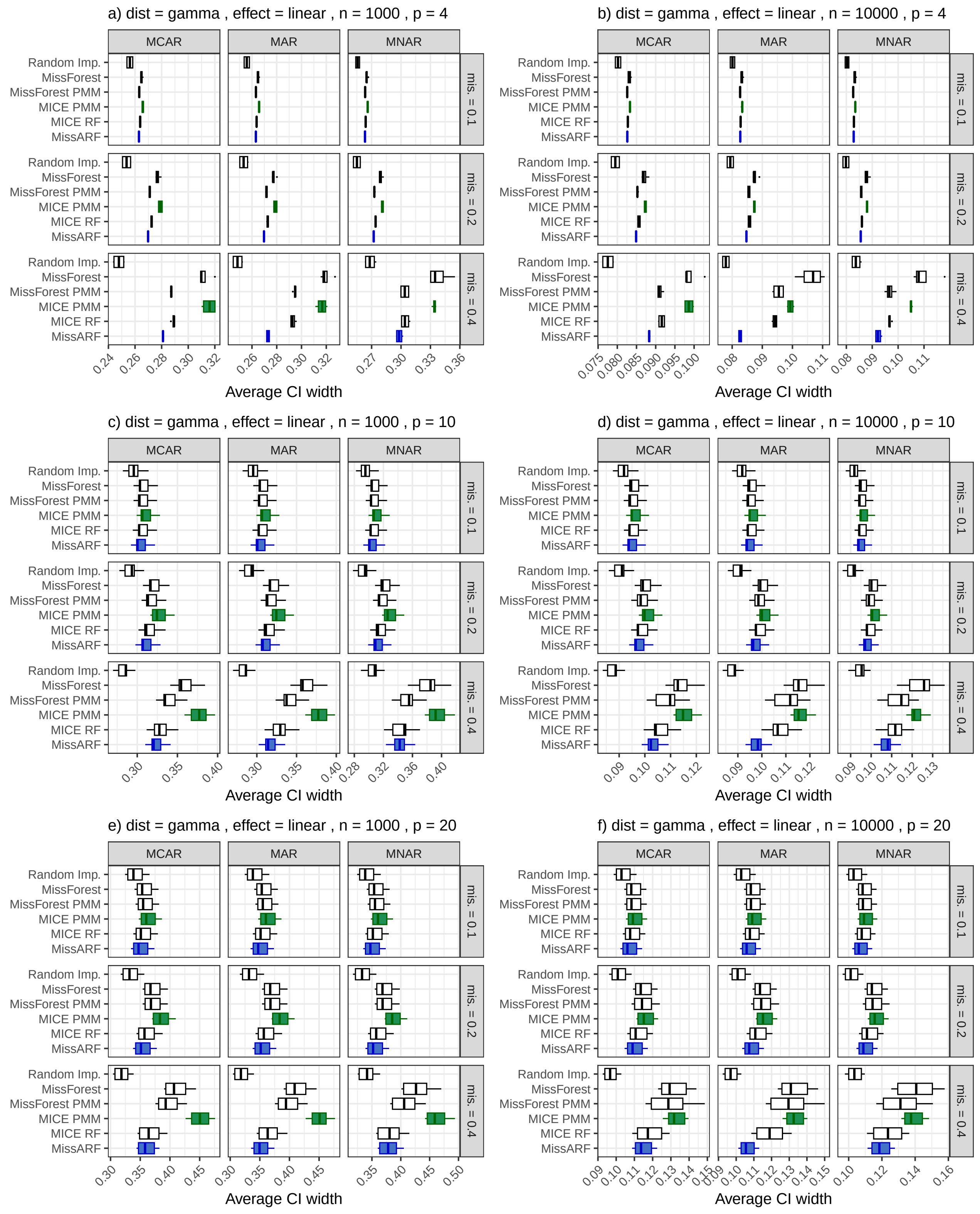

Figure S66: **Average width of the confidence intervals** of the gamma distribution setting with a linear effect over different missingness patterns, dimensionality (p) and missingness rates (mis.) with $n = 1000$ (left) and $n = 10,000$ (right). The boxplots are plotted over the features, with MissARF (blue) and MICE PMM (green) highlighted.

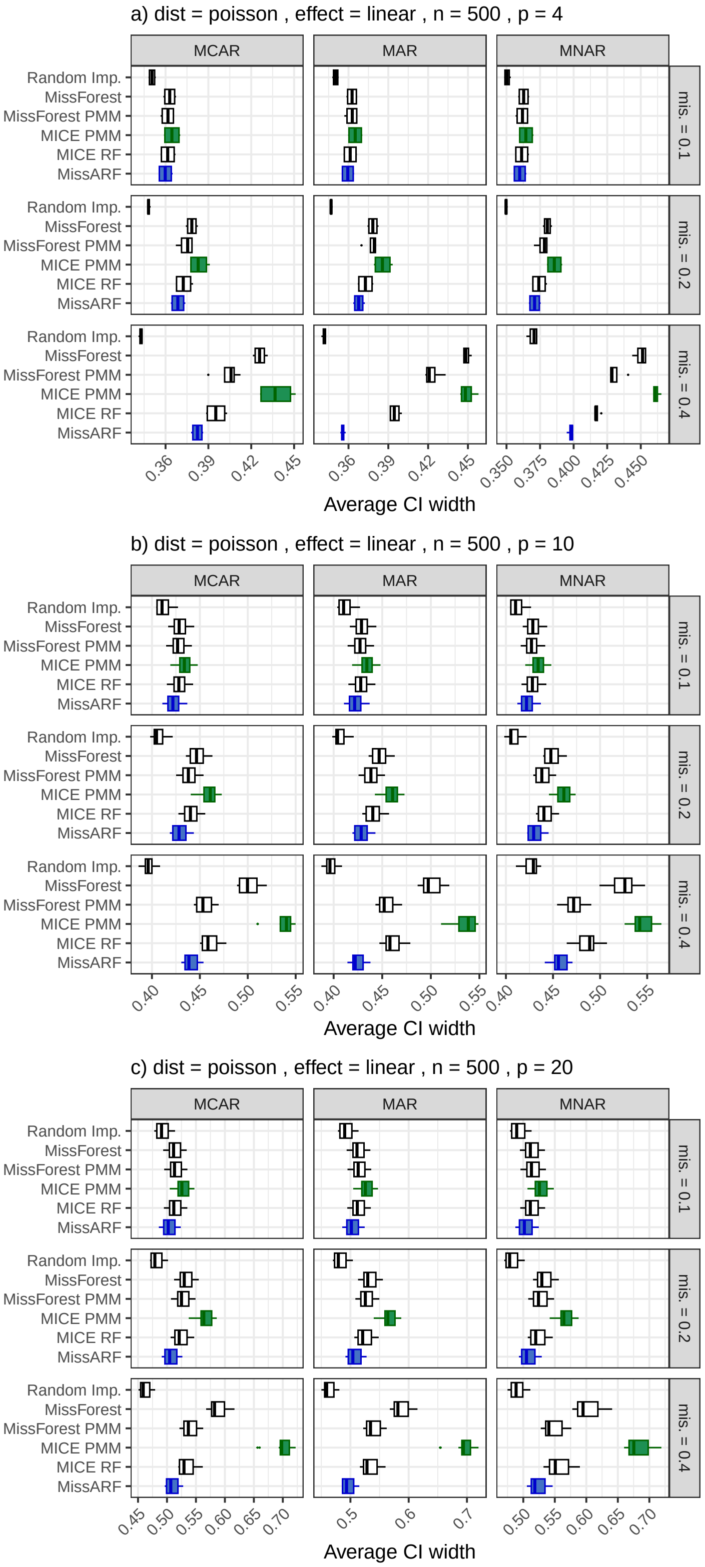

Figure S67: **Average width of the confidence intervals** of the Poisson distribution setting with a linear effect over different missingness patterns, dimensionality (p) and missingness rates (mis.) with $n = 500$. The boxplots are plotted over the features, with MissARF (blue) and MICE PMM (green) highlighted.

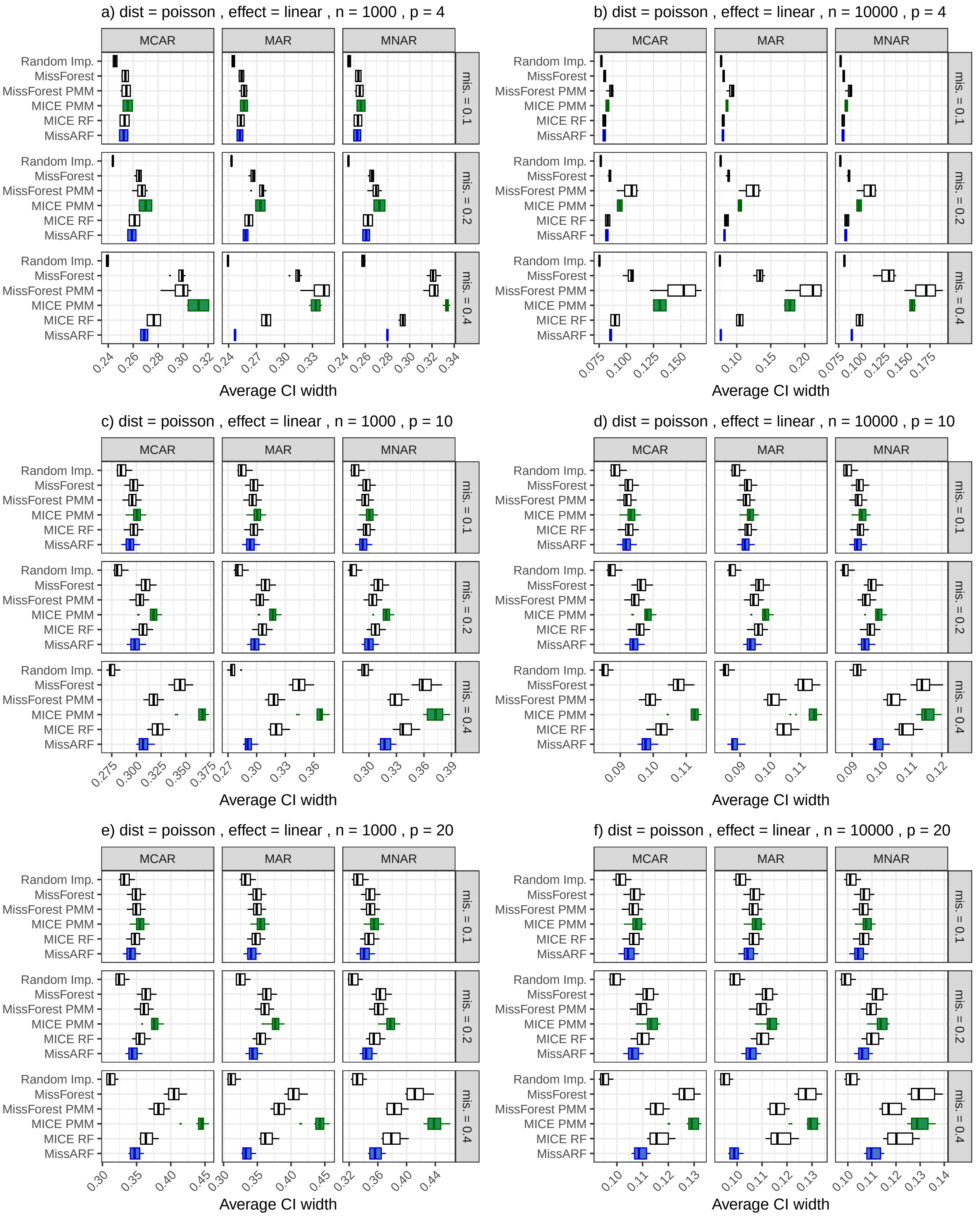

Figure S68: **Average width of the confidence intervals** of the Poisson distribution setting with a linear effect over different missingness patterns, dimensionality (p) and missingness rates (mis.) with $n = 1000$ (left) and $n = 10,000$ (right). The boxplots are plotted over the features, with MissARF (blue) and MICE PMM (green) highlighted.

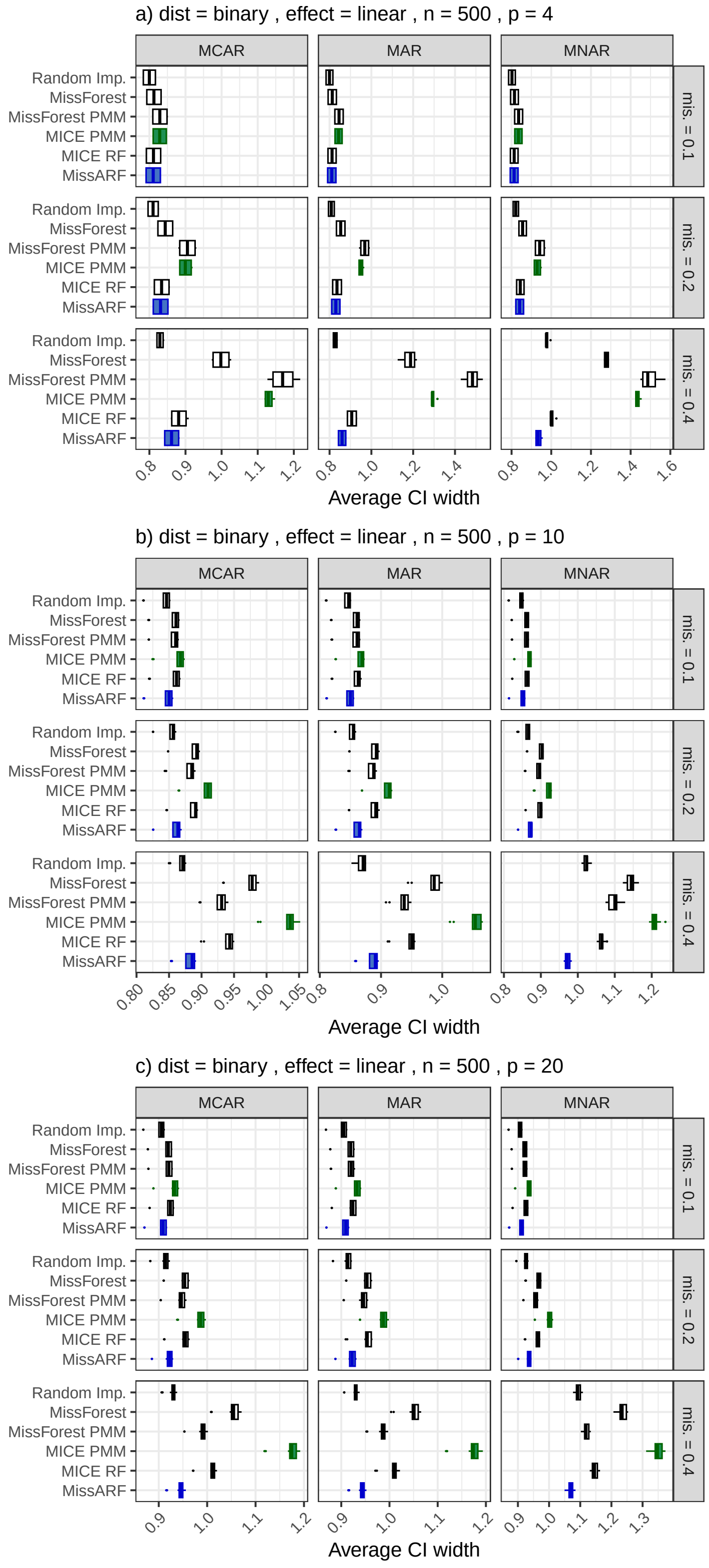

Figure S69: **Average width of the confidence intervals** of the binary distribution setting with a linear effect over different missingness patterns, dimensionality (p) and missingness rates (mis.) with $n = 500$. The boxplots are plotted over the features, with MissARF (blue) and MICE PMM (green) highlighted.

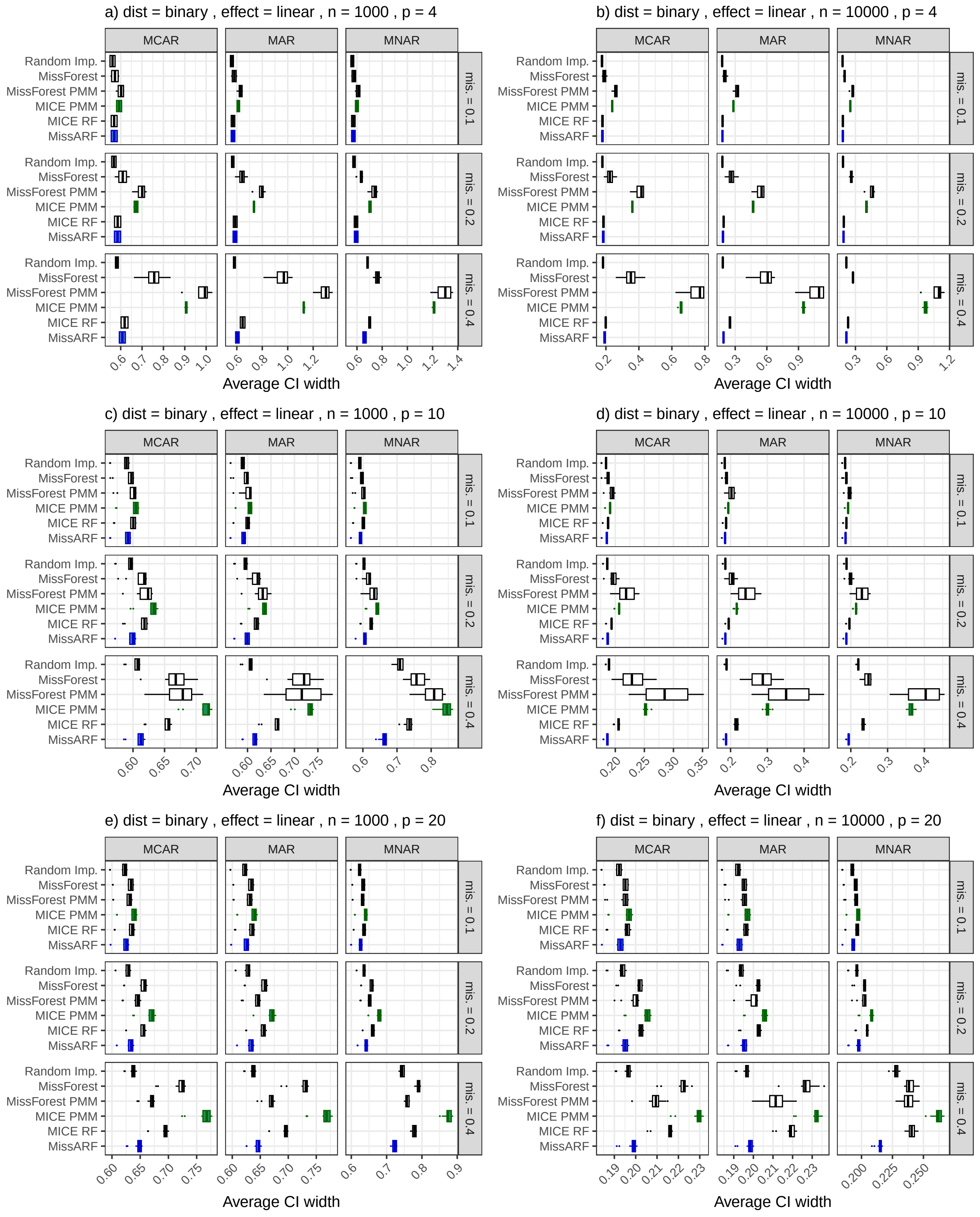

Figure S70: **Average width of the confidence intervals** of the binary distribution setting with a linear effect over different missingness patterns, dimensionality (p) and missingness rates (mis.) with $n = 1000$ (left) and $n = 10,000$ (right). The boxplots are plotted over the features, with MissARF (blue) and MICE PMM (green) highlighted.

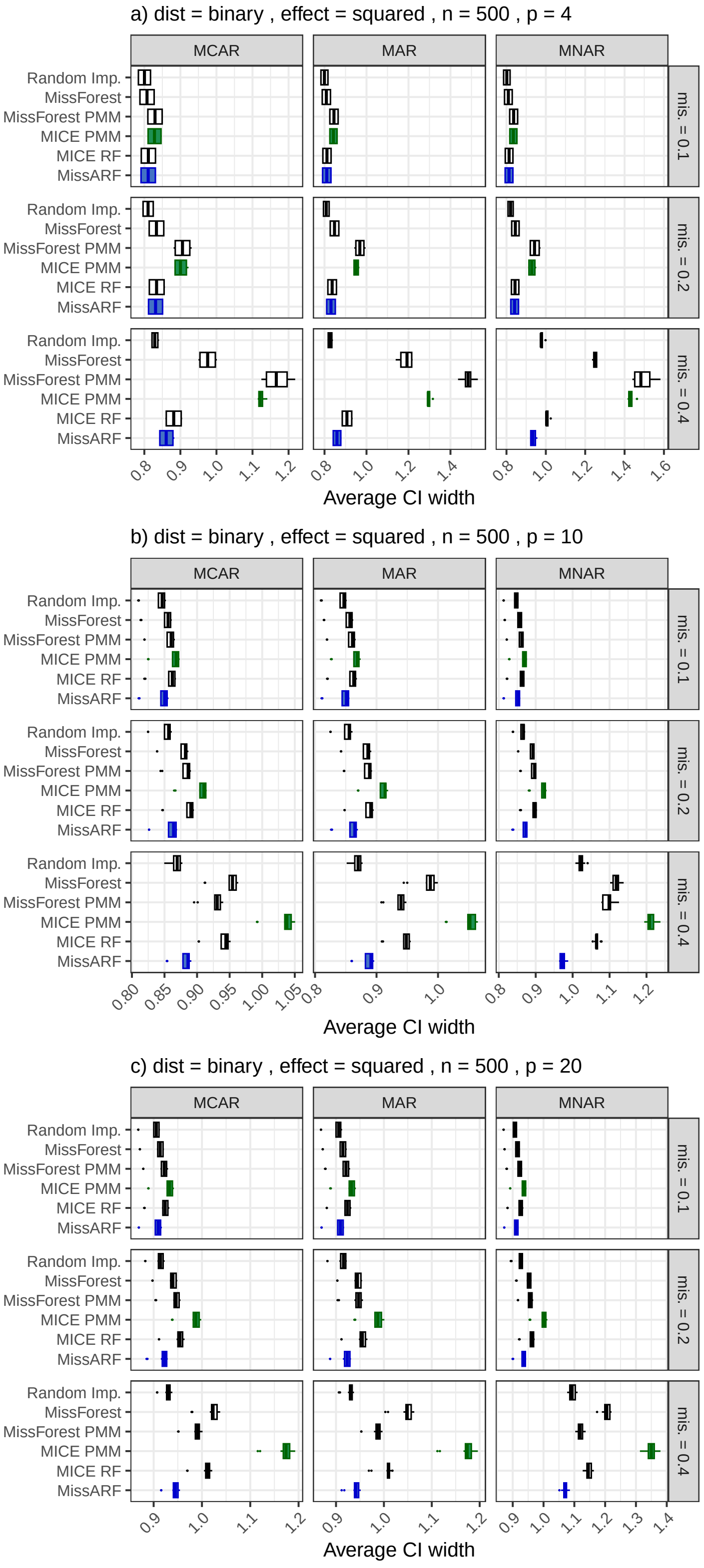

Figure S71: **Average width of the confidence intervals** of the binary distribution setting with a squared effect over different missingness patterns, dimensionality (p) and missingness rates (mis.) with $n = 500$. The boxplots are plotted over the features, with MissARF (blue) and MICE PMM (green) highlighted.

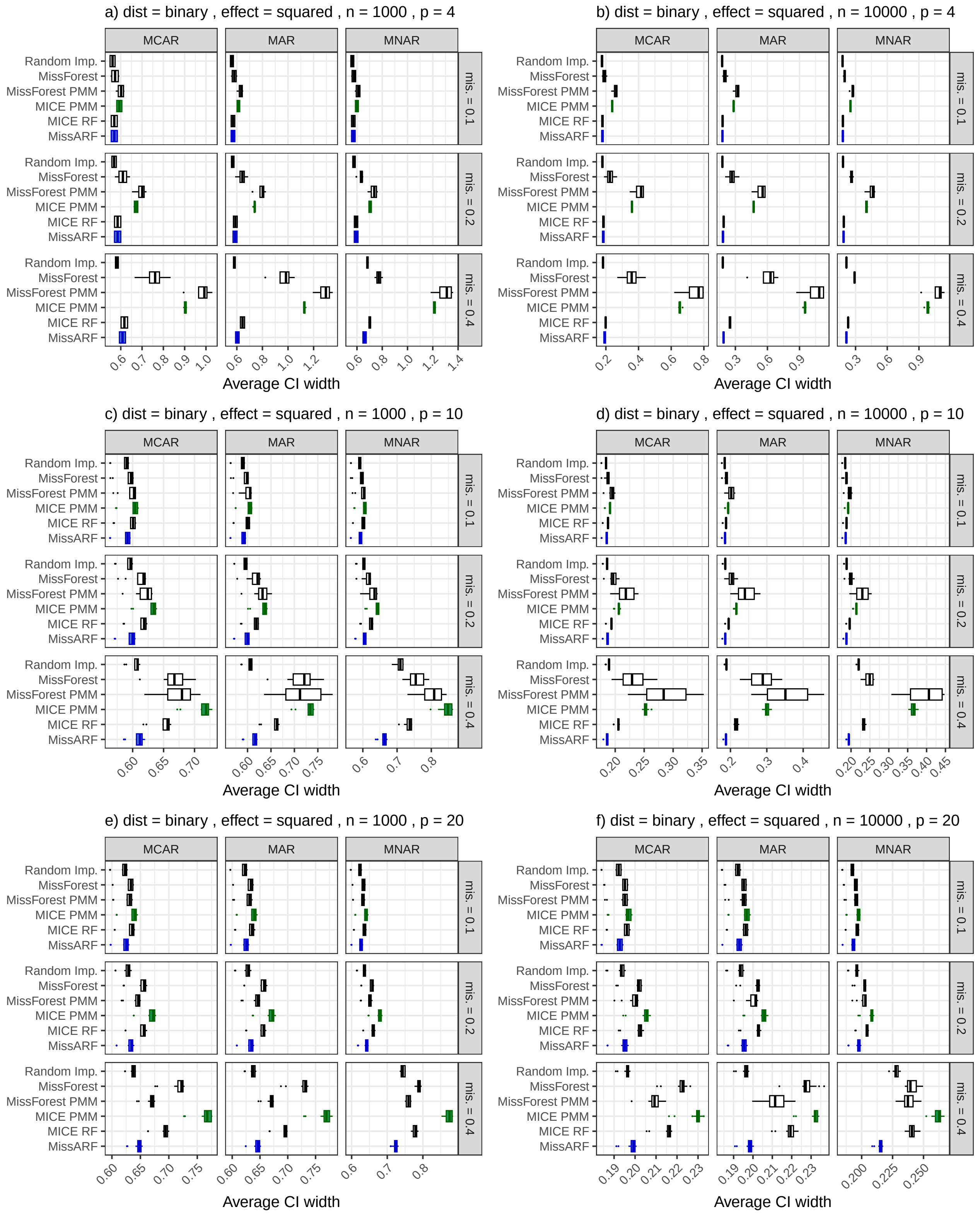

Figure S72: **Average width of the confidence intervals** of the binary distribution setting with a squared effect over different missingness patterns, dimensionality (p) and missingness rates (mis.) with $n = 1000$ (left) and $n = 10,000$ (right). The boxplots are plotted over the features, with MissARF (blue) and MICE PMM (green) highlighted.

2.2.2 Category 2: PMM methods struggle, MissARF performs well

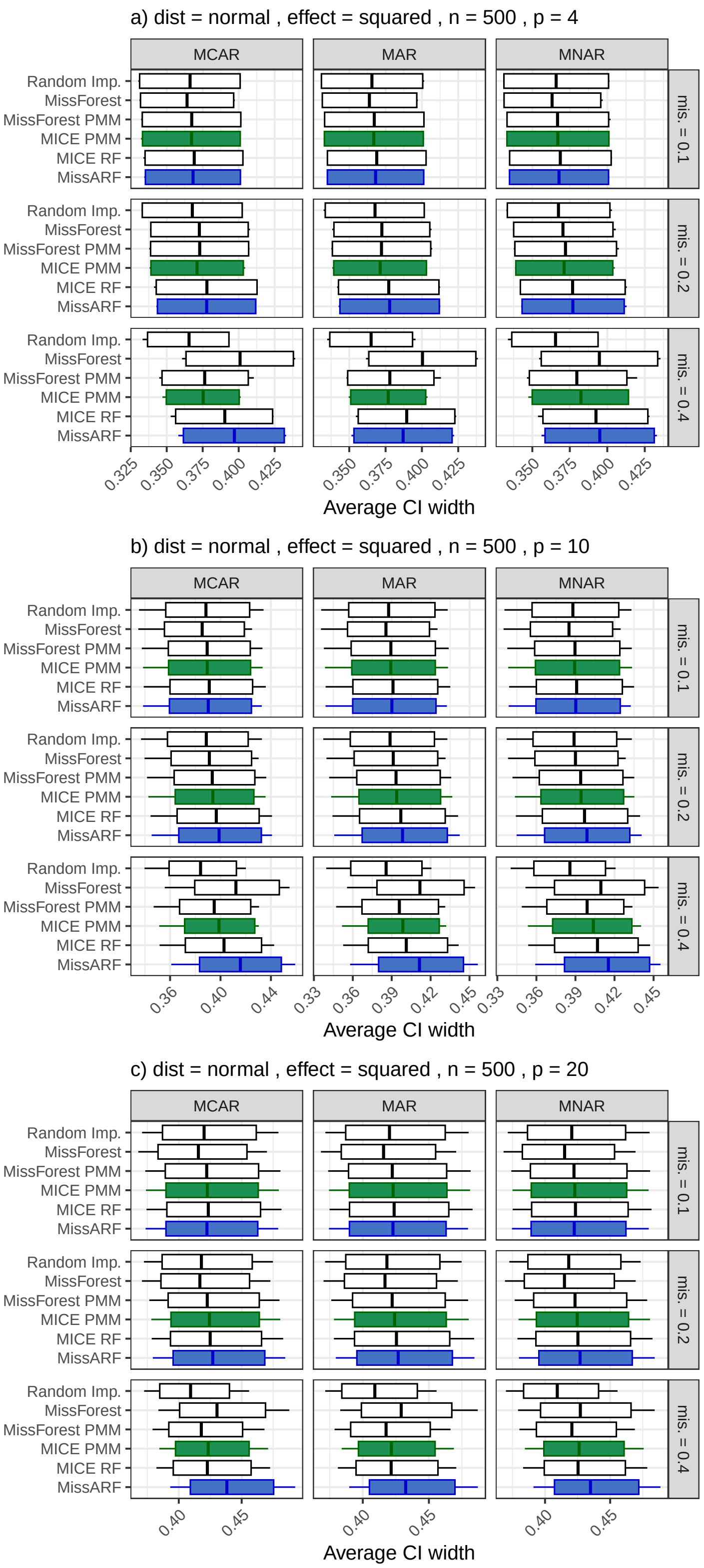

Figure S73: **Average width of the confidence intervals** of the normal distribution setting with a squared effect over different missingness patterns, dimensionality (p) and missingness rates (mis.) with $n = 500$. The boxplots are plotted over the features, with MissARF (blue) and MICE PMM (green) highlighted.

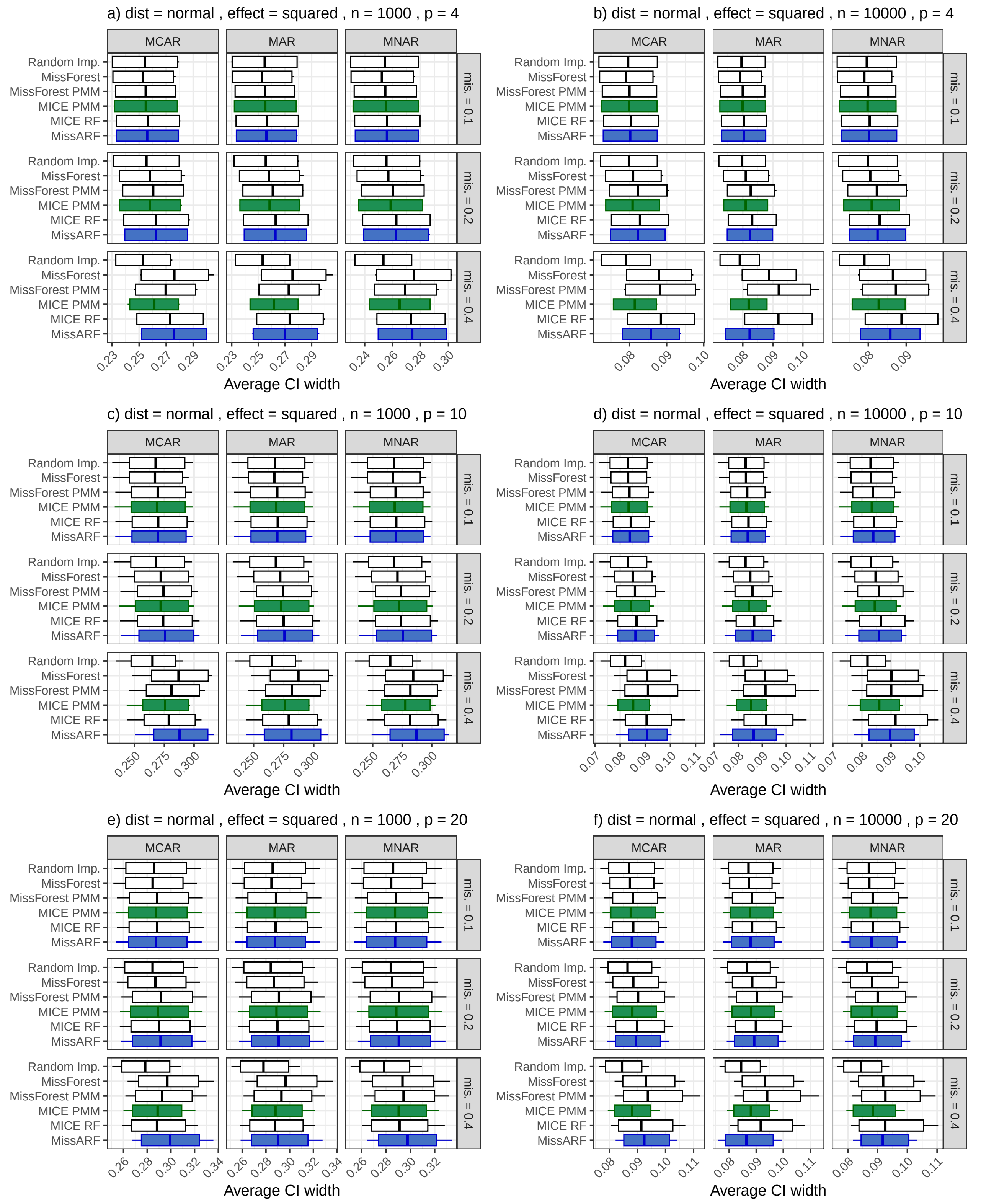

Figure S74: **Average width of the confidence intervals** of the normal distribution setting with a squared effect over different missingness patterns, dimensionality (p) and missingness rates (mis.) with $n = 1000$ (left) and $n = 10,000$ (right). The boxplots are plotted over the features, with MissARF (blue) and MICE PMM (green) highlighted.

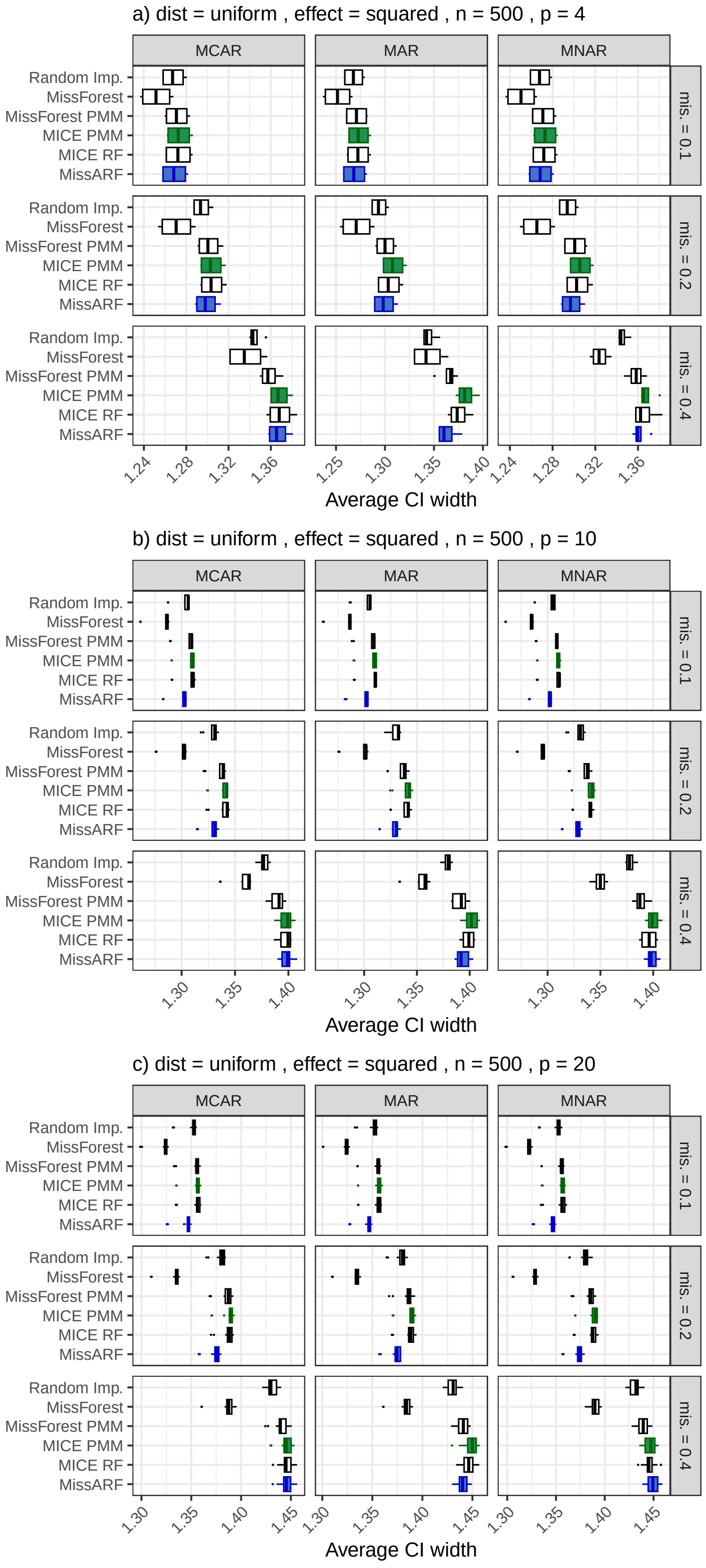

Figure S75: **Average width of the confidence intervals** of the uniform distribution setting with a squared effect over different missingness patterns, dimensionality (p) and missingness rates (mis.) with $n = 500$. The boxplots are plotted over the features, with MissARF (blue) and MICE PMM (green) highlighted.

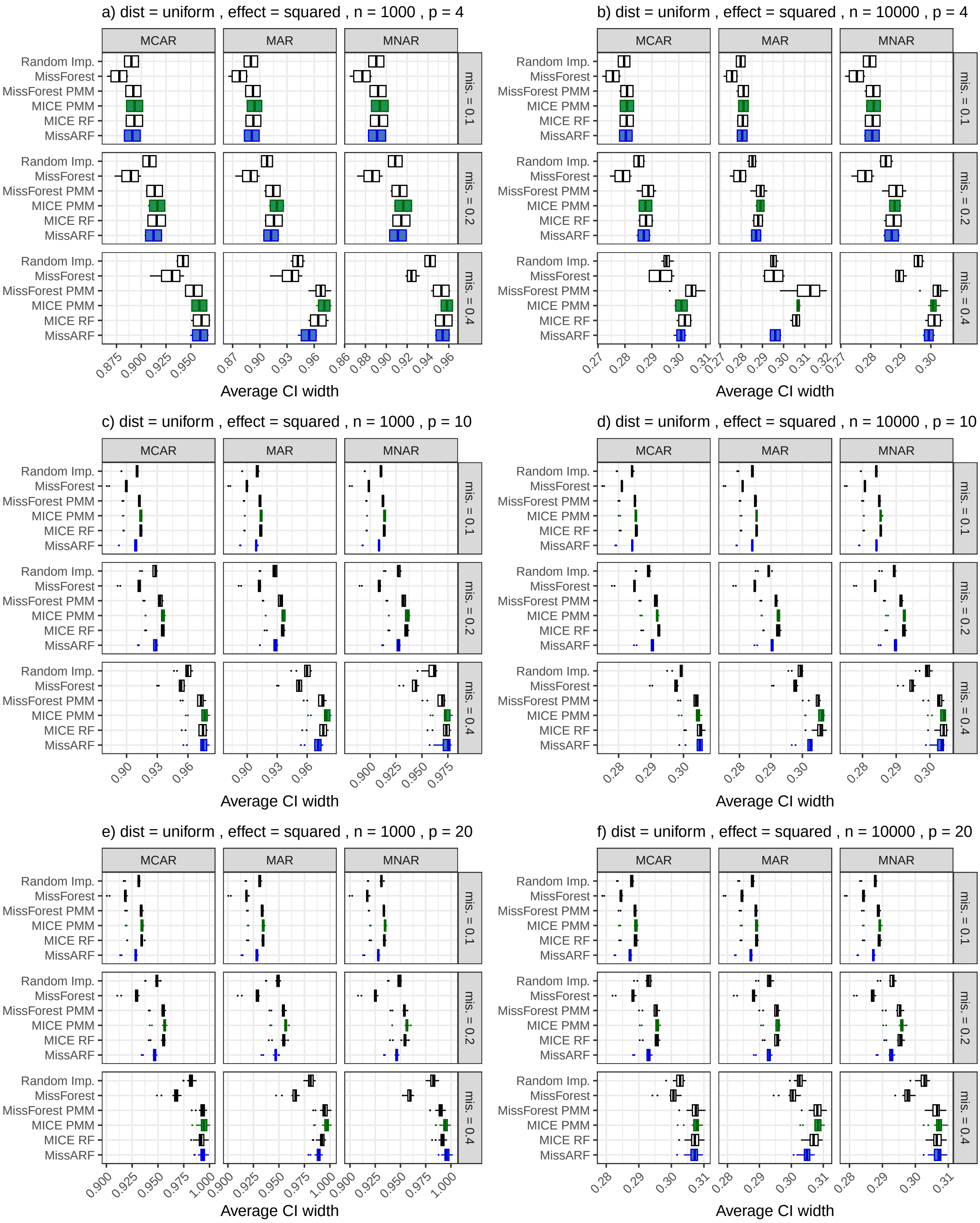

Figure S76: **Average width of the confidence intervals** of the uniform distribution setting with a squared effect over different missingness patterns, dimensionality (p) and missingness rates (mis.) with $n = 1000$ (left) and $n = 10,000$ (right). The boxplots are plotted over the features, with MissARF (blue) and MICE PMM (green) highlighted.

2.2.3 Category 3: All methods perform poorly

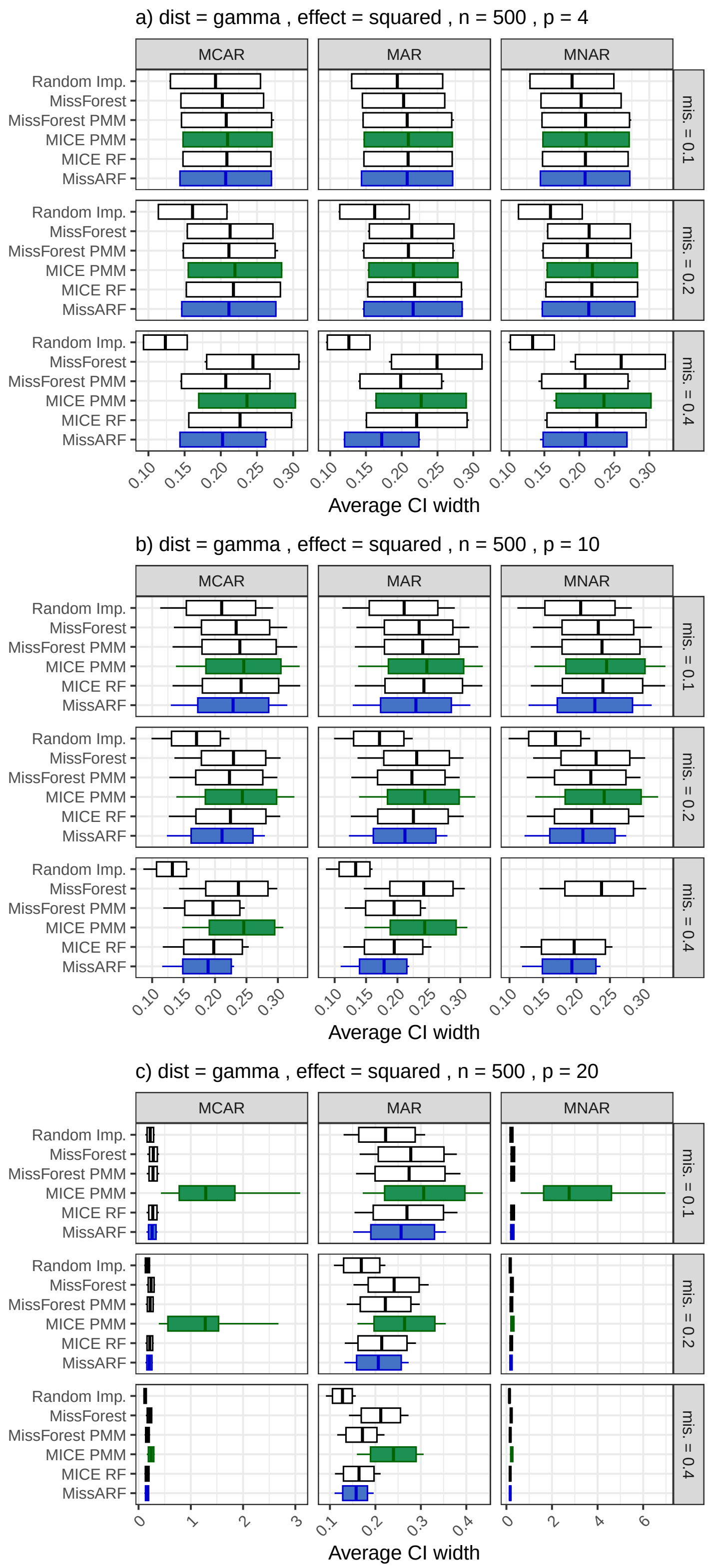

Figure S77: **Average width of the confidence intervals** of the gamma distribution setting with a squared effect over different missingness patterns, dimensionality (p) and missingness rates (mis.) with $n = 500$. The boxplots are plotted over the features, with MissARF (blue) and MICE PMM (green) highlighted.

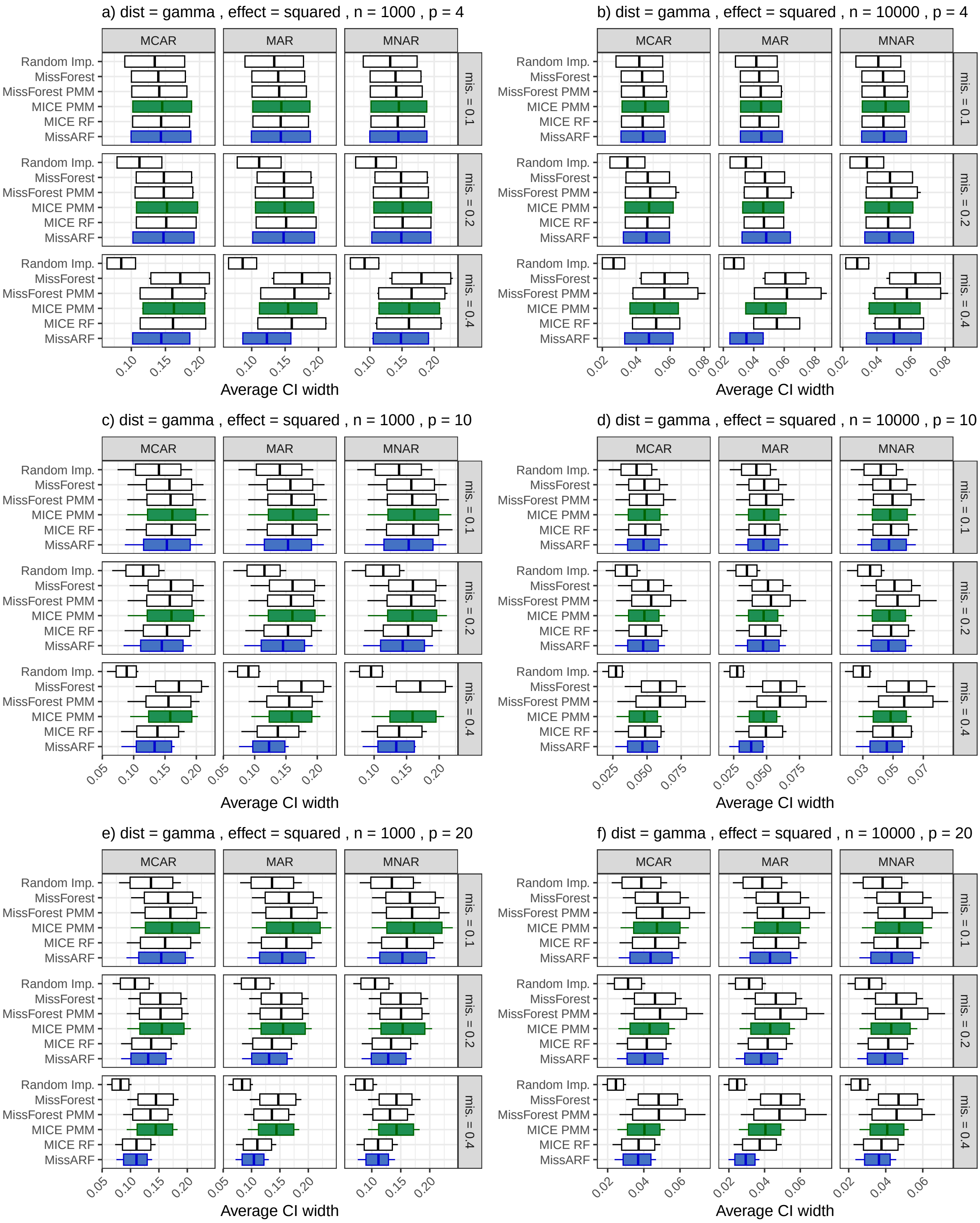

Figure S78: **Average width of the confidence intervals** of the gamma distribution setting with a squared effect over different missingness patterns, dimensionality (p) and missingness rates (mis.) with $n = 1000$ (left) and $n = 10,000$ (right). The boxplots are plotted over the features, with MissARF (blue) and MICE PMM (green) highlighted.

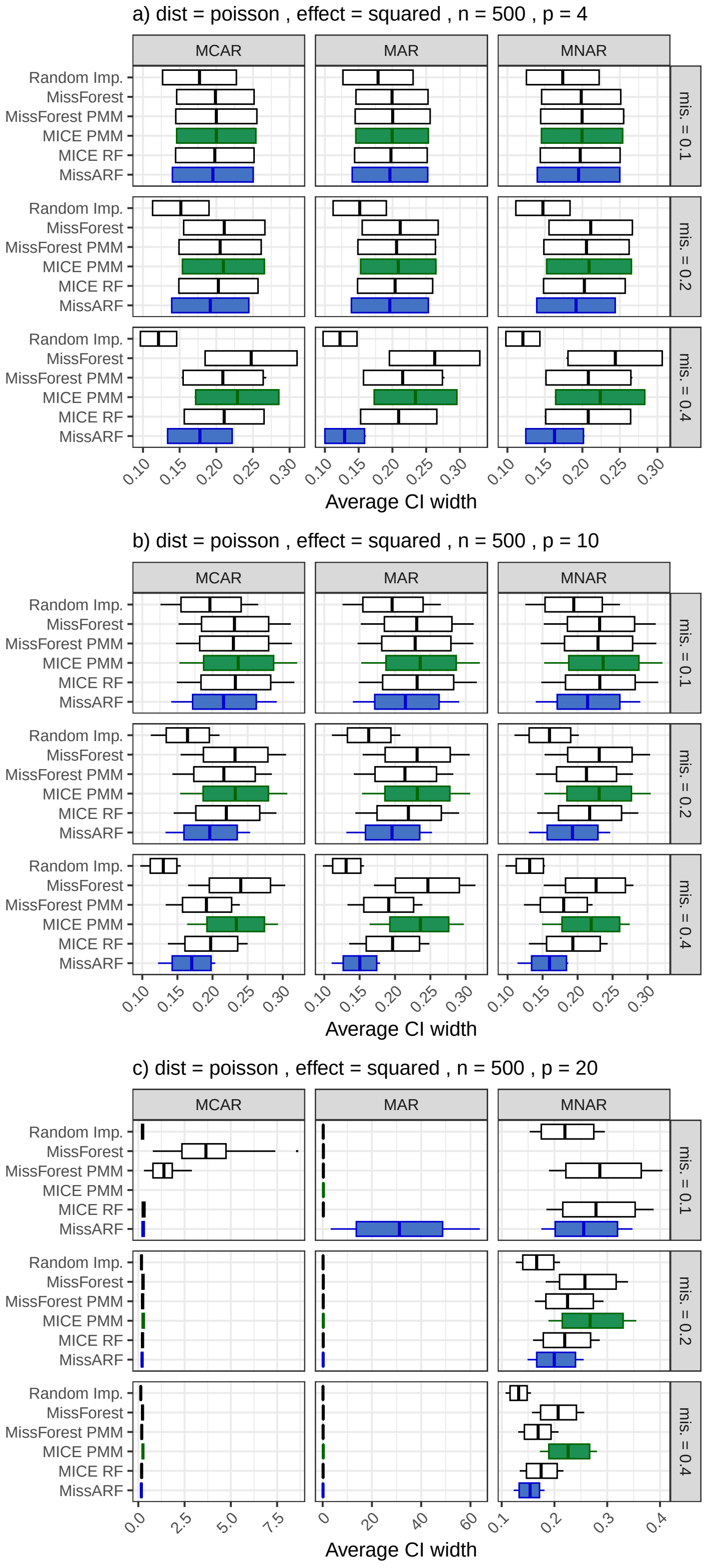

Figure S79: **Average width of the confidence intervals** of the Poisson distribution setting with a squared effect over different missingness patterns, dimensionality (p) and missingness rates (mis.) with $n = 500$. The boxplots are plotted over the features, with MissARF (blue) and MICE PMM (green) highlighted.

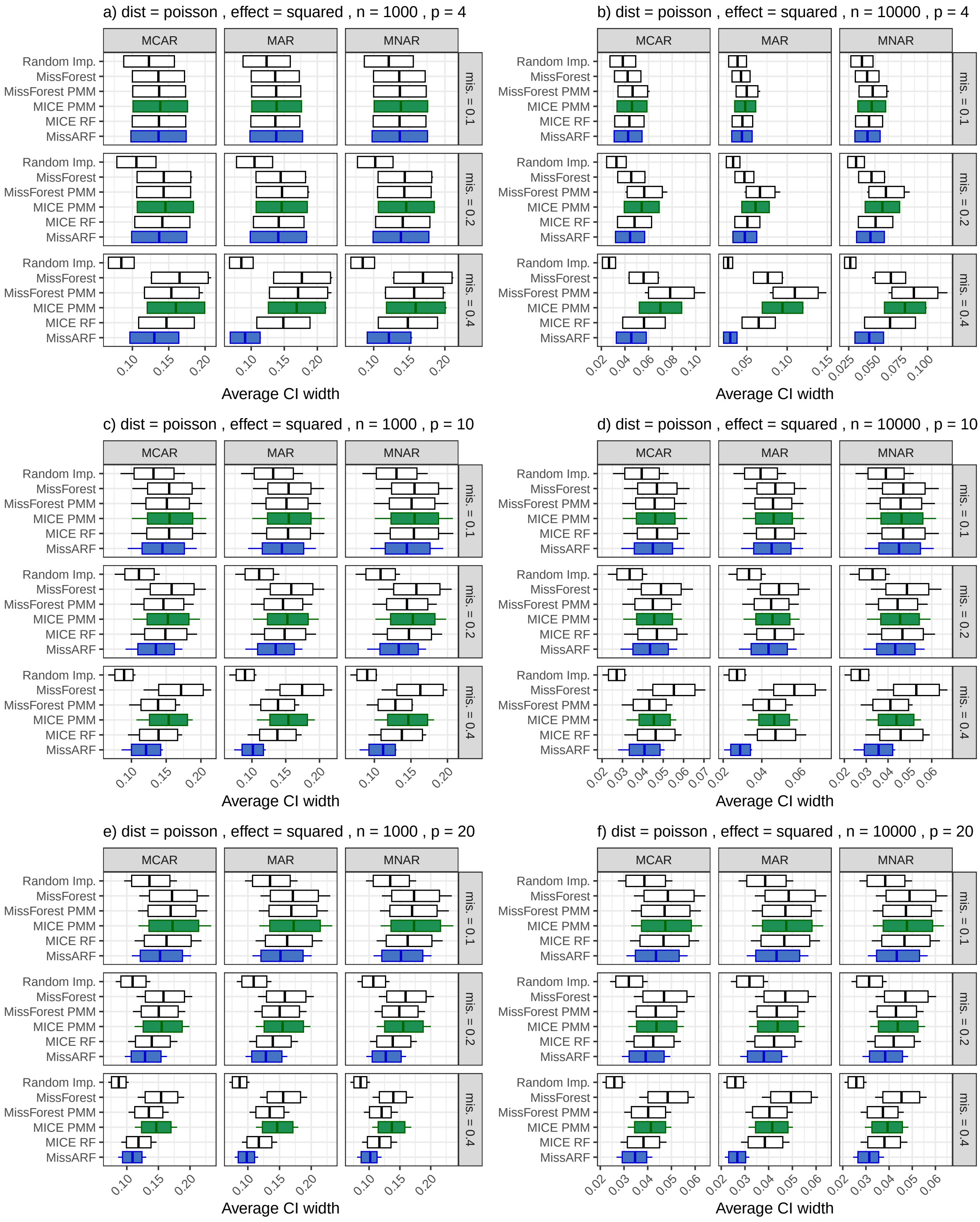

Figure S80: **Average width of the confidence intervals** of the Poisson distribution setting with a squared effect over different missingness patterns, dimensionality (p) and missingness rates (mis.) with $n = 1000$ (left) and $n = 10,000$ (right). The boxplots are plotted over the features, with MissARF (blue) and MICE PMM (green) highlighted.

2.3 RMSE

2.3.1 Category 1: Similar performance across all methods, MissARF with smallest average width

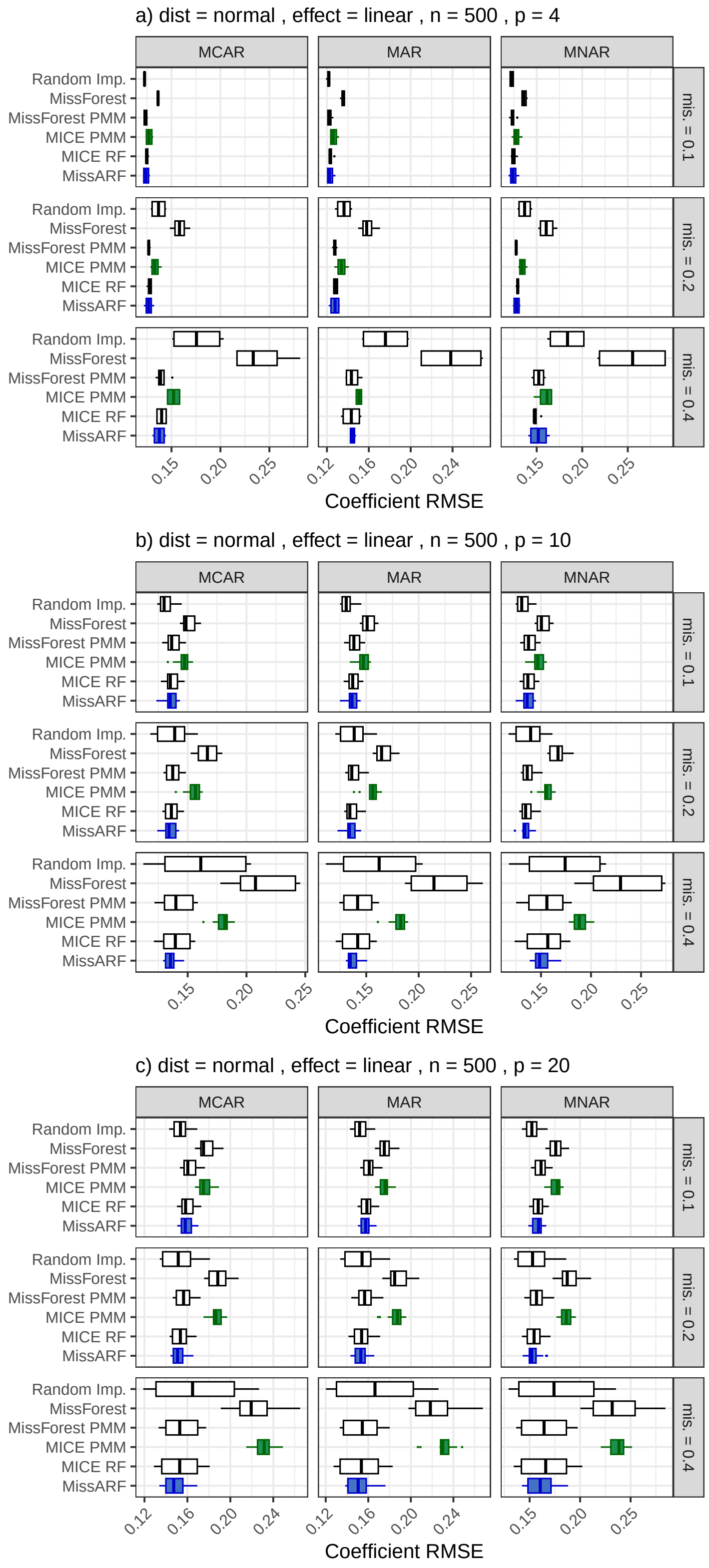

Figure S81: **RMSE of the regression coefficients** of the normal distribution setting with a linear effect over different missingness patterns, dimensionality (p) and missingness rates (mis.) with $n = 500$. The boxplots are plotted over the features, with MissARF (blue) and MICE PMM (green) highlighted.

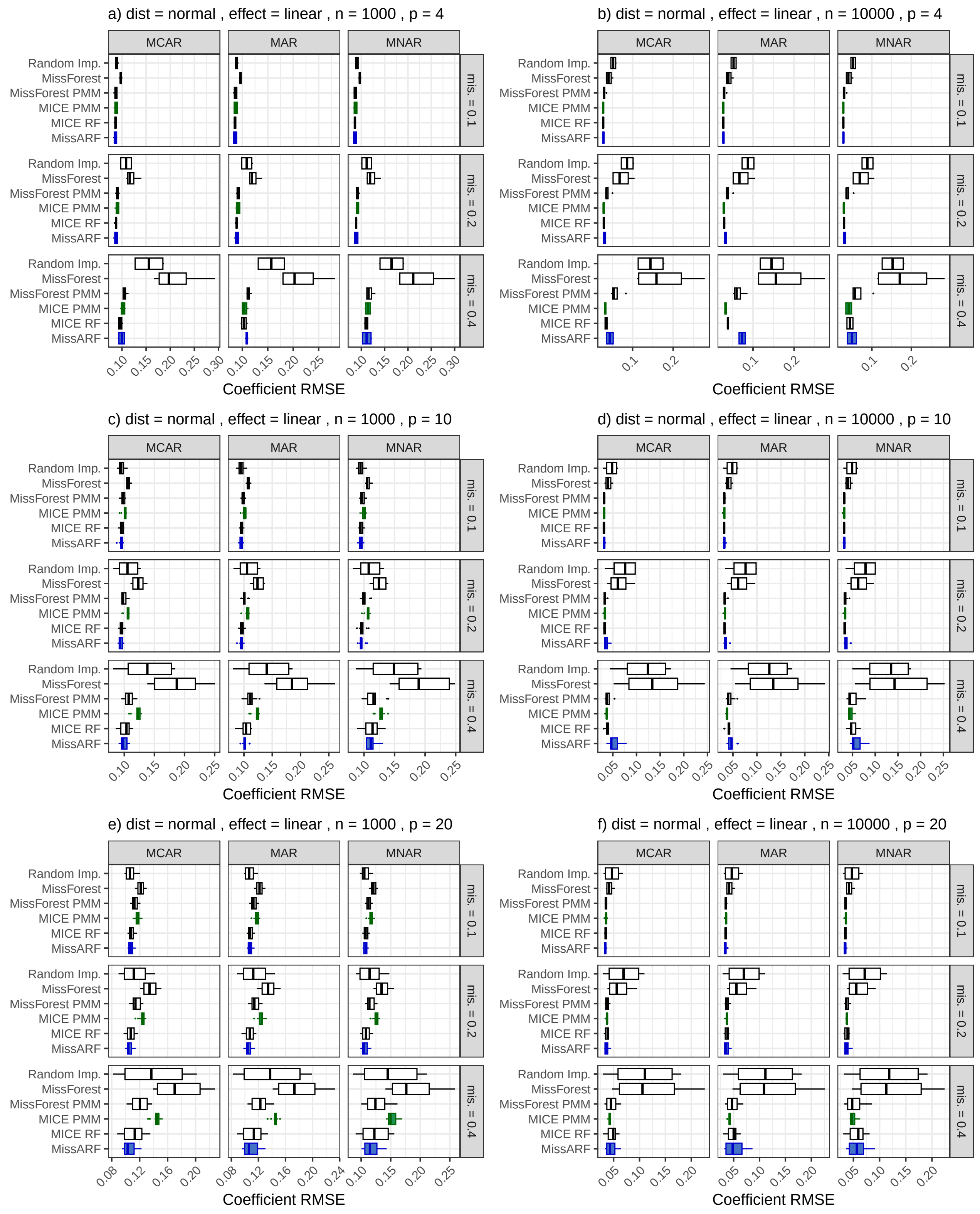

Figure S82: **RMSE of the regression coefficients** of the normal distribution setting with a linear effect over different missingness patterns, dimensionality (p) and missingness rates (mis.) with $n = 1000$ (left) and $n = 10,000$ (right). The boxplots are plotted over the features, with MissARF (blue) and MICE PMM (green) highlighted.

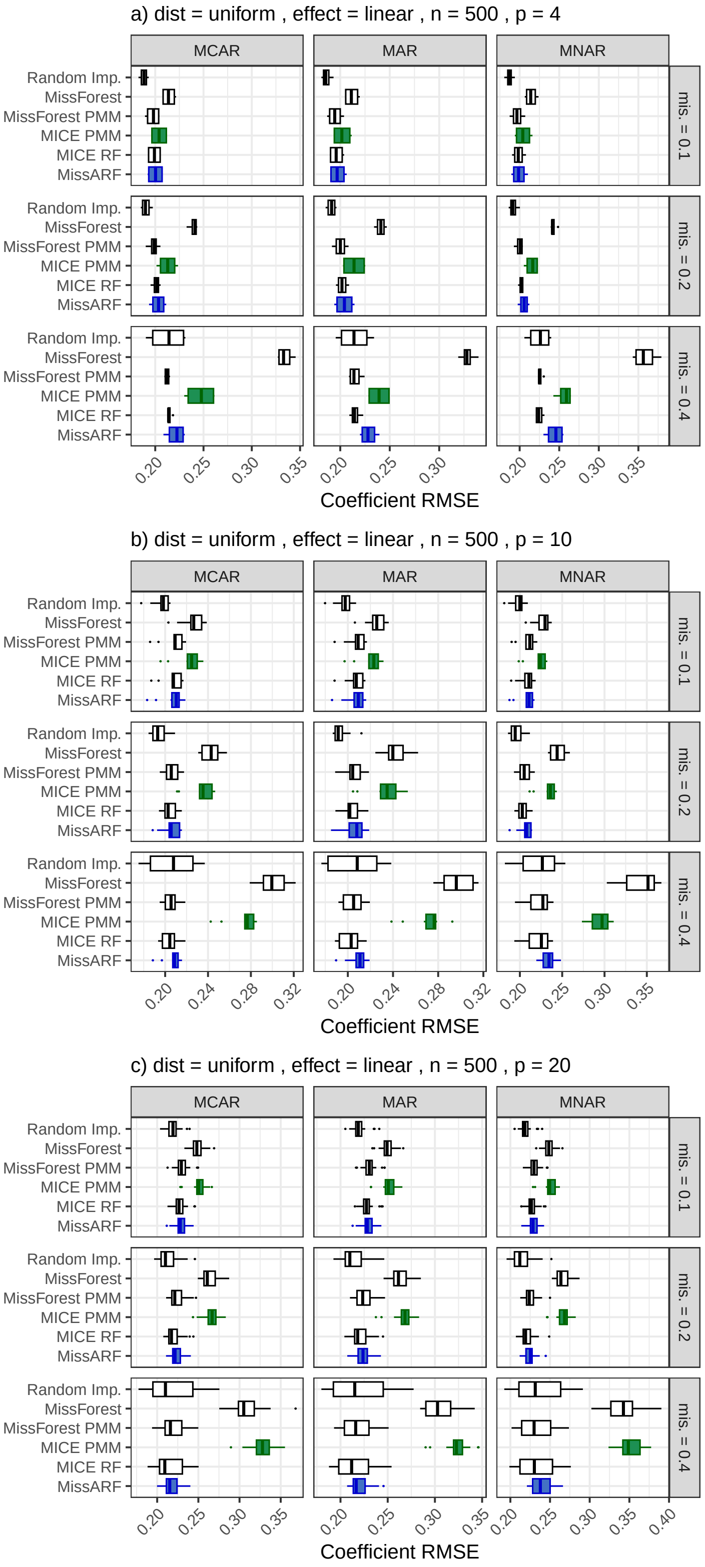

Figure S83: **RMSE of the regression coefficients** of the uniform distribution setting with a linear effect over different missingness patterns, dimensionality (p) and missingness rates (mis.) with $n = 500$. The boxplots are plotted over the features, with MissARF (blue) and MICE PMM (green) highlighted.

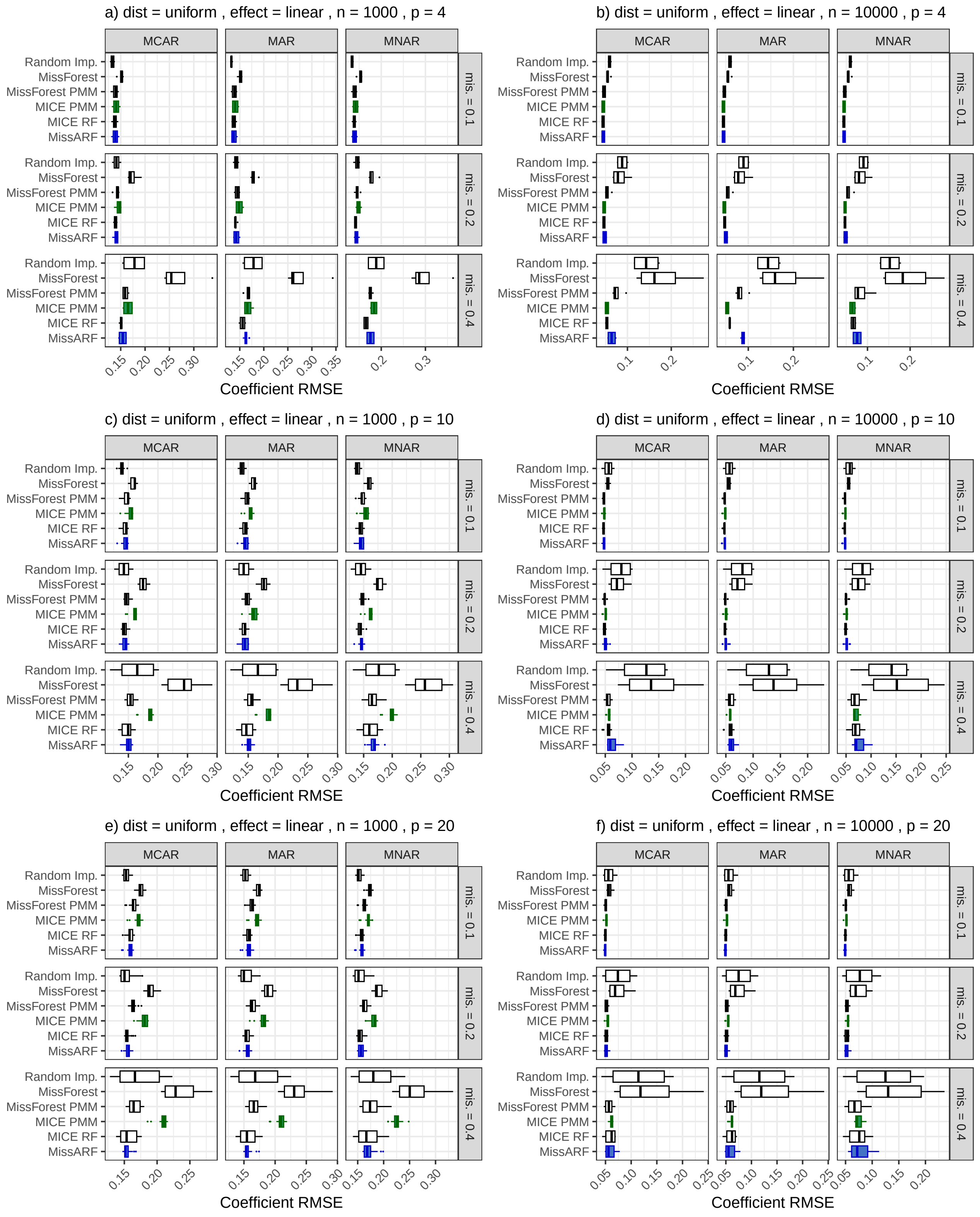

Figure S84: **RMSE of the regression coefficients** of the uniform distribution setting with a linear effect over different missingness patterns, dimensionality (p) and missingness rates (mis.) with $n = 1000$ (left) and $n = 10,000$ (right). The boxplots are plotted over the features, with MissARF (blue) and MICE PMM (green) highlighted.

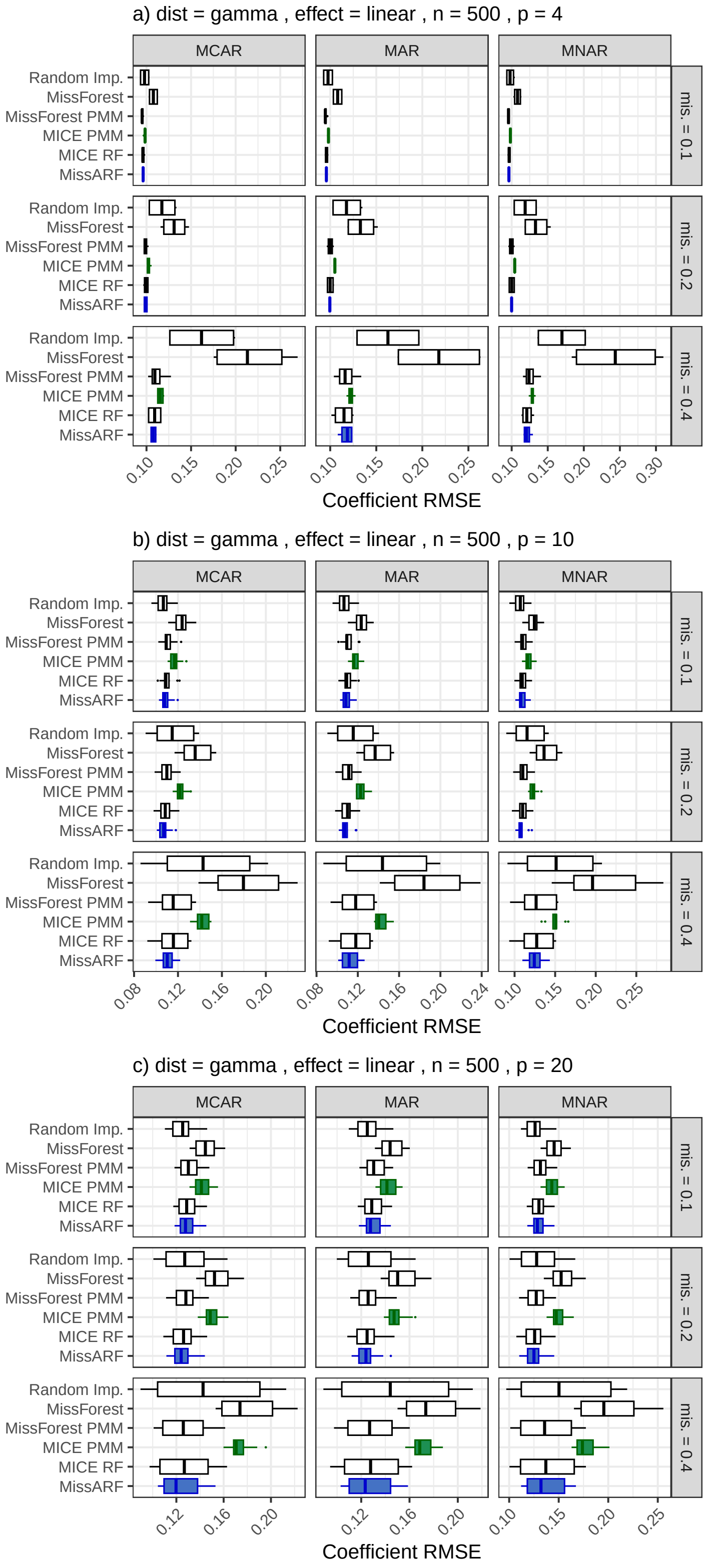

Figure S85: **RMSE of the regression coefficients** of the gamma distribution setting with a linear effect over different missingness patterns, dimensionality (p) and missingness rates (mis.) with $n = 500$. The boxplots are plotted over the features, with MissARF (blue) and MICE PMM (green) highlighted.

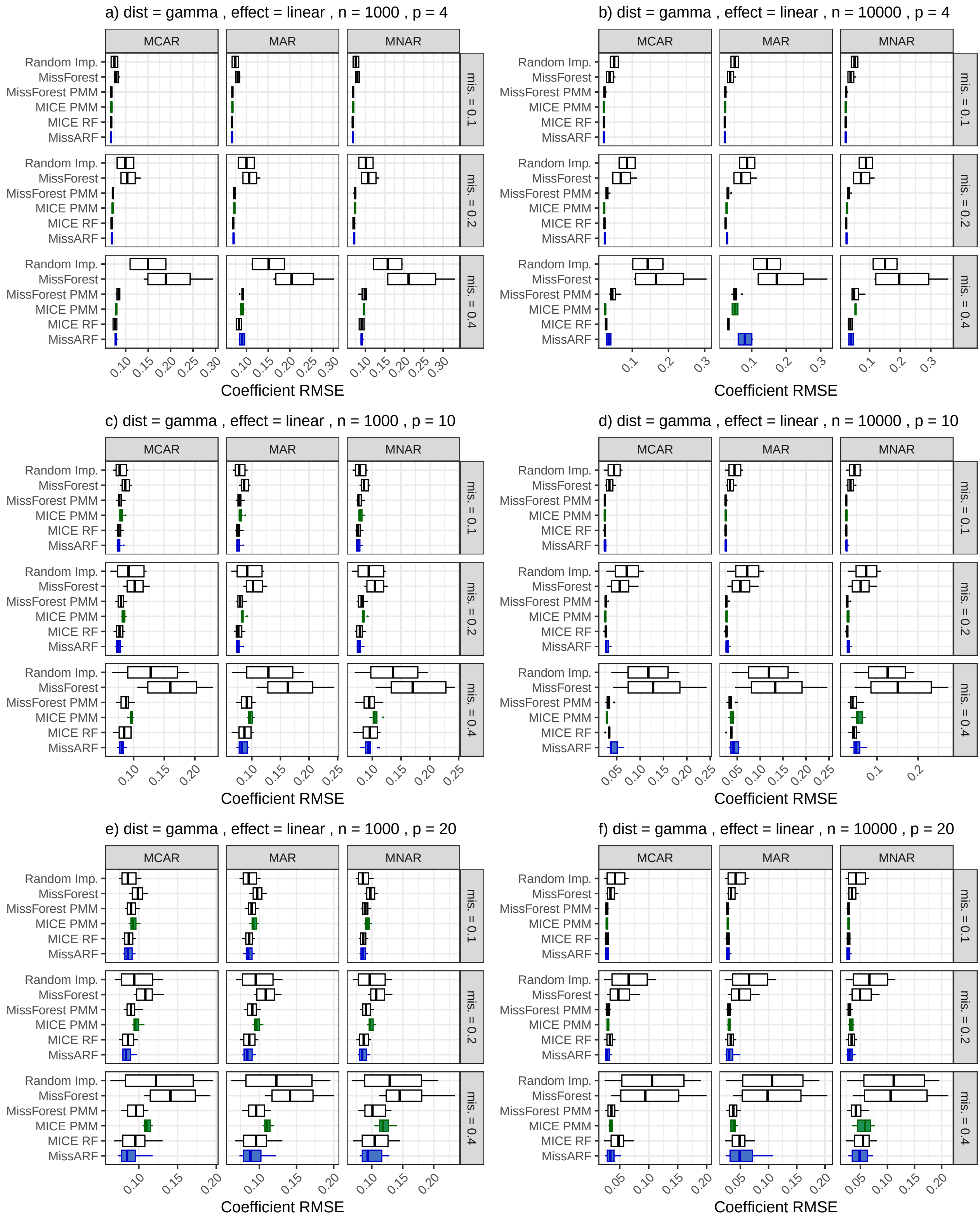

Figure S86: **RMSE of the regression coefficients** of the gamma distribution setting with a linear effect over different missingness patterns, dimensionality (p) and missingness rates (mis.) with $n = 1000$ (left) and $n = 10,000$ (right). The boxplots are plotted over the features, with MissARF (blue) and MICE PMM (green) highlighted.

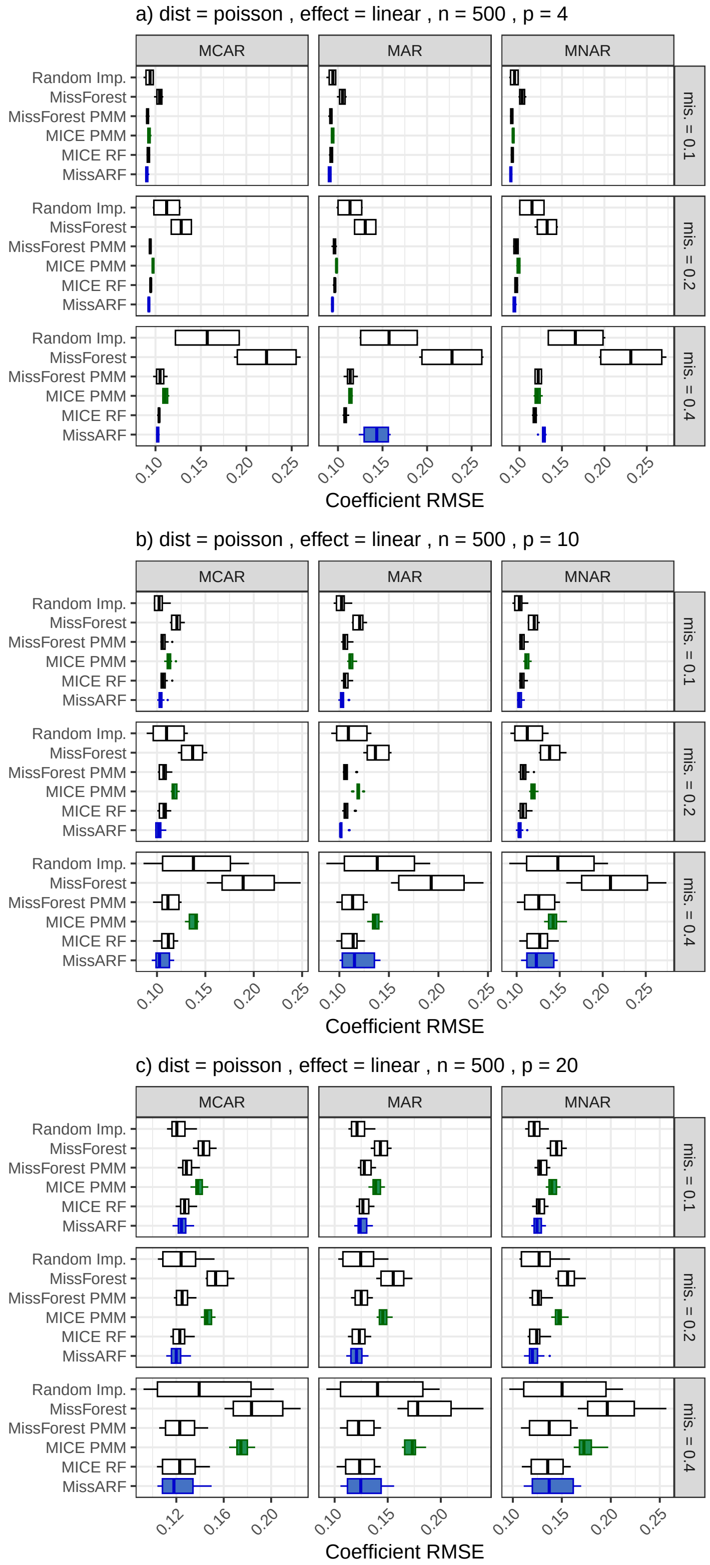

Figure S87: **RMSE of the regression coefficients** of the Poisson distribution setting with a linear effect over different missingness patterns, dimensionality (p) and missingness rates (mis.) with $n = 500$. The boxplots are plotted over the features, with MissARF (blue) and MICE PMM (green) highlighted.

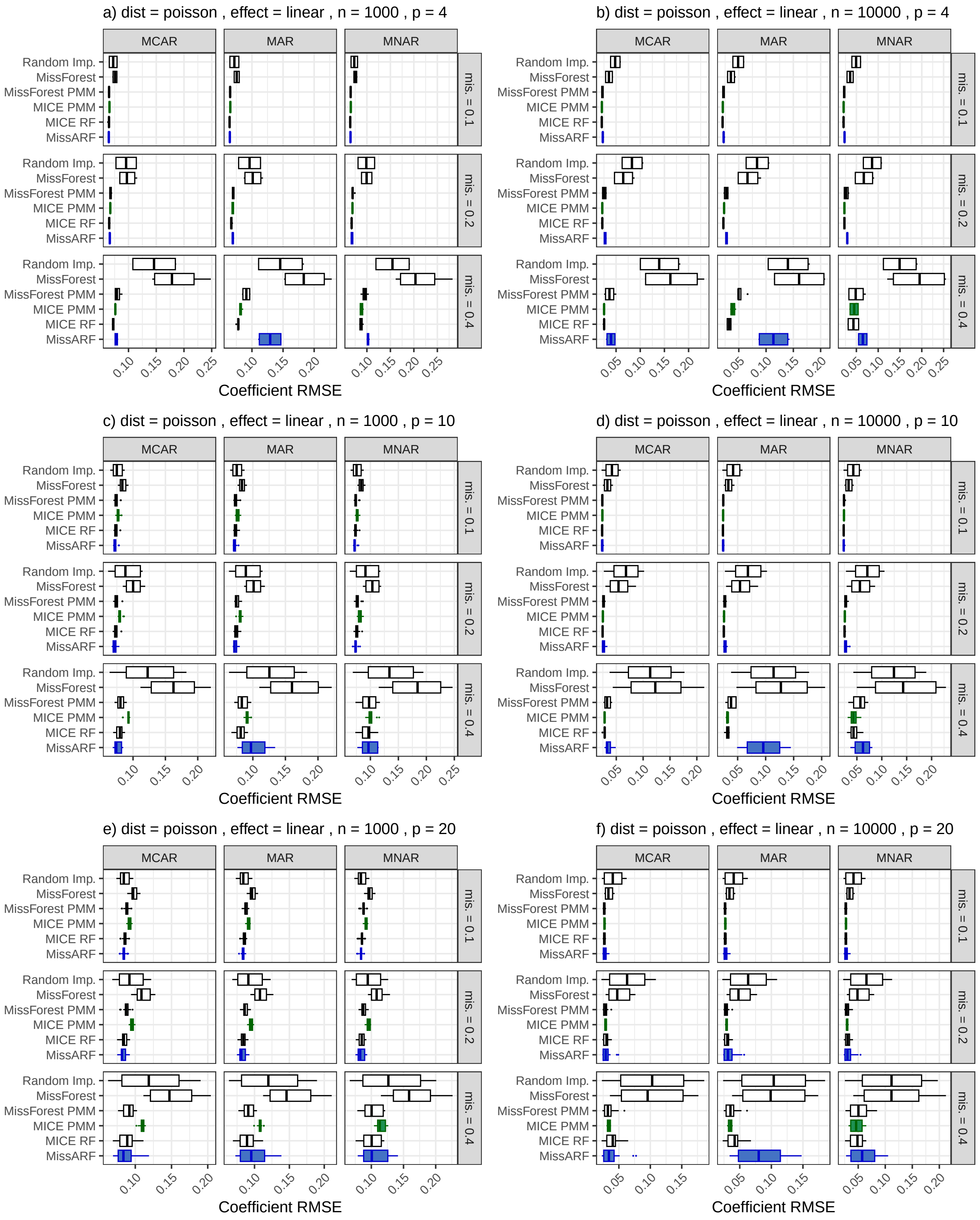

Figure S88: **RMSE of the regression coefficients** of the Poisson distribution setting with a linear effect over different missingness patterns, dimensionality (p) and missingness rates (mis.) with $n = 1000$ (left) and $n = 10,000$ (right). The boxplots are plotted over the features, with MissARF (blue) and MICE PMM (green) highlighted.

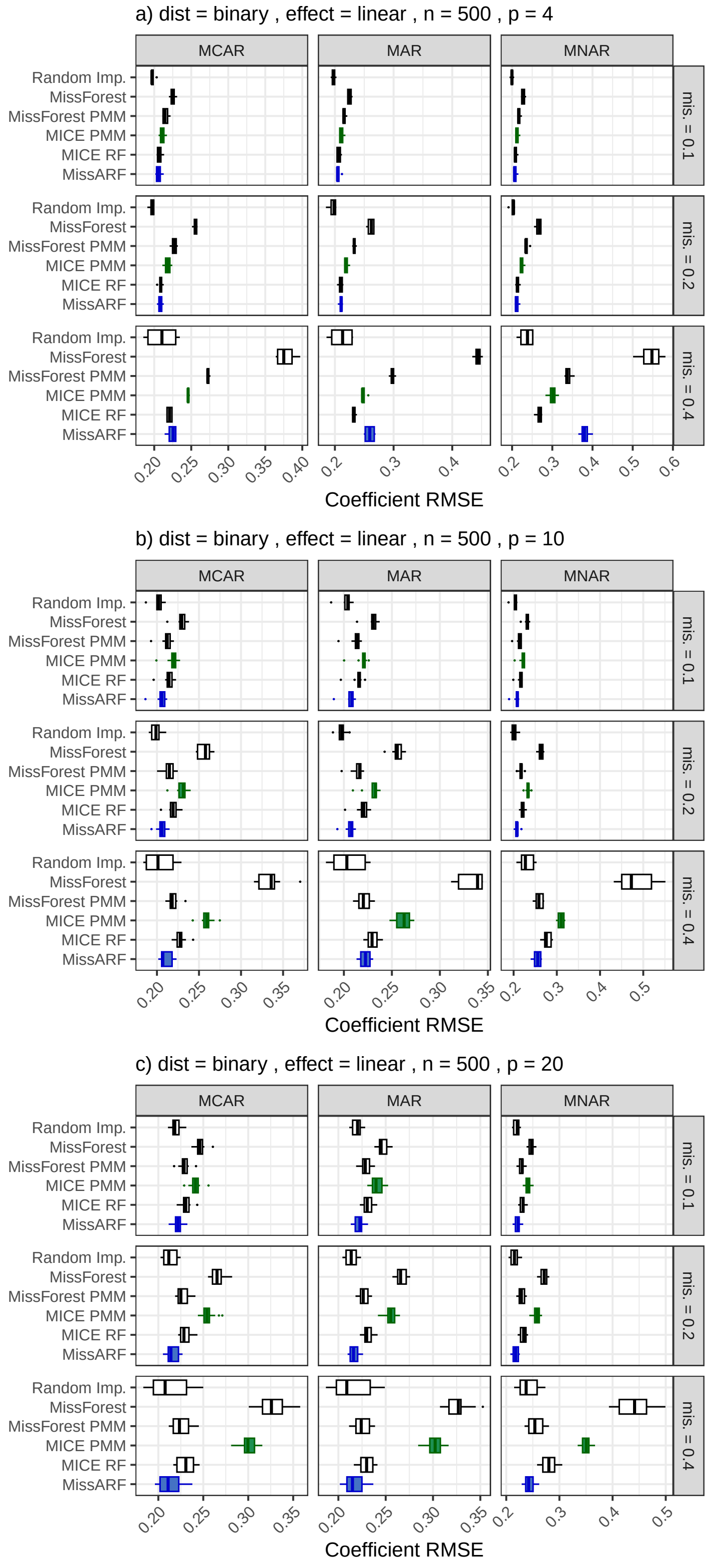

Figure S89: **RMSE of the regression coefficients** of the binary distribution setting with a linear effect over different missingness patterns, dimensionality (p) and missingness rates (mis.) with $n = 500$. The boxplots are plotted over the features, with MissARF (blue) and MICE PMM (green) highlighted.

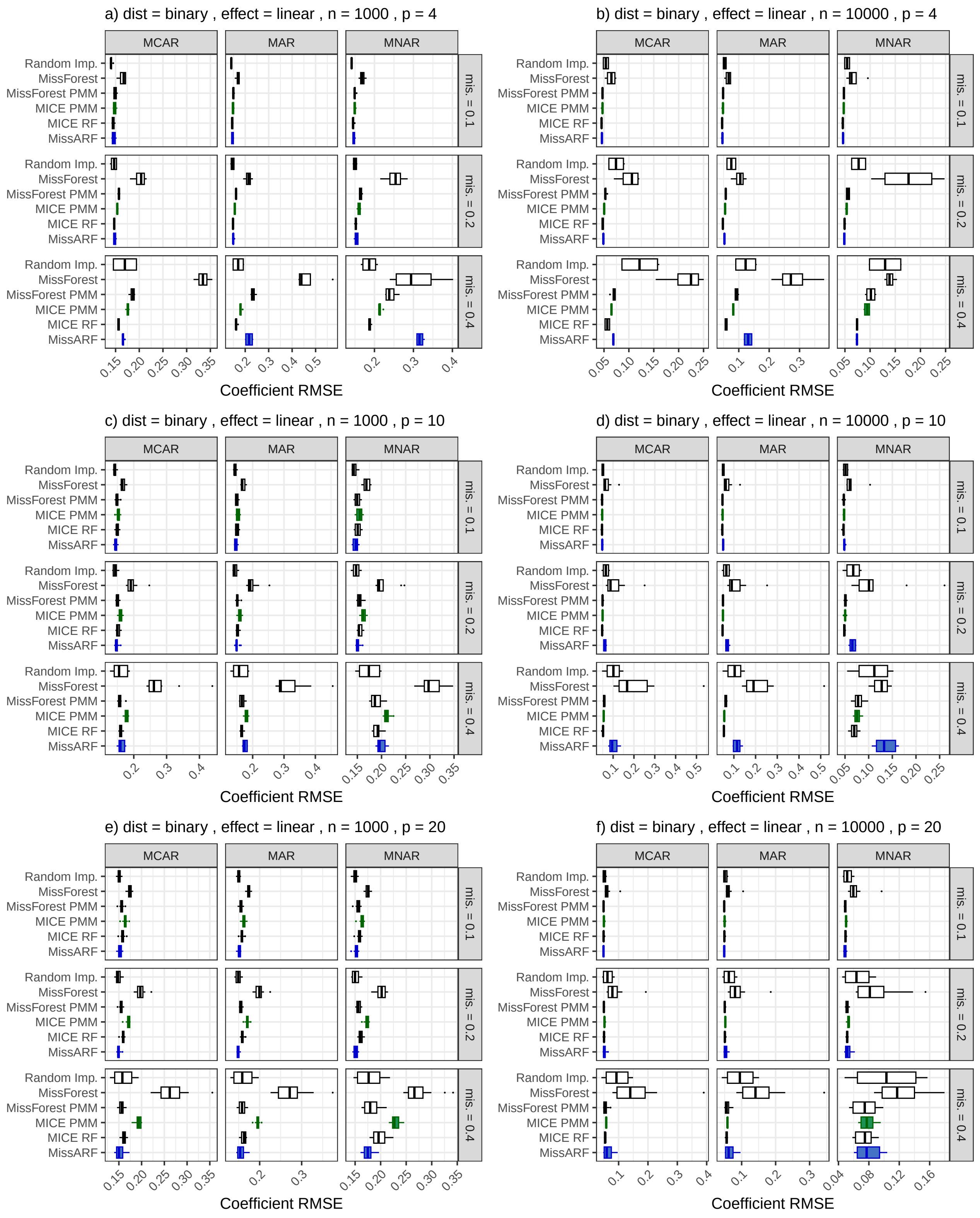

Figure S90: **RMSE of the regression coefficients** of the binary distribution setting with a linear effect over different missingness patterns, dimensionality (p) and missingness rates (mis.) with $n = 1000$ (left) and $n = 10,000$ (right). The boxplots are plotted over the features, with MissARF (blue) and MICE PMM (green) highlighted.

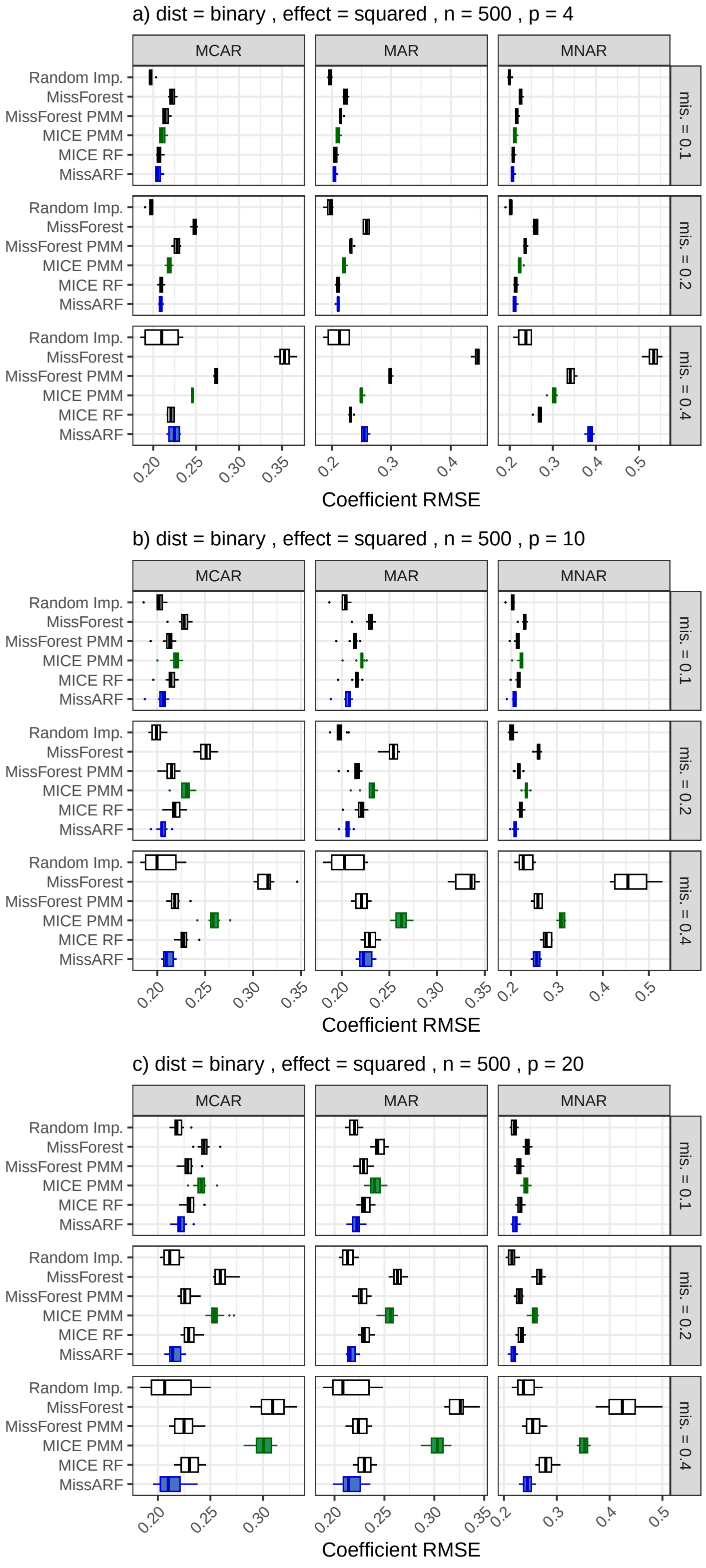

Figure S91: **RMSE of the regression coefficients** of the binary distribution setting with a squared effect over different missingness patterns, dimensionality (p) and missingness rates (mis.) with $n = 500$. The boxplots are plotted over the features, with MissARF (blue) and MICE PMM (green) highlighted.

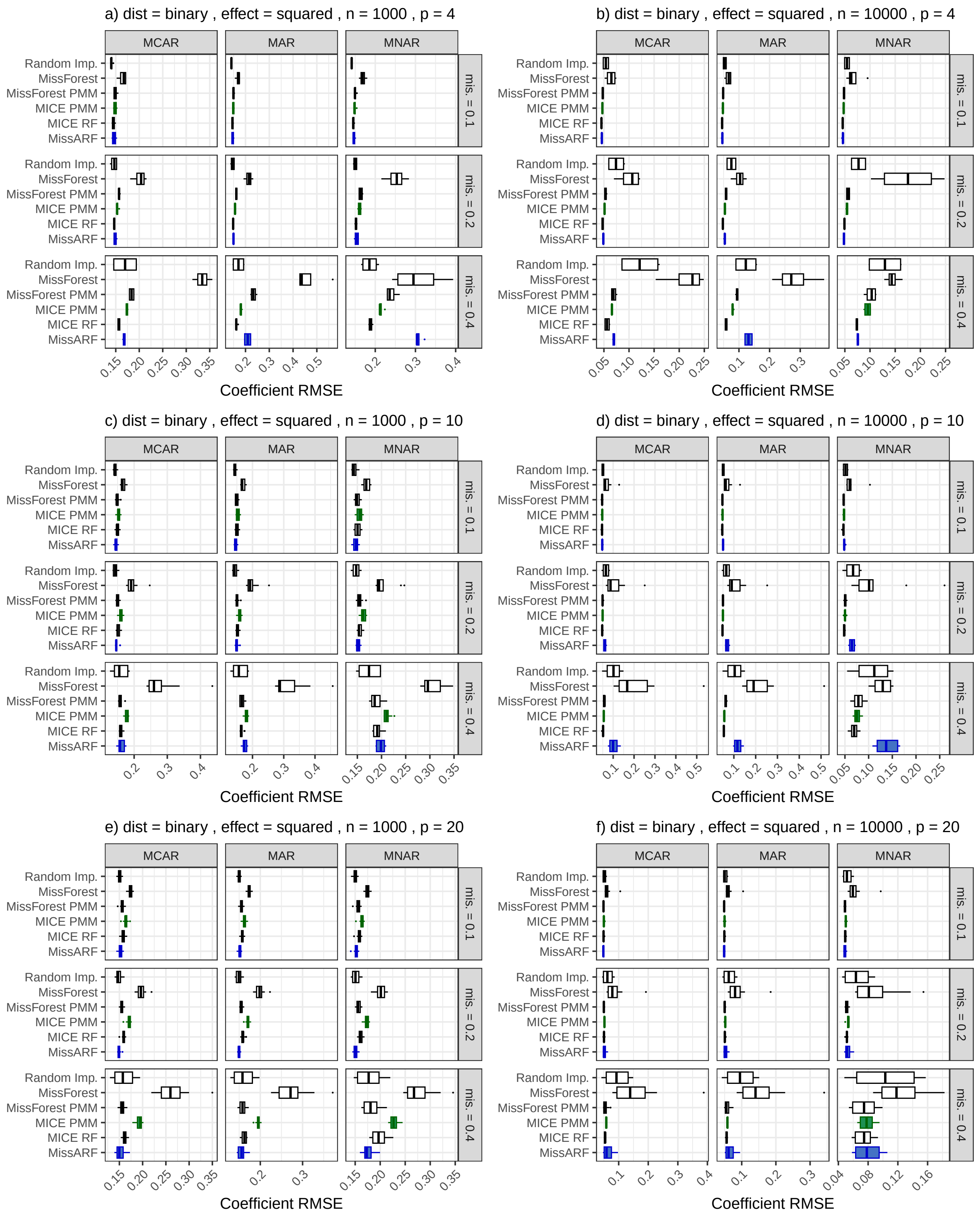

Figure S92: **RMSE of the regression coefficients** of the binary distribution setting with a squared effect over different missingness patterns, dimensionality (p) and missingness rates (mis.) with $n = 1000$ (left) and $n = 10,000$ (right). The boxplots are plotted over the features, with MissARF (blue) and MICE PMM (green) highlighted.

2.3.2 Category 2: PMM methods struggle, MissARF performs well

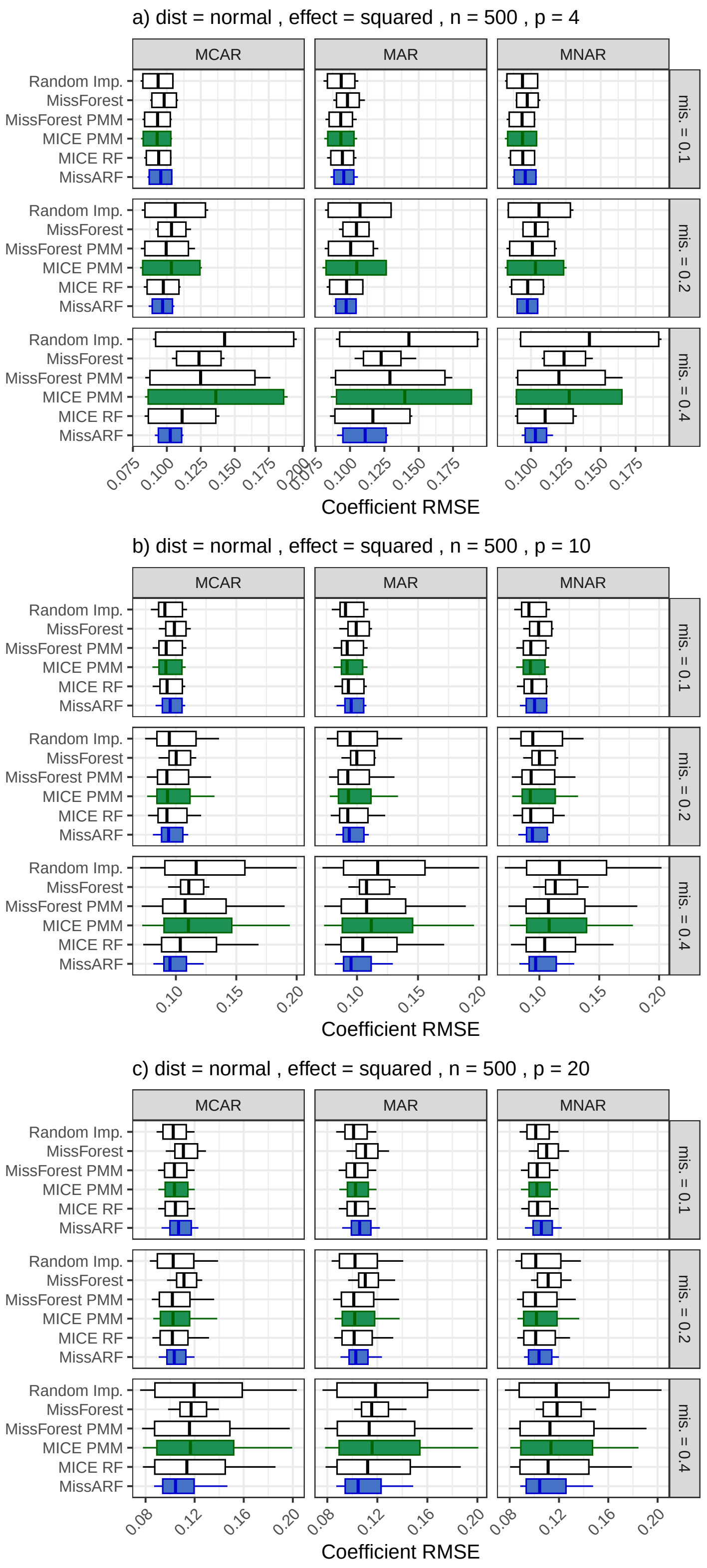

Figure S93: **RMSE of the regression coefficients** of the normal distribution setting with a squared effect over different missingness patterns, dimensionality (p) and missingness rates (mis.) with $n = 500$. The boxplots are plotted over the features, with MissARF (blue) and MICE PMM (green) highlighted.

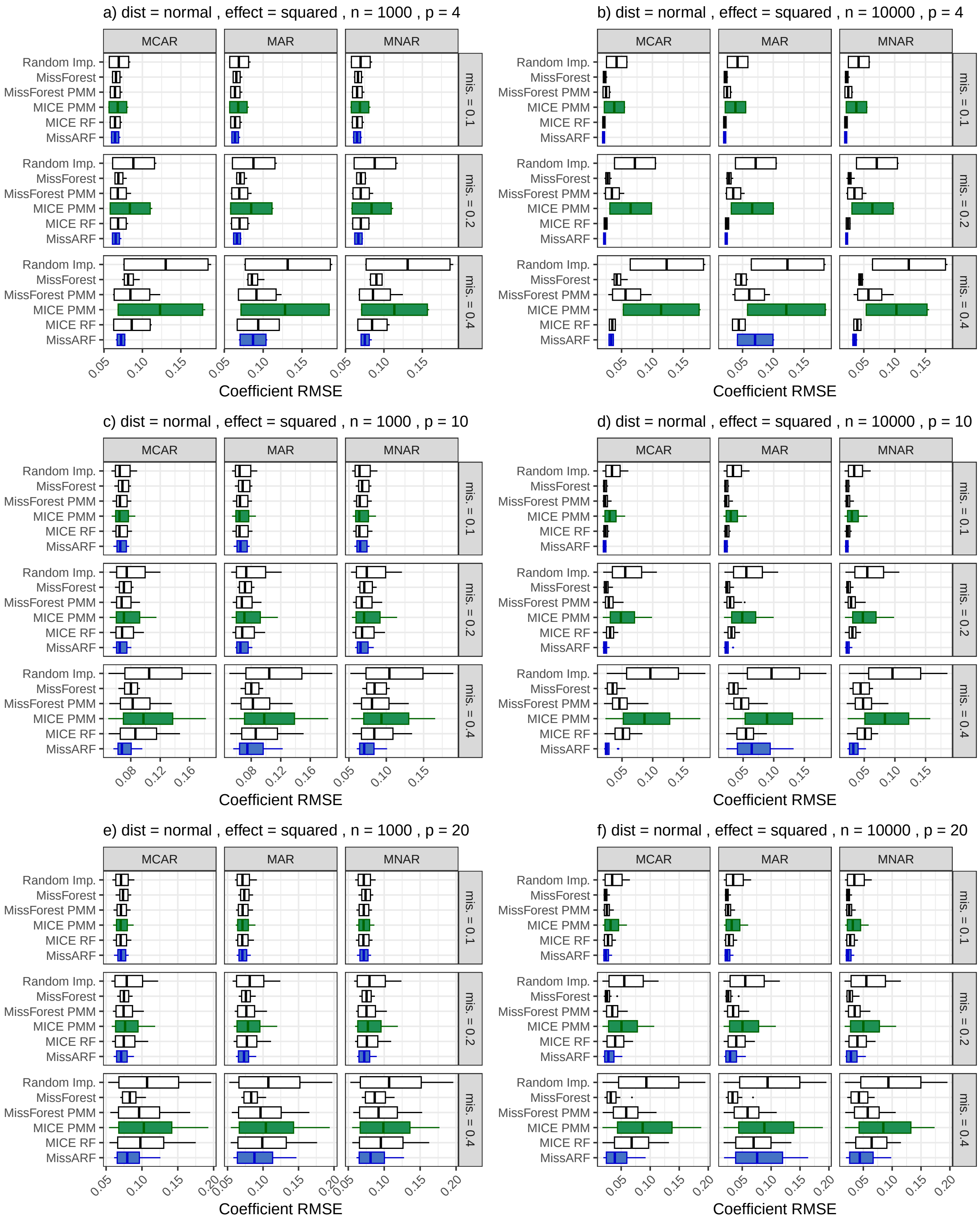

Figure S94: **RMSE of the regression coefficients** of the normal distribution setting with a squared effect over different missingness patterns, dimensionality (p) and missingness rates (mis.) with $n = 1000$ (left) and $n = 10,000$ (right). The boxplots are plotted over the features, with MissARF (blue) and MICE PMM (green) highlighted.

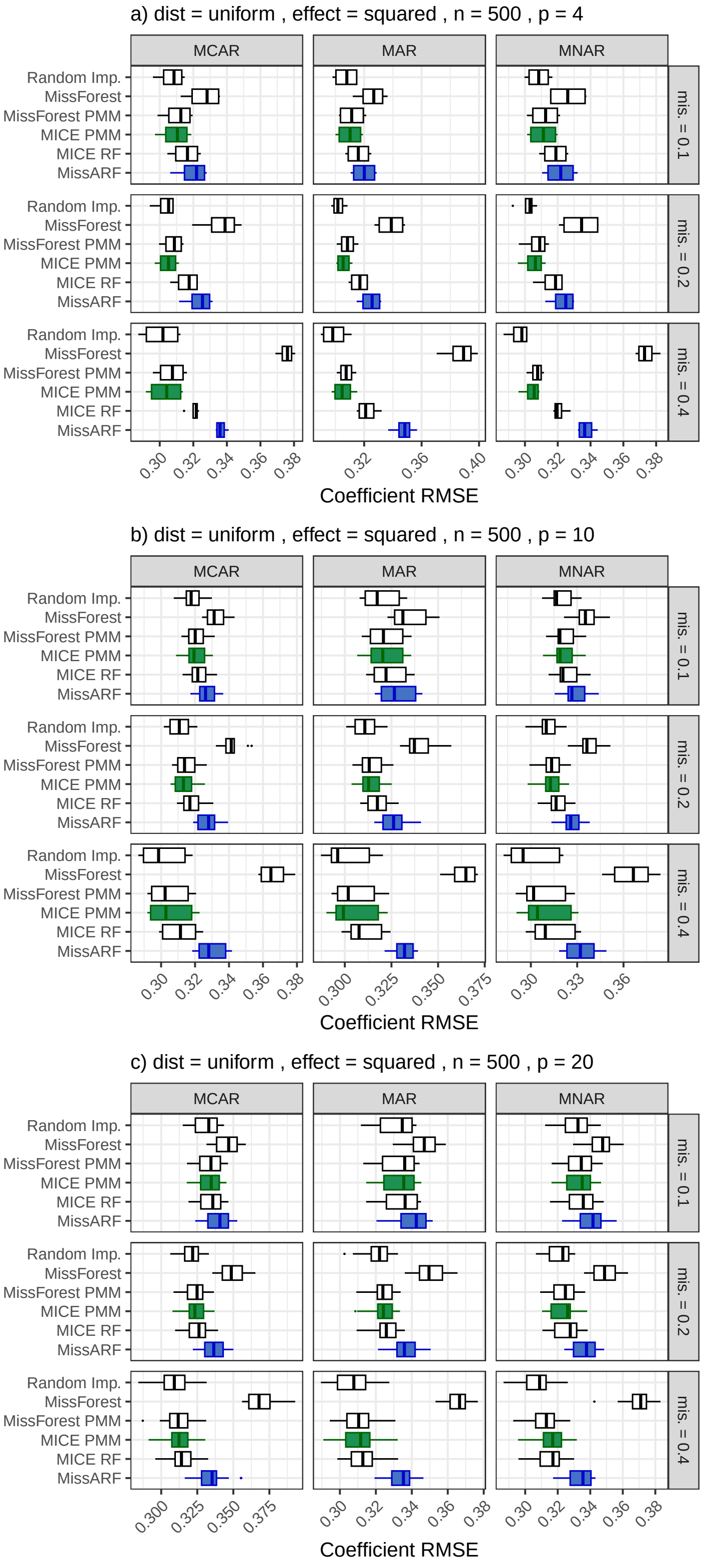

Figure S95: **RMSE of the regression coefficients** of the uniform distribution setting with a squared effect over different missingness patterns, dimensionality (p) and missingness rates (mis.) with $n = 500$. The boxplots are plotted over the features, with MissARF (blue) and MICE PMM (green) highlighted.

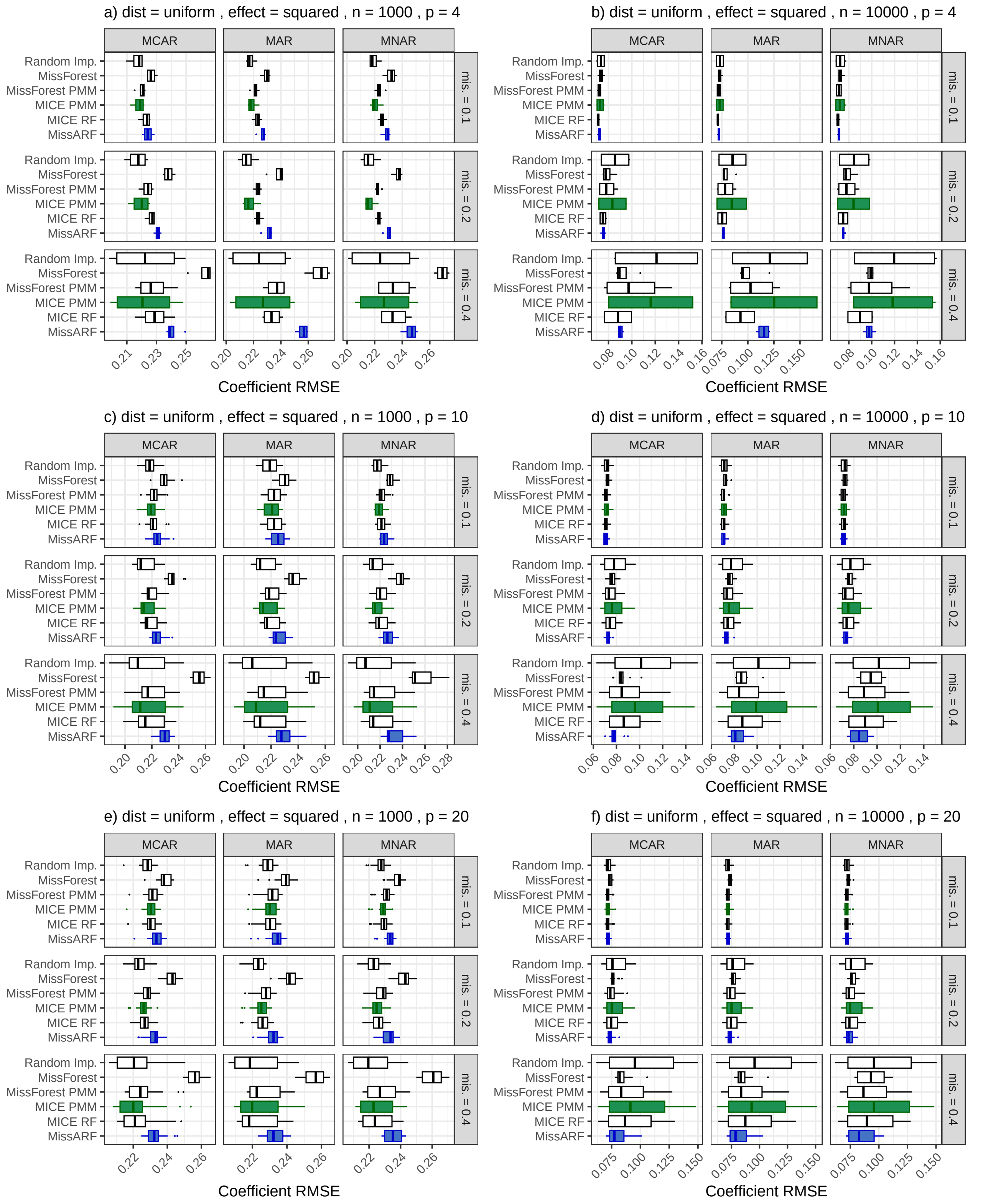

Figure S96: **RMSE of the regression coefficients** of the uniform distribution setting with a squared effect over different missingness patterns, dimensionality (p) and missingness rates (mis.) with $n = 1000$ (left) and $n = 10,000$ (right). The boxplots are plotted over the features, with MissARF (blue) and MICE PMM (green) highlighted.

2.3.3 Category 3: All methods perform poorly

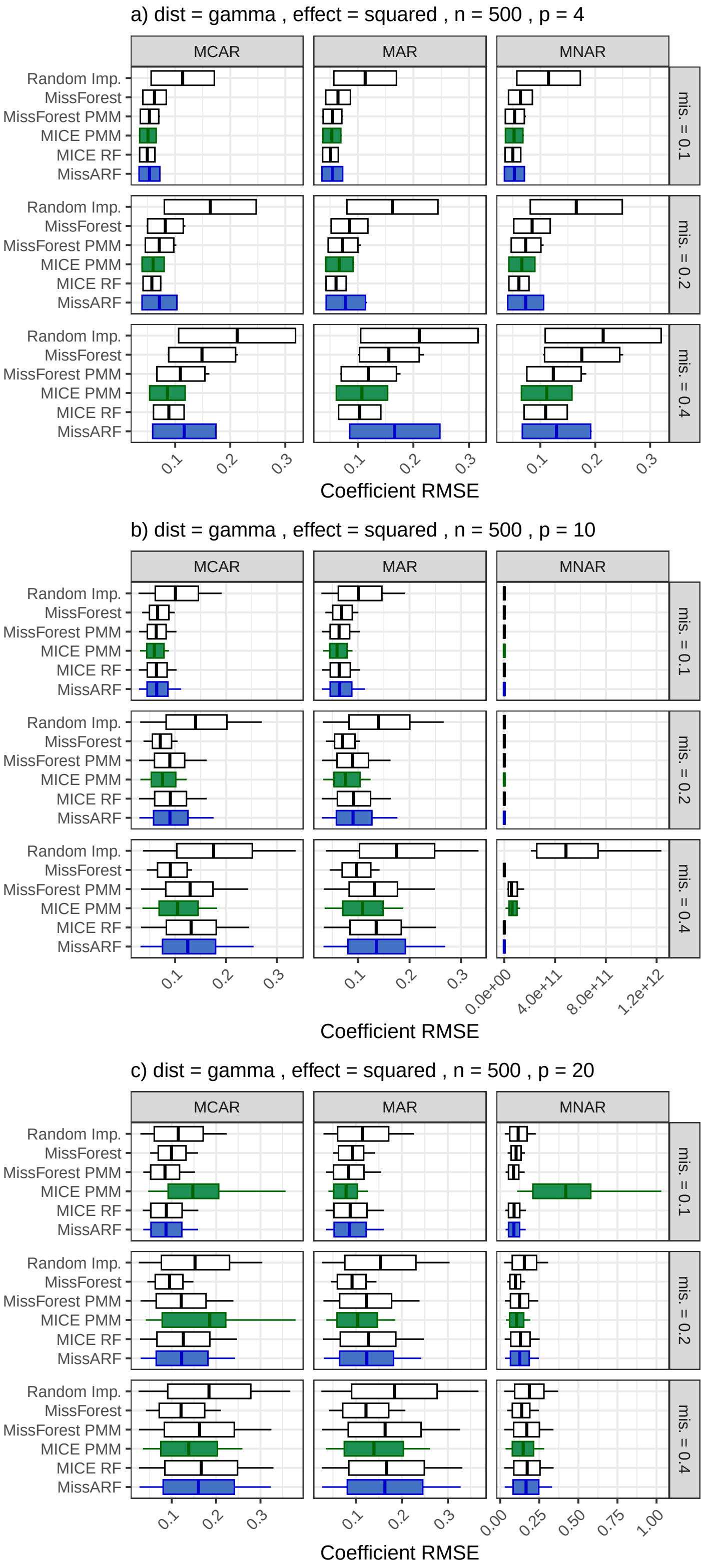

Figure S97: **RMSE of the regression coefficients** of the gamma distribution setting with a squared effect over different missingness patterns, dimensionality (p) and missingness rates (mis.) with $n = 500$. The boxplots are plotted over the features, with MissARF (blue) and MICE PMM (green) highlighted.

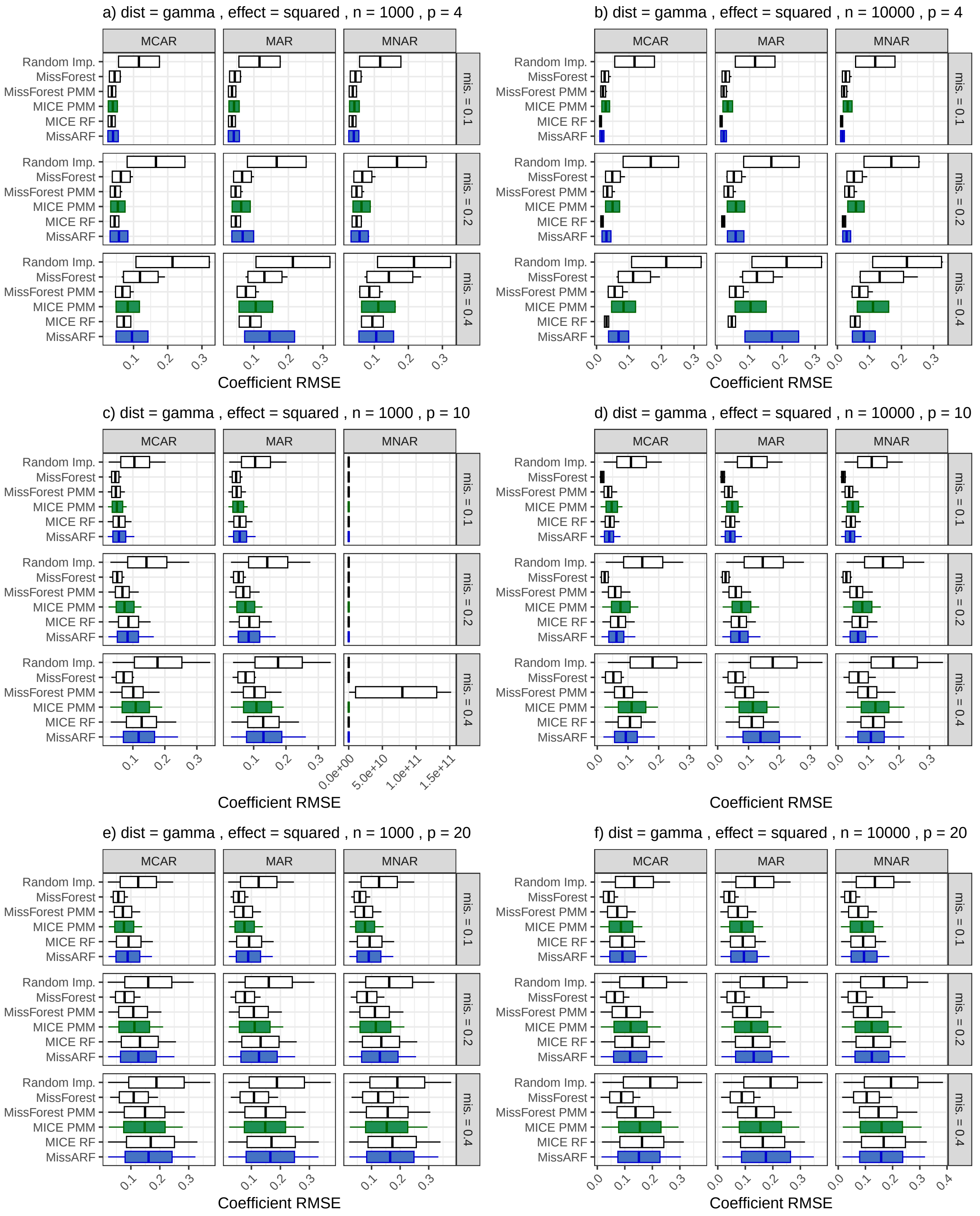

Figure S98: **RMSE of the regression coefficients** of the gamma distribution setting with a squared effect over different missingness patterns, dimensionality (p) and missingness rates (mis.) with $n = 1000$ (left) and $n = 10,000$ (right). The boxplots are plotted over the features, with MissARF (blue) and MICE PMM (green) highlighted.

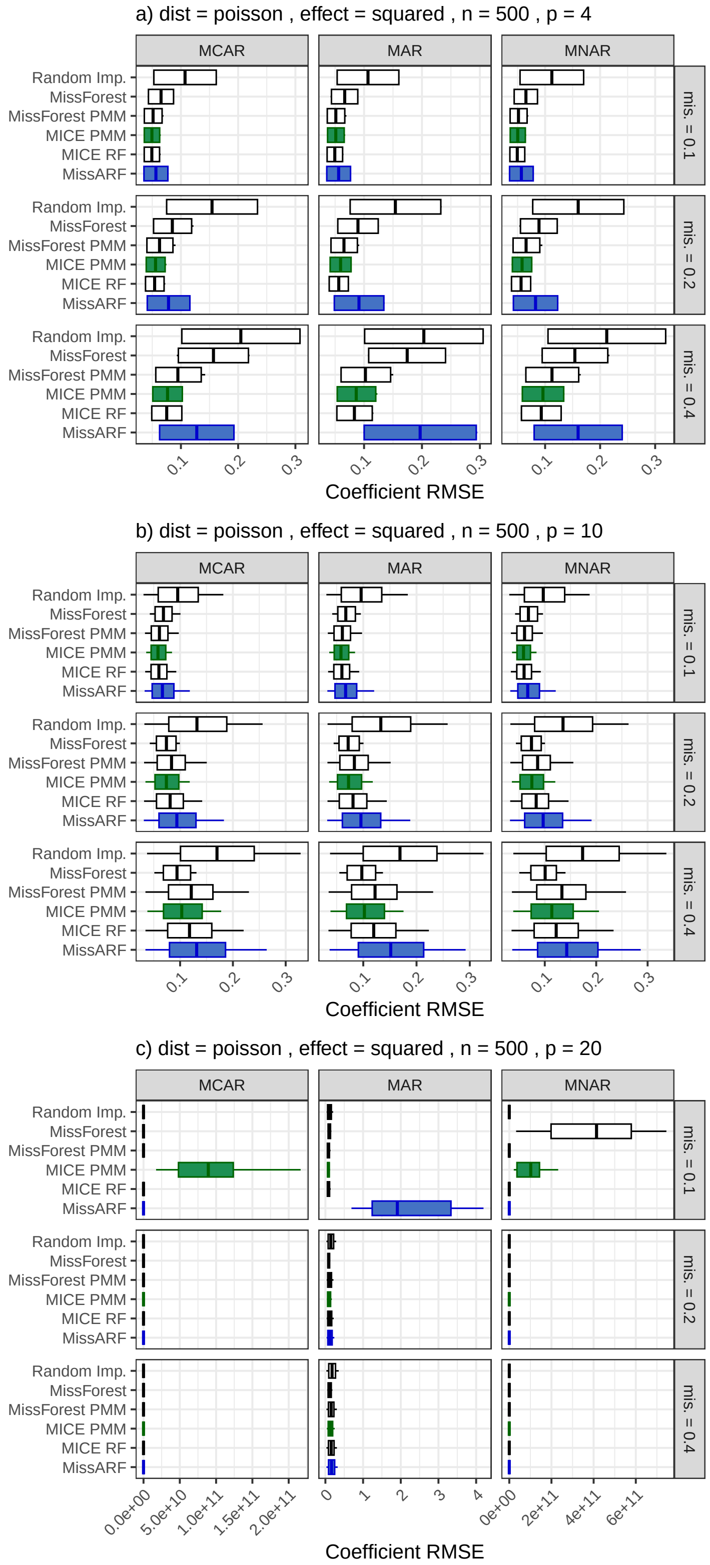

Figure S99: **RMSE of the regression coefficients** of the Poisson distribution setting with a squared effect over different missingness patterns, dimensionality (p) and missingness rates (mis.) with $n = 500$. The boxplots are plotted over the features, with MissARF (blue) and MICE PMM (green) highlighted.

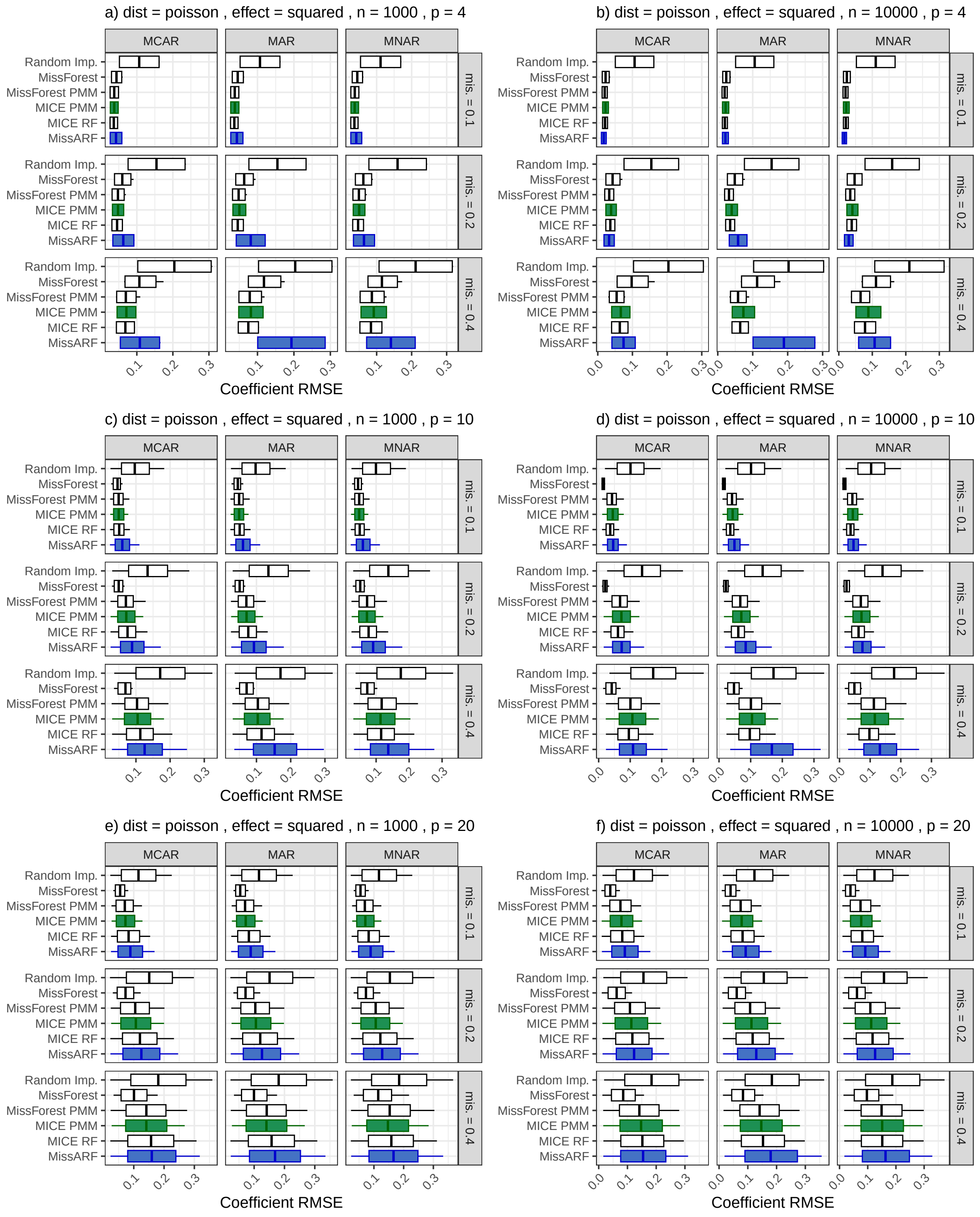

Figure S100: **RMSE of the regression coefficients** of the Poisson distribution setting with a squared effect over different missingness patterns, dimensionality (p) and missingness rates (mis.) with $n = 1000$ (left) and $n = 10,000$ (right). The boxplots are plotted over the features, with MissARF (blue) and MICE PMM (green) highlighted.

3 Real Data

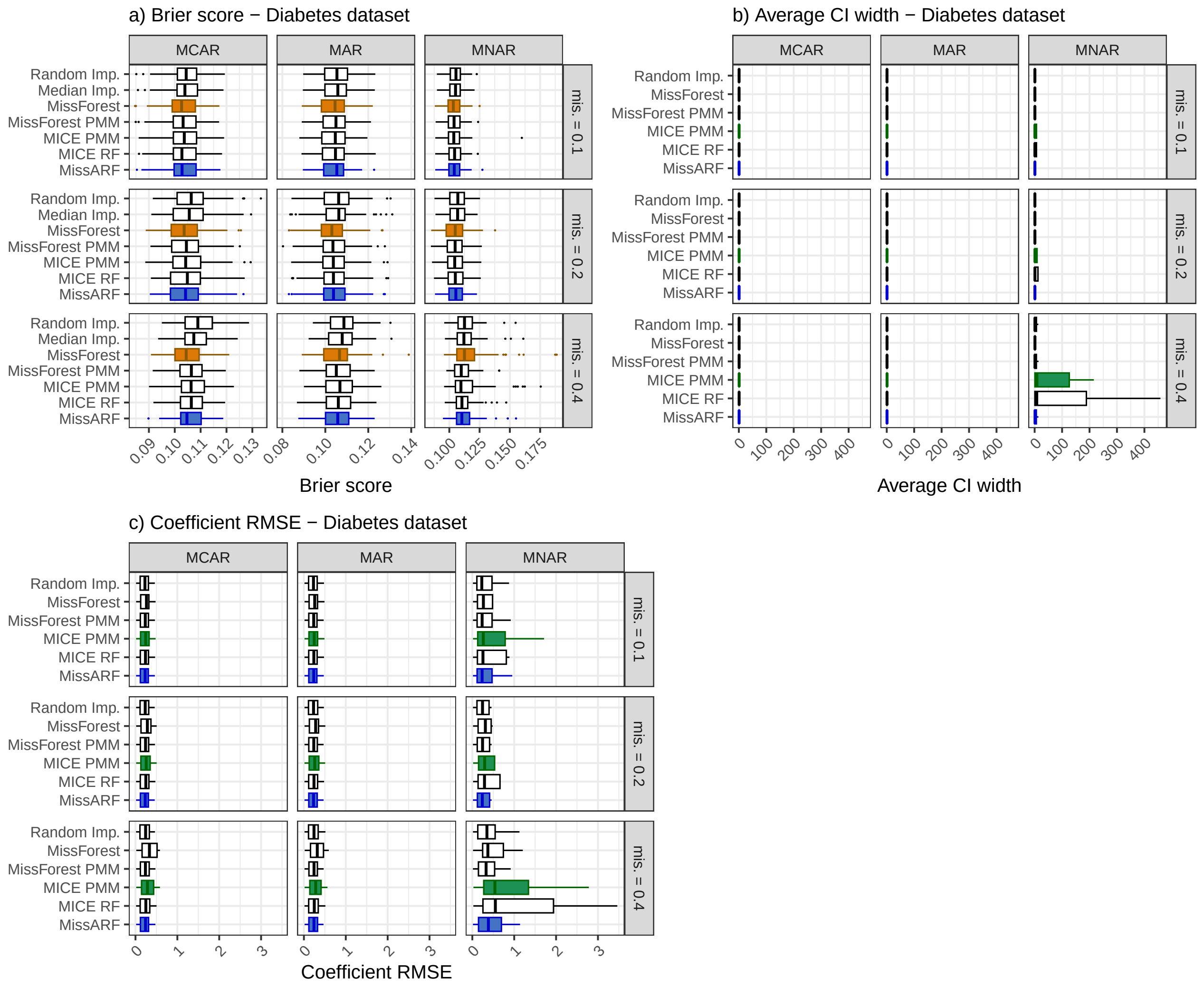

Figure S101: **Real data example results** over the different missingness proportions and patterns for a) Brier score, b) average width of the confidence intervals and c) the RMSE of the regression coefficients. The boxplots are plotted over the replicates in a) and over the features in b)-c) with MissARF (blue), MissForest (orange) and MICE PMM (green) highlighted.

4 Runtime comparisons

Tables S1 and S2 show more detailed runtime comparisons over different n. In Figures S102 and S103, we investigate runtime comparisons in more detail over different dimensions and missingness proportions. The runtime comparisons were performed using the following package versions: `missRanger_2.6.1`, `arf_0.2.4`, `mice_3.17.0`, `simstudy_0.8.0`, `missMethods_0.4.0`.

Method	$n = 500$		$n = 1000$		$n = 10000$	
	Single	Multiple	Single	Multiple	Single	Multiple
Random Imp.	<0.01	0.02	<0.01	0.02	<0.01	0.07
Median Imp.	<0.01	-	<0.01	-	0.01	-
MissForest	0.92	19.59	2.17	41.59	45.11	906.93
MissForest PMM	0.98	19.53	2.20	42.13	46.41	913.82
MICE PMM	0.09	1.50	0.11	1.86	0.48	8.46
MICE RF	0.44	8.26	0.84	15.66	10.63	211.44
MissARF	0.93	0.87	1.84	1.70	40.42	40.77

Table S1: Runtime comparison of the different methods, executed with one thread. The measured average times in seconds for single and multiple imputation for each of the different n are compared.

Method	$n = 500$		$n = 1000$		$n = 10000$	
	Single	Multiple	Single	Multiple	Single	Multiple
Random Imp.	<0.01	0.01	<0.01	0.02	0.01	0.06
Median Imp.	<0.01	-	<0.01	-	0.01	-
MissForest	0.27	5.59	0.40	7.70	4.86	98.33
MissForest PMM	0.28	5.58	0.41	7.81	5.01	99.43
MICE PMM	0.09	1.57	0.12	1.95	0.50	8.98
MICE RF	0.43	8.24	0.66	12.73	4.96	98.42
MissARF	1.20	1.23	1.38	1.44	6.34	6.26

Table S2: Runtime comparison of the different methods, executed with 16 threads. The measured average times in seconds for single and multiple imputation for each of the different n are compared.

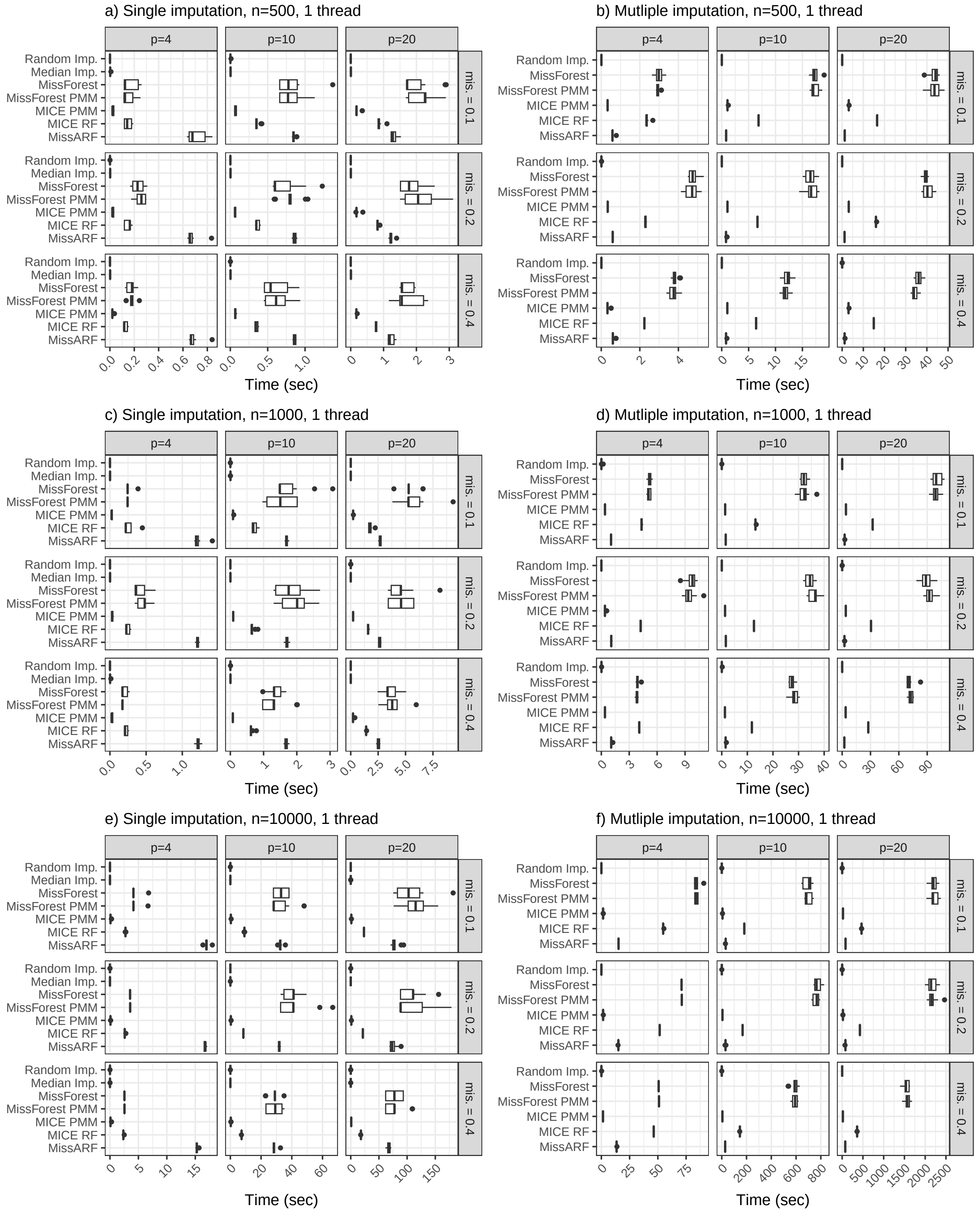

Figure S102: **Runtime comparison with one thread** of the different imputation methods across the dimensionality (p) and missingness proportions (mis.) with different n. The boxplots for single imputation (left) and multiple imputation (right) are plotted over the 10 replicates. The runtime is shown in seconds (sec).

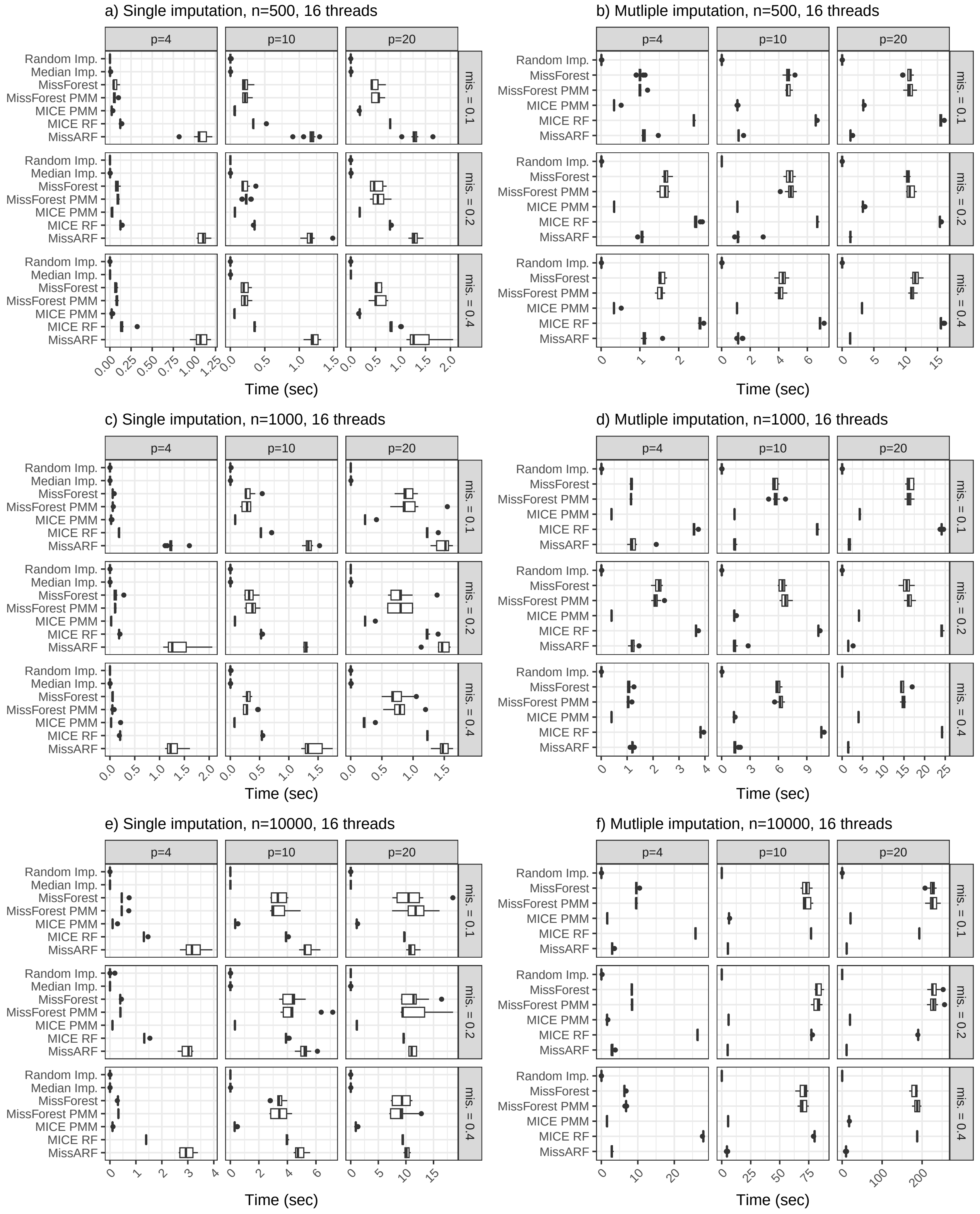

Figure S103: **Runtime comparison with 16 threads** of the different imputation methods across the dimensionality (p) and missingness proportions (mis.) with different n. The boxplots for single imputation (left) and multiple imputation (right) are plotted over the 10 replicates. The runtime is shown in seconds (sec).

5 Further experiments

5.1 Possible hyperparameter tuning

In the discussion, we state that MissARF has difficulties with higher missingness proportions and that the multiple imputation would benefit from a larger diversity of imputed values. We examine if the following approaches would improve the performance: 1) Adjusting the minimum node size, 2) increasing the number of trees, and 3) increasing the number of multiple imputations. For that we selected an example in which the coverage of MissARF was below the nominal level (~ 0.9) for higher missingness: normal distribution with a linear effect with $p = 4$, $n = 1000$, and 0.4 missingness with a MAR pattern (See Figure S42a), S62a) , S82a)).

Different number of node sizes: The idea is that the larger the leaf node size, the more different variable values are considered for each imputed value, which could be helpful if the missingness rate is high. In Figure S104, different minimum node sizes of 2-400 of a logarithmic scale grid are visualized. Very small node sizes lead to poor coverage and it increases drastically to a good coverage already by a node size of 10. We get a coverage above 0.9 for node sizes between 10 and 80 with a peak around 31 at 0.92. Then the coverage gets poorer and decreases with increasing node size. So choosing a node size of 31 instead of 10 improved the coverage by 0.02. The average confidence interval width starts with a small width for small node sizes, gets above 0.35 between 10 and 50 nodes with a peak around 20 and then decreases with larger node sizes. The RMSE starts with higher values for small node sizes and decreases with the number of node sizes. It is under 0.11 for node sizes between 10 and 110 with a minimum around 40 and increases after that with larger node sizes.

Number of trees: We examine the effect of different numbers of trees ranging from 10 to 160 for different minimum node sizes 10, 20 and 30 on coverage, average CI width and coefficient RMSE (Figure S105) and on the NRMSE (Figure S106). We chose $p = 20$ for the NRMSE because MissARF performed worse than MissForest in the experiments and we aim to improve it.

We observe that for a small number of node size 10, more trees harm the performance, whereas it does not have an effect with 20 or 30. For a minimum node size 10, the NRMSE has an optimum around 40, and then the performance weakens with growing number of trees. For the coverage we observe a similar effect with an optimum at 70. In both cases we observe worse performance for 10 as the minimum node size. For the NRMSE, we observe better performance for 20 as a minimum node size. For the coverage, and coefficient RMSE, the number of node size of 30 has a slight advantage to 20. The average width is slightly larger than for 10.

More multiple imputations ($m = 40$): In our experiments, we chose a fixed number of $m = 20$ for simplicity and due to computational restrictions. However, White, Royston, and Wood[2] describe a rule of thumb for m being "at least equal to the percentage of incomplete cases"[2][1]. When repeating the experiment with $m = 40$ imputations as recommended by White, Royston, and Wood[2], we would expect more stable results. However, in this example (see Figure S107) we do not notice a big difference for coverage, average CI width and RMSE, and the boxplots are slightly wider than before.

Conclusion: All in all, we conclude that optimizing node size could improve the performance and therefore be considered as a hyperparameter to tune.

5.2 Smaller sample sizes

In practice, for example, in clinical research, we encounter small sample sizes. In the following, we examine the trend of the performance with smaller sample sizes for the normal distribution with a linear effect with $p = 4$ and 0.2 missingness with a MAR pattern for sample sizes between 50 and 500.

In general, the performance of all metrics decreases for sample sizes smaller than 150. For the NRMSE, it only decreases slightly for all methods except for random and median imputation, which do not learn from data and are thus not affected much by sample size (Figure S108). The Brier score (Figure S109) declines more steeply for all methods compared to the NRMSE. For setting II (multiple imputation) (Figure S110), all methods achieve higher coverage rates with wider average widths of the confidence intervals and higher RMSE values for a smaller sample size. All methods over-cover, except MissForest, which under-covers, and MICE PMM, which has constant good coverage close to 0.95. For $n = 50$, all methods, except MissForest, over-cover and have high values for the RMSE of the regression coefficients. While for MissForest and random imputation the coverage decreases with growing sample size, for the other methods it remains similar after a sample size of 100.

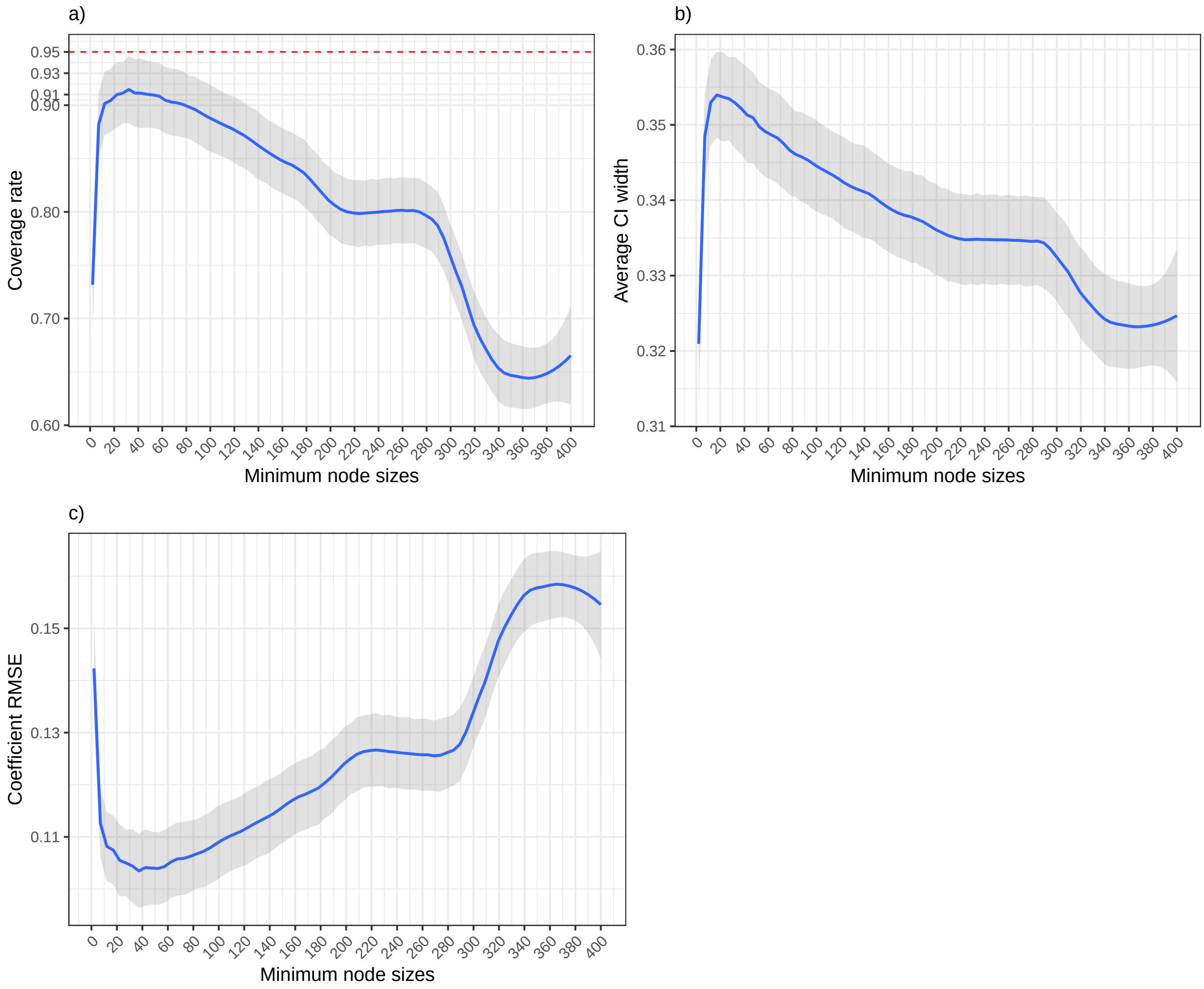

Figure S104: Mean values with the standard error for a) coverage rate, b) average confidence interval width and c) RMSE for minimum node sizes of 2-400 (100 points from a logarithmic scale) and 100 number of trees with MissARF for a normal distribution with a linear effect with $p = 4$, $n = 1000$, and 0.4 missingness with a MAR pattern over 1000 replications.

References

[1] Geert Molenberghs, Garrett Fitzmaurice, Michael G Kenward, Anastasios Tsiatis, and Geert Verbeke. *Handbook of Missing Data Methodology*. CRC Press, 2015.

[2] Ian R White, Patrick Royston, and Angela M Wood. Multiple imputation using chained equations: issues and guidance for practice. *Statistics in Medicine*, 30(4):377–399, 2011.

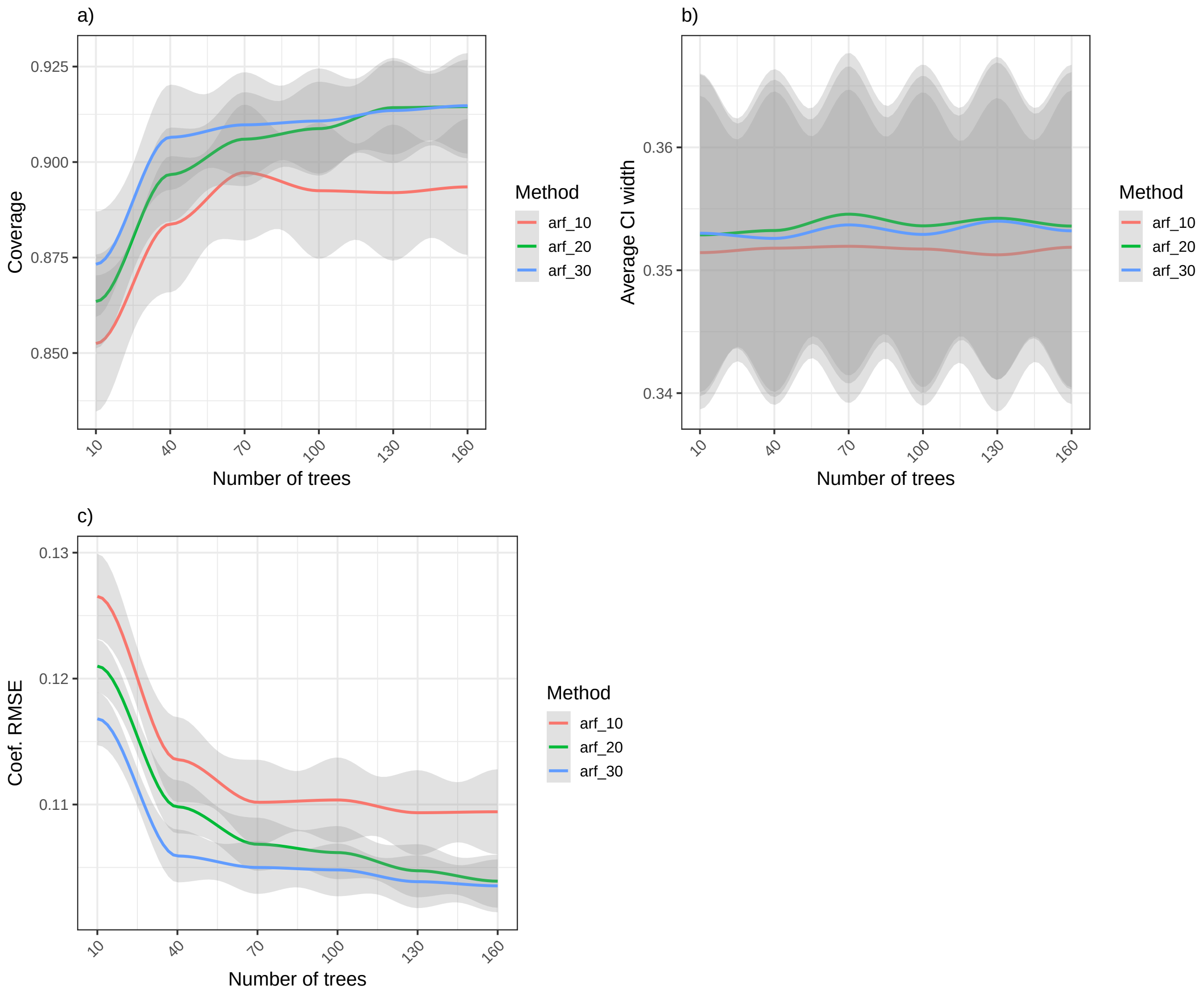

Figure S105: Mean values with the standard error for a) coverage rate, b) average confidence interval width and c) RMSE for minimum node sizes 10 (arf_10), 20 (arf_20) and 30 (arf_30) and 10-160 number of trees with MissARF for a normal distribution with a linear effect with $p = 4$, $n = 1000$, and 0.4 missingness with a MAR pattern over 1000 replications.

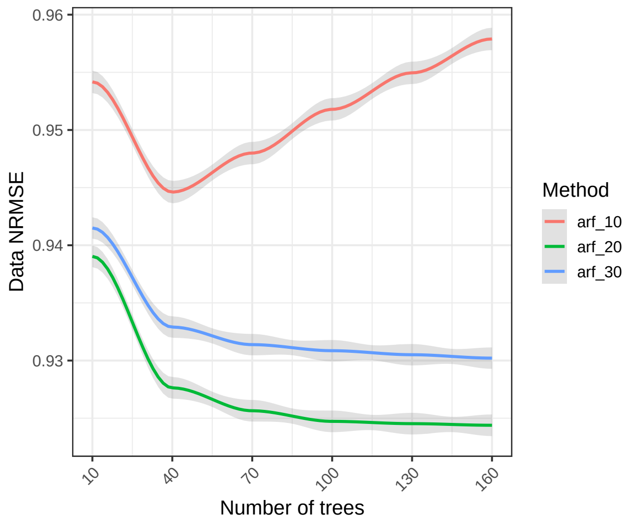

Figure S106: Mean values with the standard error for the NRMSE for minimum node sizes 10 (arf_10), 20 (arf_20) and 30 (arf_30) and 10-160 number of trees with MissARF for a normal distribution with a linear effect with $p = 20$, $n = 1000$, and 0.4 missingness with a MAR pattern over 1000 replications.

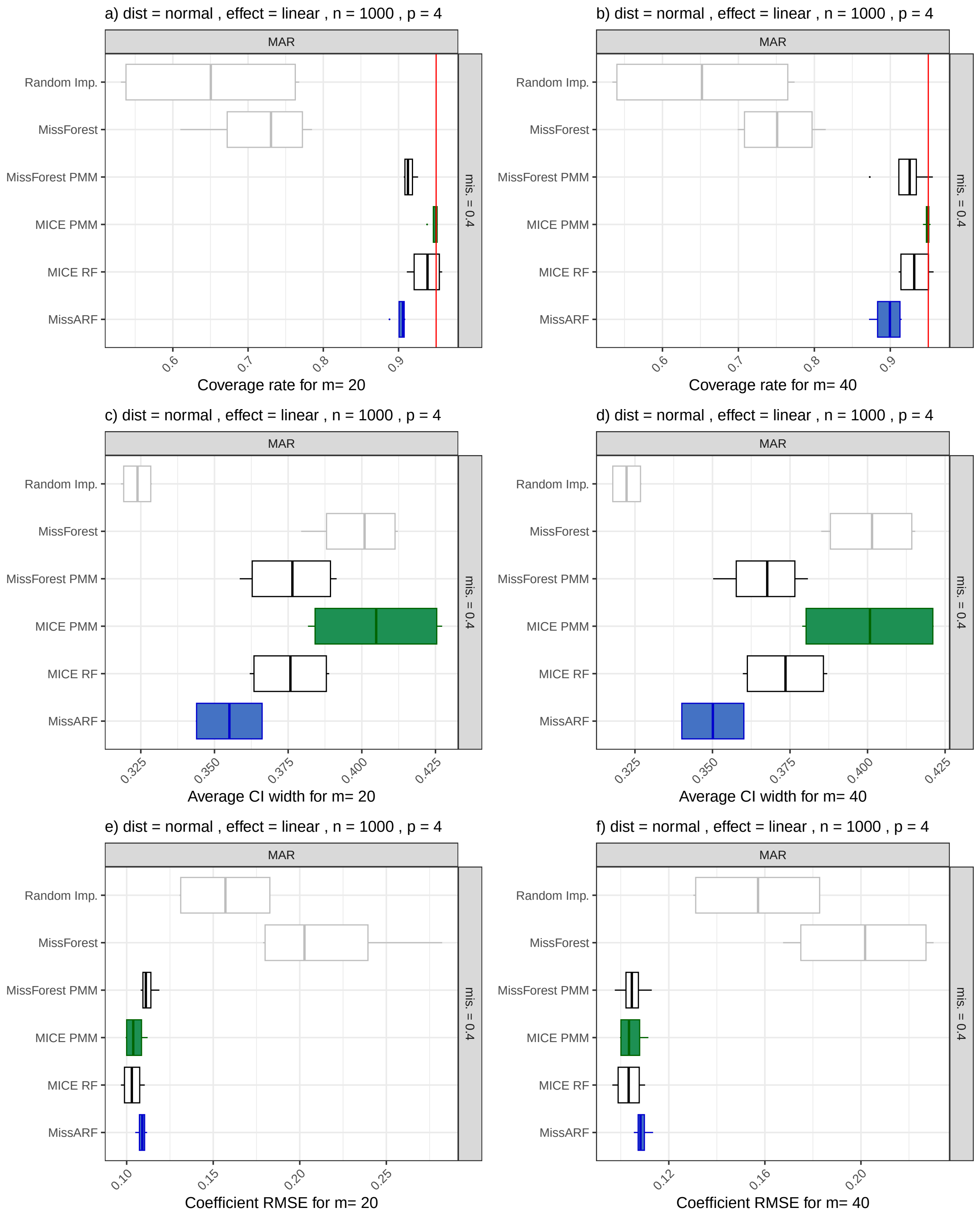

Figure S107: The effect of the number of multiple imputations $m = 20$ (left) and $m = 40$ (right) for a-b) coverage rate, c-d) average confidence interval width and e-f) RMSE for a normal distribution with a linear effect with $p = 4$, $n = 1000$, and 0.4 missingness with a MAR pattern. The boxplots are plotted over the features, with MissARF (blue) and MICE PMM (green) highlighted.

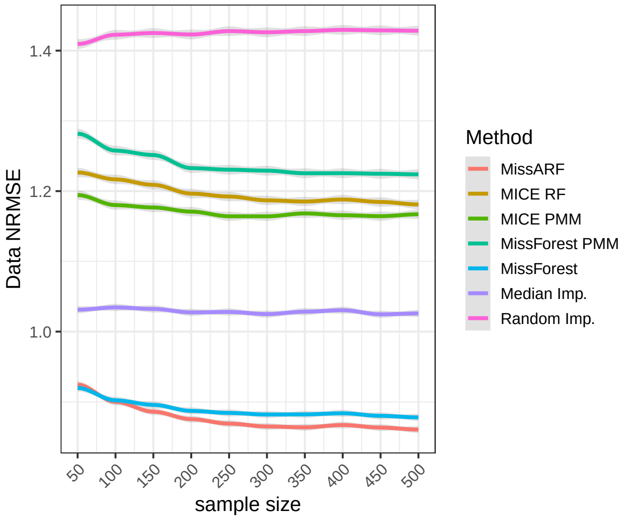

Figure S108: Mean values with the standard error for the NRMSE for sample sizes 50-500 for the different imputation methods. Results are for a normal distribution with a linear effect with $p = 4$ and 0.2 missingness with a MAR pattern over 1000 replications.

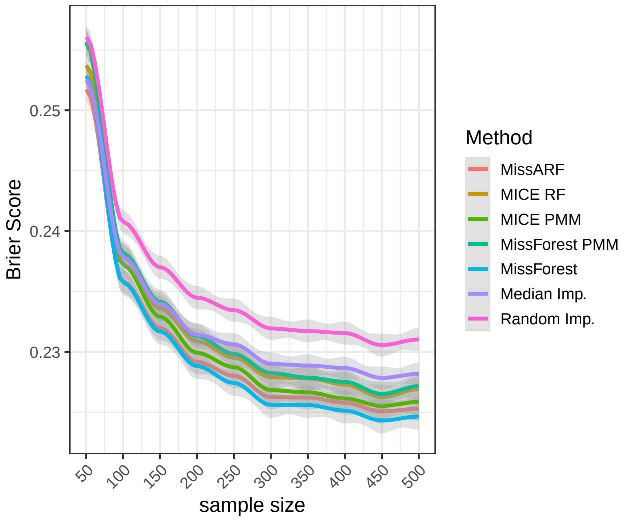

Figure S109: Mean values with the standard error for the Brier Score for sample sizes 50-500 for the different imputation methods. Results are for a normal distribution with a linear effect with $p = 4$ and 0.2 missingness with a MAR pattern over 1000 replications.

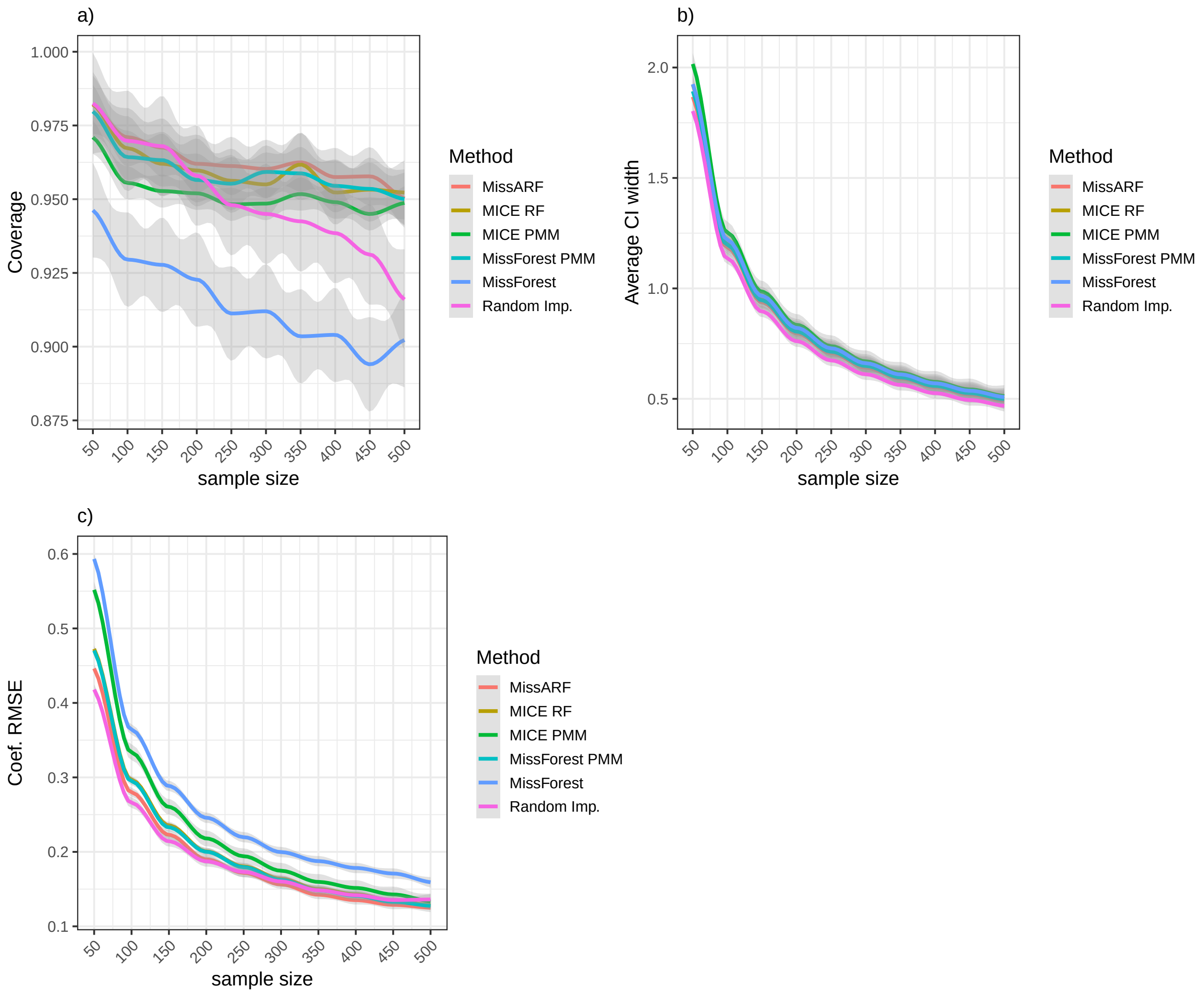

Figure S110: Mean values with the standard error for a) coverage rate, b) average confidence interval width and c) RMSE for sample sizes 50-500 for the different imputation methods. Results are for a normal distribution with a linear effect with $p = 4$ and 0.2 missingness with a MAR pattern over 1000 replications.
